# Light–Dark and Activity Rhythm Therapy (L-DART) to Improve Sleep in People with Schizophrenia Spectrum Disorders: A Single-Group Mixed Methods Study of Feasibility, Acceptability and Adherence

**DOI:** 10.3390/clockssleep5040048

**Published:** 2023-12-04

**Authors:** Sophie Faulkner, Altug Didikoglu, Rory Byrne, Richard Drake, Penny Bee

**Affiliations:** 1 School of Health Sciences, Faculty of Biology Medicine and Health, University of Manchester, Manchester M13 9PL, UK; richard.drake@manchester.ac.uk; 2 Centre for Biological Timing, Division of Neuroscience, School of Biological Sciences, Faculty of Biology Medicine and Health, University of Manchester, Manchester M13 9PL, UK; altugdidikoglu@iyte.edu.tr; 3 Greater Manchester Mental Health NHS Foundation Trust, Bury New Road, Prestwich M25 3BL, UKpenny.bee@manchester.ac.uk (P.B.); 4 Department of Neuroscience, Izmir Institute of Technology, Gulbahce, Urla, Izmir 35430, Turkey

**Keywords:** schizophrenia, psychosis, sleep, insomnia, circadian rhythm disorder, light exposure, occupational therapy, behavioural therapy, CBTi, qualitative

## Abstract

People with a diagnosis of schizophrenia often have poor sleep, even when their psychotic symptoms are relatively well managed. This includes insomnia, sleep apnoea, hypersomnia, and irregular or non-24 h sleep–wake timing. Improving sleep would better support recovery, yet few evidence-based sleep treatments are offered to this group. This paper presents a mixed methods feasibility and acceptability study of Light–Dark and Activity Rhythm Therapy (L-DART). L-DART is delivered by an occupational therapist over 12 weeks. It is highly personalisable to sleep phenotypes and circumstances. Ten participants with schizophrenia spectrum diagnoses and sleep problems received L-DART; their sleep problems and therapy goals were diverse. We measured recruitment, attrition, session attendance, and adverse effects, and qualitatively explored acceptability, engagement, component delivery, adherence, activity patterns, dynamic light exposure, self-reported sleep, wellbeing, and functioning. Recruitment was ahead of target, there was no attrition, and all participants received the minimum ‘dose’ of sessions. Acceptability assessed via qualitative reports and satisfaction ratings was good. Adherence to individual intervention components varied, despite high participant motivation. All made some potentially helpful behaviour changes. Positive sleep and functioning outcomes were reported qualitatively as well as in outcome measures. The findings above support testing the intervention in a larger randomised trial ISRCTN11998005.

## 1. Introduction

Sleep problems are common in people diagnosed with schizophrenia spectrum disorders [1], and there is a lack of specialised sleep input offered within specialist mental health services [2,3].

Sleep problems are linked to, and can cause, significant problems with physical and mental health [4,5,6,7], poorer medication adherence [8], and cognitive impairment [9]. Sleep problems can impact the self-image of people with psychosis and make it more difficult to keep social arrangements and to do paid or voluntary work [10].

Experts identify sleep problems as a modifiable factor affecting outcomes in schizophrenia, arguing it should therefore be treated [9,11,12]. Similarly, people with psychotic illnesses recognise that sleep is important, and they want to sleep better [13], particularly to have better quality, less broken sleep, not just longer sleep [10]. Qualitative research suggests patients prefer non-pharmacological interventions to improve sleep, despite acknowledging that these require effort to successfully engage in [10,14,15].

Circadian dysregulation is much more common in people with schizophrenia than in the general population or those with common mental health problems [16]. Circadian dysregulation can result in reversed (nocturnal) activity timing, irregular rest-activity patterns, or a non-24 h (free-running) sleep–wake rhythm. Among samples with schizophrenia diagnoses, sometimes 50% have significantly delayed or free-running rest-activity patterns [17,18]. Predictive modelling work using longitudinal light exposure and activity patterns suggests that these patterns are driven by reduced light exposure, alongside reduced drive for wakefulness, not by fundamental differences in the circadian clocks of people with schizophrenia, and suggests that increasing light exposure in the daytime could normalise patterns [19].

Changes to light exposure patterns directly affect alertness [20,21], alter circadian entrainment and phase [22,23], and affect mood [24,25,26]. Large-scale Biobank work suggests increased light at night is particularly associated with poorer psychiatric outcomes [27]. There is significant background evidence to suggest which changes are likely to be helpful, but there has been limited work applying advice and behaviour change techniques to support people in altering their own light exposure in the community and then measuring adherence and acceptability.

Sleep problems in people with schizophrenia include insomnia, problems with sleep hygiene [28], excessive caffeine consumption [29,30], parasomnias [31,32], sleep apnoea [33], hypersomnia [34,35], and circadian dysregulation [11,18]. The high level of co-morbidity of sleep problems, and particularly increased circadian dysregulation in this group, was the rationale for the development of a personalisable behavioural intervention that can accommodate co-morbid sleep issues: Light–Dark and Activity Rhythm Therapy (L-DART). L-DART was based on the findings of an expert opinion study with clinical and research topic experts (in sleep, circadian rhythm, occupational therapy, mental health, and psychosis) and service users and carers with relevant personal experience [36]. L-DART includes elements of Cognitive Behavioural Therapy for Insomnia (CBTi), such as time in bed restriction to manipulate sleep pressure (sleep compression), ‘stimulus control’ instructions to increase association of bed with sleep, and sleep hygiene advice [37,38]. L-DART combines these elements with components to alter daytime activity and the home environment, with a focus on improving the regularity and rhythmicity of environmental and behavioural cues (zeitgebers), especially light.

Working on activity routines and environmental modification are often core parts of the role of the occupational therapist [39,40], hence the decision to design the intervention to be delivered by this profession. This study is the first we are aware of to test an occupational therapy intervention to improve sleep in this group. It examines the feasibility of delivery, acceptability, and adherence to intervention recommendations.

## 2. Results

### 2.1. Recruitment and Demographics

#### 2.1.1. Recruitment

Thirty-five referrals were received, and the study was completed within half of the allowed time. Table S1 shows a full breakdown of the outcomes of referrals. Contact was lost with four referred individuals, six declined, eight were ineligible, one was withdrawn by the referrer to refer instead for OSA assessment, and seven were placed on a reserve list as the study waiting list was full at the time of referral. This rate of referral, eligibility, and consent showed it was very feasible to recruit.

#### 2.1.2. Demographics

Of the ten participants, all had a diagnosis of schizophrenia; 80% were male, 100% were White British, 100% were not in paid employment, 100% lived alone, and none lived in residential or supported accommodation. It is difficult to compare such a small sample to population demographics, but more female participants might be expected in a larger study. All were prescribed anti-psychotics, and on average, participants’ psychotic symptoms were relatively well controlled (CGI-SCH overall score at baseline 2–4, mean = 2.9, where 3 = mildly ill). For further details, see Table S2.

#### 2.1.3. Sleep Problem Phenotypes and Participant Goals

See Table 1 for details of the complaints and sleep problem phenotypes of participants from the baseline clinical assessment.

### 2.2. Study Attrition and Data Completeness

We examined attrition from the study separately from adherence to the intervention. There was no attrition. One participant declined to participate in the qualitative interview, and one pre-intervention prospective acceptability brief interview was skipped due to time limitations in the session. Pre-, post-, and follow-up self-report measures were 100% complete, and weekly PROMIS-SD 8a ratings were 60–80% complete (mean 71%). Completeness of passive monitoring data (light and activity recordings) was relatively complete; this is commented on below, and can be seen in Supplementary File S4. Despite some data synching issues, Withings data were mostly complete or nearly complete. We had no inbuilt means of detecting non-wear time, though newer devices commonly have this feature which would improve interpretation in future studies.

### 2.3. Attendance, Delivery, and Adherence Summary

#### 2.3.1. Therapy Session Delivery and Attendance

The therapy protocol prescribed 6–9 face-to-face sessions plus 3–6 phone calls, depending on need. Participants received 4–10 face-to-face sessions (mean 6.9) and 1–10 phone sessions (mean 4.3). Original plans for the split between face-to-face and phone were not adhered to strictly where participants did not want as many phone sessions (*n* = 2, 1–2 sessions), wanted additional phone sessions (*n* = 2, 7–10 sessions), or strongly preferred in person and received additional face-to-face sessions (*n* = 1, 10 sessions).

The planned number of sessions over 12 weeks allowed for some weeks with no session, which allowed for leave and holidays. Face-to-face sessions were more prone to cancellation or postponement (*n* = 17 sessions) than scheduled phone sessions (*n* = 8), but on the other hand, phone sessions were more often not answered without prior warning (*n* = 6) than face-to-face visits (e.g., the client was out when the therapist arrived) (*n* = 2). There were 22 cancellations or postponements for non-COVID-related reasons, five sessions cancelled/postponed, for COVID-related reasons, and 10 face-to-face sessions switched to phone for COVID-related reasons; see Supplementary File S1 for full details.

#### 2.3.2. Intervention Component Delivery and Adherence Summary

It should be noted that it was not expressed to participants that they must accept or adhere to every aspect of the intervention to participate. During recruitment, it was made clear that it was important that participants were willing to make some changes; otherwise, the intervention would not help them, and we suggested they should be open to at least discussing sleep-related behaviours they do not want to change, but that each part was in the end optional. This was important as we accepted referrals where people were not sure if they wanted to change their sleeping location or if they would use a lightbox, for instance, but who still wanted to participate.

**Figure 1:**
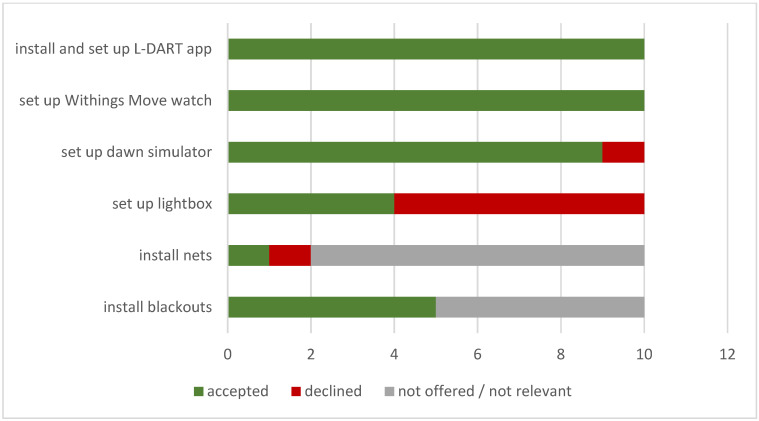
Environmental interventions offered and accepted.

Some interventions were environmental and equipment-based; whether these were recommended and delivered by the therapist and the rate at which each was accepted is summarised in Figure 1.

It is relevant to note that although all participants were offered a light box as part of the therapy, this was not emphasised as an essential aspect, particularly if they were able to go outside more to increase their light exposure (or already went outside a lot). Some participants enrolled in the study had no wish to use a light box and had more interest in other aspects of the therapy. If the study had accepted only those who stated at the outset that they would use a lightbox, there would no doubt have been higher acceptance of the device among participants.

**Figure 2:**
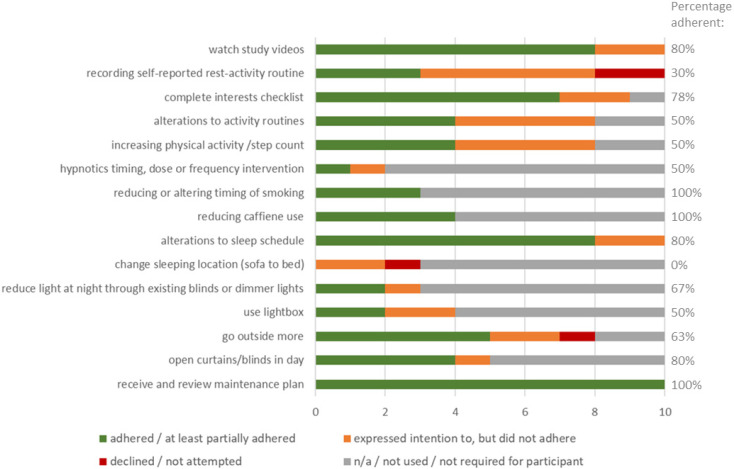
Intervention components delivered, attempted, and self-/therapist-reported adherence.

Discrete and separable behaviour change intervention components are summarised in terms of how many cases they were used, whether there was an attempt or expressed intention to adhere, and the participant’s success in adhering to the intervention component (according to self-report and therapist report). See Figure 2. Where there was sufficient adherence to be judged potentially useful in terms of influencing outcomes, this has been described as partial adherence; if change was so brief or minimal as to not be useful (e.g., trying something once only), this is described as non-adherence. Overall, of these intervention components, when applied, in 67% of those instances, there was adherence.

### 2.4. Intervention Component Delivery, Adherence, Barriers and Facilitators, and Perceived Impact, from Qualitative Accounts, Therapy Notes, and Passive Data

The initial interview and initial goal setting always indicated a number of potentially relevant homework tasks or intervention components. The therapist focused on a combination of the most important areas to modify and the easiest to modify, taking account of service user preference and motivations. The therapist avoided setting more than three homework tasks at the end of a session, and sometimes set fewer depending on complexity or difficulty. The protocol allowed some flexibility already (see therapy content by week, Supplementary File S1), and minor modifications were made to the order of delivery during the study, to allow focusing on the most pertinent issues first, and to avoid overwhelming participants with too much content at once.

To give examples of this personalisation in practice, with participants 1 and 8 with insomnia, we began by looking at the evening wind-down routine and anchoring morning wake time, then moved on to sleep compression, whilst with participants 5, 6, and 7 with non-24 h sleep phase disorder, irregular/reversed sleep timing, and hypersomnia, we initially focused on daytime activity and light exposure, then implemented various personalised changes in sleep timing. With participant 4, we began to address caffeine consumption very early on, whereas for many participants, this was not addressed at all, or where it was a possible or slight contributing factor, it was discussed in detail only later when more pressing concerns had first been addressed.

#### 2.4.1. Explaining the Intervention Rationale to Participants, Educational Content

Some of the intervention rationale was already explained by the Participant Information Sheet and during the recruitment process, so the study sample by definition would not include those who fundamentally disagreed with or disbelieved the rationale.

Most participants watched some or all of the study videos as homework early within the course of therapy, and this enabled faster progression as session time could be spent on assessment, review, and personalised goal setting, rather than didactic presentation of background information. The main barrier to access was when participants had low skills or confidence with technology, for example, “…not confident finding the browser address bar” (therapy notes extract). Sometimes there was an opportunity to supervise and support viewing one or two videos during a session. Giving structured space to make notes helped with ascertaining engagement with the videos, and many participants made use of this. Participants seemed reassured about the idea that it was normal to potentially need to re-watch videos to take everything in. On occasions where participants did not watch the study videos, some of the rationale for instructions was given verbally during sessions; however, this was necessarily more limited than in the videos due to time limitations. Some participants did not watch the educational videos until several weeks into the therapy window, and this could have affected their understanding of the rationale for certain interventions; implementing supported viewing early might be worthwhile.

When the videos had not been watched and goals related to the content contained in them needed to be set, the therapist gave a brief rationale verbally. The stimulus control rationale (use the bed only for sleep, avoid non-sleep activity in bed, and reduce wake time in bed) was always given verbally rather than using a video. As below, this appeared to be accepted, but those who slept on the sofa rather than the bed did not eventually end up changing this behaviour.

#### 2.4.2. Recording Self-Reported Rest Activity Routine

The intervention did not frame this as essential to complete, as rest-activity patterns were tracked by the watch. However, to assess time use and routines, this was requested from all participants. Although it was more often not completed, and occasionally rejected outright, it was possible to proceed without it using other data sources (activity tracking and interviews). A minority of participants completed this rigorously without much encouragement, and in some but not all cases, when completed, it provided useful information that influenced the course of therapy.

#### 2.4.3. Interests Checklist

The results of this were useful in some cases, and usually took minimal time to complete or review, although often multiple prompts were needed before it was completed. Completing together did not take much longer than reviewing answers, so completing this together from the outset may be considered to reduce the number of homework tasks being left with participants in any given session.

#### 2.4.4. Use of Passively Monitored Activity Pattern Plots within the Therapy

Some participants were not confident with technology and found use of the L-DART app difficult and required therapist support. Additionally, there were data synching issues for some participants that required troubleshooting, and in one case, this was not possible to resolve, resulting in missing data (did not transfer from phone to online Withings account and API). Once set up, however, viewing the activity plots together in therapy was useful, enabling a richer picture of baseline patterns, and corroborating or contradicting initial impressions given by participant reports to prompt further clarifying discussion. Participants also reported finding the graphs helpful in terms of reviewing their behaviour or changes in behaviour over time.

#### 2.4.5. Altering Daytime Activity Routines

Usually, but not always, participants bought into the rationale regarding the impact of daytime activity on nighttime sleep; when this was the case, it increased motivation to make changes. Not all participants began the intervention wishing to alter their activity routines, which may differ from the experience of delivering non-sleep-related community occupational therapy, where clients are often referred due to dissatisfaction with their activity routines. A lack of identified interests presented a barrier to progress in other areas of therapy, such as sleep schedule goals, stimulus control, and managing worry. Limited interests can be related to amotivation, reduced pleasure anticipation, or other psychological barriers to engagement, which are beyond the scope of behavioural sleep therapy to adequately address. Further intervention or referral may be required.

Participants’ interday stability (IS) overall slightly decreased during the intervention, but returned to baseline levels during follow-up. This may initially appear counterintuitive as the intervention aims to improve (increase) regularity; however, it seems less surprising when considering that during the intervention period participants began with their old routines, tried out changes in activity patterns suggested, and then may or may not settle on some changes they would maintain ongoingly.

Although only a minority of participants made really significant changes to their daytime activity routines (timing and types of activities), when they did, it appeared impactful on quality of life and sleep. If a large minority can make such changes, this may be sufficient justification for retaining this focus, even if many participants do not make changes. On average, across all participants, the most and least active hours stayed similar. However, in participants 5 and 6 (non-24 h/irregular), there were significant improvements in activity rhythm period (which became closer to 24 h) during and after intervention. Qualitatively, their free-running and irregular patterns were unfortunately not normalised. Participant 7 (with hypersomnia) shifted their daytime and nighttime activity patterns to earlier times (M10 midpoint was 15:50 during baseline, and 13:50 during intervention and follow-up). This did appear as a marked change visually on the activity pattern plot and the participant’s reports. See Supplementary File S4 for passively recorded rest activity plots for all participants.

#### 2.4.6. Increasing Physical Activity Levels or Step Count

This was relatively straightforward to address in most cases, and was mostly met with acceptance by participants. Many individuals reported success in increasing their daytime activity levels, and being able to track progress live with participants by reviewing step tracking in the L-DART app appeared to help. There was no significant difference in daily step counts or activity within the most active 10 h (M10) from baseline to intervention or follow-up. However, qualitatively, one participant took up swimming during the intervention, and another began to regularly attend a gym following the intervention (these activities would not necessarily be picked up via step counts). Withings follow-up step count was missing for one participant due to data synching issues.

The main barriers to increasing physical activity were identifying activities to do, or destinations to walk to and a reason to walk there. If participants were walking to visit someone or doing an activity with someone, they were more likely to complete these plans than solo plans. When there were insurmountable barriers to going outside, it was difficult to increase physical activity if participants were not open to doing home-based exercise (such as using an exercise video). This is a foreseeable challenge for which a pathway or options could be developed. A similar challenge would apply if applying the intervention to those whose movements are restricted by being detained in an institutional environment.

#### 2.4.7. Alteration of Meal Timing

This was always briefly assessed, anticipating that late-night eating might affect some participants, but for these participants, that was not relevant, as none reported night eating. A few participants who previously did not have breakfast began having breakfast or a hot drink as part of their new morning routine.

#### 2.4.8. Hypnotics

As in other research, many participants were already not keen on hypnotics, although some had more mixed views. The main intervention completed was addressing misuse of prescribed hypnotics and divergence from prescriber instructions, though a thorough assessment was needed before this came to light.

#### 2.4.9. Smoking

Participants were often not aware that nicotine is a stimulant; this advice was delivered, and several participants dramatically reduced their nighttime or evening smoking, and/or their smoking total. Reducing smoking was not a core element of the intervention and was not pursued with all participants who smoked, but rather where this appeared to be a more significant contributor or where participants seemed more interested in reducing smoking. Sometimes, stopping smoking during the night was reported to be the main thing/one of the main things that improved sleep.

#### 2.4.10. Caffeine

Although participants knew caffeine was a stimulant, many were not aware of how much above-recommended levels they were consuming. Translating the number and type of caffeinated drinks into an approximate value in caffeine mg and providing this with a comparison to recommended limits surprised and motivated participants to reduce their use. Homework to try decaffeinated versions was completed and resulted in changes in caffeine consumption, at least in the mid-term.

#### 2.4.11. Addressing Comorbidities

There was a moderate amount of time spent during therapy discussing and troubleshooting the impact on sleep of a range of physical health conditions, and several participants either had OSA or were identified as being at high risk for OSA during assessment. Difficulties switching off due to worry, anxiety, paranoia, or hearing voices were identified as an area to address in a large minority of cases (this reflects the relatively stable community sample and may be a larger feature in other settings).

#### 2.4.12. Alterations to Sleep Schedule

There were a large number of goals set regarding sleep schedule alterations, the nature of which varied depending on the sleep problem type. Overall, considering these can be difficult to adhere to, participants did well, and most made significant changes to their sleep schedule (after revisiting, if not immediately), some of which were sustained to the end of therapy and at follow-up. Various changes can be seen visually on activity plots, including participant 8 who increased consistency of sleep timing on weekdays and weekends (reduced sleeping-in and increased activity on weekend days), and participant 3 who tried out a very early wake time (6 am) during intervention, then settled on a consistent wake time of around 8 am by the end of the intervention period and during follow-up. Even those two participants, categorised as mostly not adhering in Figure 2 above, attempted changes in their sleep schedule briefly or intermittently, but which were not sufficiently sustained to be expected to improve sleep/sleep patterns and not continued by the end of therapy.

#### 2.4.13. Sleep Compression and Time in Bed Reduction

Reducing the time in bed window was used in two ways—where the sleep window was excessive and wanted to be permanently reduced, and where increased sleep pressure was desirable to improve sleep onset and maintenance as per CBTi. Changes were well adhered to; some found it difficult but adhered anyway. For one participant with hypersomnia, this was the main focus: to sleep for fewer hours and feel alert in the daytime. This was undertaken gradually in 15 min increments and resulted in a much shorter self-reported sleep and passively monitored inactive (assumed sleep) period from ~14 h during baseline to ~11.5 h during follow-up (see Supplementary File S4). This was accompanied by increased, rather than reduced, qualitatively self-reported daytime alertness.

Although intervention development work highlighted avoiding naps as a key area, no participants reported frequent napping, and those who occasionally napped reported this was easy to stop.

#### 2.4.14. Stimulus Control (‘Go to Bed Only when Sleepy’, ‘Use the Bed Only for Sleep’, and ‘the 15 Min Rule’)

During intervention development there was significant resistance and bad experiences described regarding this [36], however the rationale was well accepted by participants, mostly adhered to, and described as helpful by many.

The greatest challenge was using the sleeping surface only for sleep. In participants who were not sleeping in their bed, one did not have a usable bed, and two of three tried sleeping in their bed for periods, but reverted to the settee by the end of therapy.

#### 2.4.15. Regular Rise Time

This idea was mostly embraced, and participants reported efforts to adhere to a regular rise time and at least some success. Participants with insomnia or mixed sleep problems appeared to find this easier to adhere to than the two participants with non-24 h and irregular sleep timing, who tried repeatedly but did not sustain a regular rise time.

#### 2.4.16. Altering Overall Sleep Timing (Bed Time and Rise Time)

This was a key focus for the participants with hypersomnia, late rise time, non-24 h, and irregular sleep timing. All these participants engaged fully in a collaborative process of negotiating target times, considering both their ideal target times and what the therapist advised was most achievable. In one case, a participant would have ideally preferred to improve their sleep whilst remaining mostly nocturnal; in this case, they accepted the explanation and rationale for why this was unlikely to be possible, although some ambivalence regarding conflicting priorities and goals may have remained. Practitioners should consider how to approach those with sleep goals that are in conflict with some of the premises of the intervention, including the extent of educational and motivational work at the outset, as conflicting priorities were not operationalised as an exclusion criterion.

#### 2.4.17. Dawn Simulator/Wake up Light

There were overwhelmingly positive experiences reported of the gradual awakening with light and nature sounds instead of a buzzer. Some reported that this improved their ability to get up in the morning; others just described it as more pleasant. Once set up, its use did not usually add any burden in the form of specific homework tasks for participants to complete. For those who did not describe the benefit, this was because they were already awake at the time it was set to go off due to early wakening or erratic sleep timing.

#### 2.4.18. Increasing Daytime Natural Light Exposure

We focused on going outside more, but it was a difficult behaviour to change in most cases. Despite the fact that several participants demonstrated having bought into the rationale for this, qualitative reports suggested most did not make major changes to their time outdoors. Wrist bright light (>500 lx) exposure duration and average light exposure were correlated with seasonal daylength (unsurprisingly) and showed no significant difference from baseline to intervention or follow-up. MotionWatch wrist light measures are missing for three participants’ intervention and two participants’ follow-up, probably reflecting the low acceptability of wearing a second device and suggesting a need for all features in one device or increased miniaturisation for ease of wear.

Opening the curtains or blinds in the daytime was an easy behaviour to change once privacy concerns were addressed with net curtains if needed. Participants reported opening their curtains routinely once in the habit. Showing participants light metre readings of the brightness of light indoors versus outdoors and in different locations (near and far from the window) was fast to deliver, was well received, and surprised participants regarding how much darker it was indoors even when it appeared ‘light enough’. Passively monitored living room overall light exposure and bright light exposure duration usually increased during intervention. They significantly increased and stayed high in follow-up for participant 6 with irregular rhythm (who’s IS also improved during the study).

Amongst two rooms and 60 measurement time points, MotionWatch wall measures are missing for 8 timepoints (13.3%); data were missing at baseline × 2, intervention × 2, and follow-up × 4. All were due to device malfunction (data failed to record/download), except for × 2 follow-up measurements, which were due to the participant declining the follow-up visit (completed outcome measures remotely). Collecting ambient light levels using wall-mounted devices was shown to be low-burden and readily accepted when explained adequately. Device malfunction levels reported in this study reflect that an older version of the firmware was being used on our MotionWatch 8 due to budget constraints, which, if updated, should perform better. For wrist-worn and environmental light pattern plots, see Supplementary File S4.

#### 2.4.19. Light Box

Some declined the light box before trying it, and this was sometimes not pushed if it was not the highest priority, for instance, in those with less circadian abnormality. Some tried it and discontinued it after perceiving no immediate effect. In one case, making other changes at the same time made it difficult for the participant to determine the cause of improved alertness. One participant felt more alert with use and continued regularly. It seems likely that introducing this at the same time as other components and framing it as an option, not a core element of intervention, reduced uptake compared to other studies where light box use was the main/sole focus.

#### 2.4.20. Reducing Light at Night

It was necessary to advise some participants to turn the light off at night for sleep, or to close existing blinds, as the effect of light on sleep at night was not apparent or known to them. This was often accepted, as there was no barrier for them to doing this other than not knowing it might help. Fear of the dark was a reason not to wish to turn the light off fully, in which case it was very important to have a dim light option to offer as an alternative to a full light (the dim red midnight-light setting on the dawn simulator fulfilled this function). There was no meaningful pattern in the change in nighttime light levels from passively monitored light, although qualitatively, participants reported improved sleep maintenance from not being woken up by bright light in the morning or intermittent bright light from passing traffic.

### 2.5. Barriers and Facilitators to Adherence (Overall)

#### 2.5.1. Expectations and Motivation

Many participants had low expectations of success, due to the length of time they had had sleep problems and their severity, or because other approaches had not worked: “I’ve been through a lot of stuff, you know, trying different things, and it really hasn’t made any difference, so yeah, you know I’m not overly confident”. However, participants expressed or showed high motivation despite this, including, in some cases, initiating changes before starting the intervention (see qualitative data extracts, Supplementary File S3). Through keenness, participants would also sometimes try off-protocol additions, such as reducing the sleep window further than instructed and trying lavender drops.

#### 2.5.2. Format, Scheduling and Duration

Intervention delivery was blended (face-to-face and by phone) and adjusted to suit participant preferences. Some participants had no preference regarding the format of contact, whilst others found in-person contact preferable, citing that it was more engaging, or easier for things to be demonstrated/explained. Some spoke positively about the flexibility to reschedule with phone calls, and phone calls did enable the replacement of cancelled contacts at shorter notice. There were various positive comments about the flexibility of the therapist in offering suitable times for participants, suggesting this may be an important feature to retain in the future. Participants suggested the duration and amount of contact were about right, and this was also reflected in the fact that the planned content was usually able to be covered within the sessions offered (this was the case even with some weeks of no contact where the therapist or participant were unavailable due to illness or holidays).

#### 2.5.3. Interpersonal Factors

Some participants noted struggling with meeting new people but opening up after getting to know the therapist better. In this study, the therapist set up the appointments, took consent, and completed baseline measures and device set-up. Having this contact take place before the initial interview may have improved rapport, allowing for a reasonable assessment. In a future study, the protocol should be arranged such that the initial assessment is not the first contact with the therapist. There was only one occasion noted where questions asked were problematic interpersonally, which was when asking about daytime routines and activities was seen as invasive (when the relationship with sleep was not properly conveyed).

Participants spoke positively about the therapist, and when reasons were given, these were most often related to explaining things well, being clear, and making concepts understandable, and sometimes to listening and taking enough time.

#### 2.5.4. Impact of Social and Physical Environment, including COVID Pandemic

Participants’ social environment was helpful when it provided reasons to go out or maintain commitments. Equally, it was possible for friends or family to be a bad influence on routines or a source of stress. The impact of the physical environment was significant, both in terms of the bedroom environment and the local area. Noise, physical comfort, and pets affected sleep and were not easy to modify. Staying at others’ houses impacted intervention delivery and adherence (e.g., missed appointments, different routines).

There were few COVID measures in place restricting activities during the study, and worries about COVID were not cited as a barrier to pursuing social or activity-related goals by any participants. Masks were worn during appointments; participants reported no problems with mask wearing or any other infection control measures. Some therapy time was lost due to COVID infection or isolation, and others switched to phone contact from in-person contact, and therapy goal progress was understandably affected when participants contracted COVID.

#### 2.5.5. Linking with Other Care Providers and Services

A moderate amount of time was spent liaising with sleep services and participants’ mental health teams. Waiting for information, replies, or contact details could cause a delay in otherwise useful communication. Having better established channels of communication (often simply knowledge of who to contact and how) would speed this up. This could be achieved with larger-scale work where more participants are seen from each locality and processes become familiar to staff, and prior contact with services before commencing could be helpful.

### 2.6. Acceptability

#### 2.6.1. Quantitative Acceptability Ratings

The statements “I like L-DART (so far)”, “I am satisfied with L-DART (so far)”, and “I would recommend L-DART to someone else with a similar problem” were all rated on average between ‘agree’ and ‘strongly agree’, on a scale of 1 (strongly disagree) to 5 (strongly agree) (mean (SD) = 4.4 (0.5), 4.3 (0.5), 4.4 (0.7)).

#### 2.6.2. Qualitative Acceptability Data

Congruent with the multiple-choice ratings, qualitative comments were positive. Participants said L-DART was worthwhile for others to try, and that although it took effort and was difficult at first, participants described it as worth the effort.

Participants often discussed what they had learned about sleep and circadian functioning, and valued what they described as a better understanding. This was often the case even where this had not translated into much improved sleep, and participants often expressed that they could use this knowledge in the future: “[explanation of circadian rhythm] has been digested by me, but I’m just not using it at the moment, but I do feel that it’s been of value”.

### 2.7. Adverse Events and Adverse Effects

#### 2.7.1. Unrelated Adverse Events

There were no hospitalisations, deaths, or other SAEs. There were two cases of COVID-19 infection and two short-term worsenings of mental state, neither of which required inpatient admission or crisis team intervention (one during treatment, one during follow-up). Other exacerbating factors were cited by participants, though case reviews suggested no evidence of relatedness to the intervention.

#### 2.7.2. Adverse Effects

Three participants developed a skin rash where the Withings silicone watch strap was in contact with their skin, all in the summer. In one case, this recurred on the other wrist when the watch was moved. In all cases, this resolved with moving or temporarily removing the watch, and advice to ensure skin is fully dry underneath after showering. Two participants accepted an alternative watch strap (fabric and leather options were offered).

### 2.8. Outcomes

#### 2.8.1. Self-Reported and Therapist Rated Outcomes

**Figure 3:**
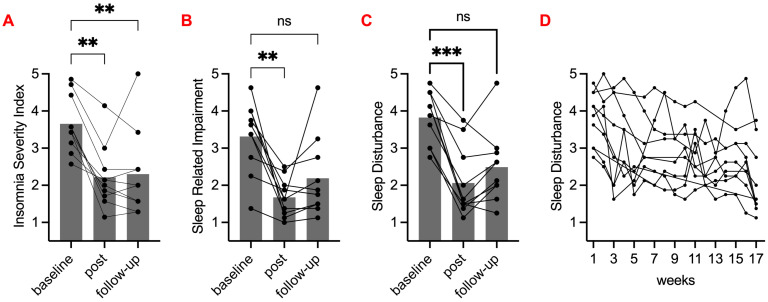
Sleep quality before, during, and after intervention. (**A**) Insomnia Severity Index (ISI). (**B**) PROMIS-SRI 8a (Sleep-Related Impairment). (**C**) PROMIS-SD 8a (Sleep Disturbance). (**A**–**C**) Grey bars show mean values of baseline, post-intervention, and follow-up scores. Black circles show participants’ individual scores, and lines represent a participant. Friedman tests with Dunn’s multiple comparison against baseline were performed (ns *p* > 0.05; ** *p* < 0.01; *** *p* < 0.001). (**D**) PROMIS-SD 8a (Sleep Disturbance) score over study weeks (baseline and intervention period). Black circles show participant individual scores, and lines represent a participant.

**Figure 4:**
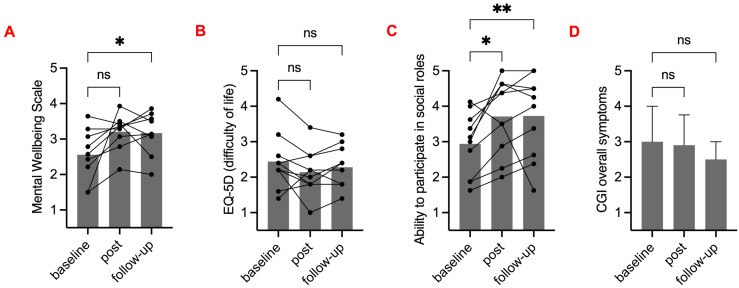
Mental health, wellbeing, and social participation before, during, and after intervention. (**A**) Warwick–Edinburgh Mental Wellbeing Scale (WEMWBS). (**B**) EQ 5D-5L (5-level EQ-5D version, EuroQol). (**C**) PROMIS-AP 8a (Ability to Participate in Social Roles and Activities). (**A–C**) Grey bars show mean values of baseline, post-intervention, and follow-up scores. Black circles show participant individual scores, and lines represent a participant. (**D**) Clinical Global Impression-Schizophrenia (CGI- SCH) overall score mean and 95% confidence intervals. (**A–D**) Friedman tests with Dunn’s multiple comparison against baseline were performed (ns *p* > 0.05; * *p* < 0.05; ** *p* < 0.01).

ISI scores were significantly lower (less sleep problems) at post-intervention and 6 months follow-up compared to baseline; PROMIS-SD and PROMIS-SRI were significantly lower (less sleep disturbance and sleep-related impairment) at post-intervention but not at follow-up. WEMWEBS, EQ-5D, and CGI-SCH were not significantly different at any time point. PROMIS-AP was significantly higher (better functioning) at post-intervention, and the difference was highly significant between baseline and follow-up. See Figure 3 and Figure 4.

#### 2.8.2. Qualitative Reports on Outcomes

Congruent with the self-report ratings, participants described sleep improvement in various domains, although they often noted that sleep was still disturbed and not completely normalised. Participants described varied improvements in specific sleep parameters, including sleep maintenance, sleep quality, having fewer nightmares, and longer sleep. Most often, though, participants described improvements in sleep-related functioning:

“…so much more awake and alive compared to how I was.”

“…there’s more time for going out and making appointments for in the mornings, cos one time I could never make an appointment anywhere in the morning. But at this time now I can make more appointments and I can go out more. It’s changed my life, this therapy.”

## 3. Discussion

This study found that it was easy to recruit participants with a schizophrenia spectrum diagnosis who wished to receive L-DART and had relevant sleep problems from specialist mental health services. It was possible to deliver L-DART in the community in the time allowed and to adapt the intervention to fit a range of circumstances, preferences, and sleep problem phenotypes. It was identified that more detailed pathways for L-DART’s application to certain circumstances should be formalised before larger-scale testing (including for non-24 h sleep phase disorder and for when participants are unable to go outside).

L-DART was acceptable to recipients, as reflected in satisfaction ratings and qualitative accounts, and as can be inferred from good attendance at therapy. This agrees with separate survey work that found the prospective/hypothetical acceptability of L-DART was high in a wider severe mental illness sample (*n* = 190 service users, *n* = 147 staff) [41]. Overall, participants were motivated to attend sessions, showed good engagement with therapy discussions and homework tasks, adhered to either some, or many, recommendations (for those summarised quantitatively, 67%), and all continued with therapy until the end. This compares positively to rates of adherence in sleep-related intervention studies (65.5%) and rates of intervention dropout (14–40%), summarised in the meta-analysis of CBTi intervention in other groups [37]. This contradicts views expressed by some staff that patients with psychosis would not be motivated to improve their sleep [42,43] and better agrees with the views of people with schizophrenia, who said they would be highly motivated to improve their sleep [36].

Adherence to the individual recommendations of L-DART varied, and understandably, some changes appeared more difficult than others to implement. Two elements that were particularly poorly adhered to were logging daily activity and sleep habits on paper (3/10), and changing the main sleeping location from the living room to the bedroom (0/3). As there was passive monitoring of activity patterns and discussion of these during sessions, there was not much negative impact when written self-report activity logging was not carried out, so this element could be considered for removal or made more optional. Regarding sleeping location, it will be considered whether the behaviour change techniques used in relation to this could be optimised, as for comfort and behavioural association reasons, it is probably usually important. Restricting time in bed and alterations to sleep schedules were better adhered to than expected in development work (which suggested this would be a very difficult element for participants) [36], whilst alterations to daytime activity were somewhat less adhered to. This might reflect that sleep pressure and timing were explained in the video-based educational content, more so than the impact of daytime activity on sleep, which was instead only explained verbally; additional content could be included in a future iteration.

This study was not randomised, nor was it powered to examine safety. However, outcomes measures, qualitative accounts, and adverse effects records are promising. There is a substantial existing evidence base supporting various of the components included, such as time in bed restriction and sleep scheduling [38,44], and the positive and negative health impacts of different patterns of light exposure [27,45,46,47,48]. Furthermore, behavioural sleep interventions with some overlapping components have shown promising results in mixed psychiatric inpatients [49], outpatients [50], participants with persistent delusions and hallucinations [51], and young people at high risk for psychosis [52]. So, although the intervention is novel, what we know of sleep and circadian regulation and applied sleep interventions suggests that if properly delivered and adhered to, the L-DART intervention would be expected to improve sleep and wellbeing. The speed and ease of recruitment suggest sleep intervention is a priority for service users with schizophrenia spectrum diagnoses, agreeing with other evidence [14,36,53], and suggest sleep interventions for this group should be a research and clinical priority in the future. L-DART has the particular advantages of being co-developed with lived experience input, applicable to a range of overlapping sleep disorders, and delivered by a relatively inexpensive staff group compared to some psychological therapies. These features, the existing background evidence, and these promising preliminary findings support further testing of L-DART on a larger scale.

## 4. Materials and Methods

### 4.1. Study Population

Included participants were adult mental health service users with a diagnosis of non-affective psychosis (Schizophrenia, Schizotypal disorder, Delusional disorder, or Schizoaffective disorder), and a self-reported sleep problem (initiation, maintenance, timing, duration, or refreshingness).

We excluded people with untreated severe sleep apnoea, whose primary complaint was sleep apnoea or parasomnia, or whose condition or circumstances were too unstable to permit useful participation in L-DART. See Supplementary File S1 for full inclusion/exclusion criteria.

#### Recruitment Method

Participants were recruited via clinical gatekeepers in NHS mental health services and directly via posters and leaflets in patient areas. Participants were given the Participant Information Sheet in their preferred format, with a link to the study description video, and had at least a few days to consider this before consenting (usually much longer). Written informed consent was obtained before any study procedures commenced. The second cohort (winter start) was on a waiting list, so consent was reviewed verbally before visiting, in case things had changed, and written consent was taken at the start of the first visit.

### 4.2. Ethical Approval and Study Commencement

This study received NHS ethical approval on 13 March 2020 from the North West—Greater Manchester South Research Ethics Committee (REC reference 20/NW/0059). It opened to recruitment one year and two months later at its first site on 13 May 2021, due to the COVID-19 pandemic.

### 4.3. Data Collection

See Supplementary File S1 for visual timelines of measures and activities by study week.

#### 4.3.1. Season of Data Collection and Intervention

It was scheduled for some participants to receive the intervention in the summer and some in the winter (as the season may affect light-related intervention adherence or outcomes). The first cohort began participation in May–June 2021, and the second cohort began in December 2021–January 2022.

#### 4.3.2. Routine Clinical Data

We collected routine therapy data in the form of therapy notes, correspondence, and therapy documents (e.g., goal summaries and maintenance plans) and logged session attendance, cancellations, and re-schedules.

#### 4.3.3. Adverse Events and Adverse Effects

We monitored adverse effects via therapist assessment during visits, as well as open questions, and a checklist was used, incorporating the known possible adverse effects of light therapy and sleep restriction. This was read to participants in full initially, then abbreviated once they were familiar. The qualitative interview (conducted by a separate researcher) also probed undesired effects or experiences. See Supplementary File S1 for the assessment and escalation procedure for adverse effects.

#### 4.3.4. Custom Measures

We collected demographic information, therapy satisfaction ratings, and repeated some of the questions from the initial assessment post-therapy to quantify changes in variables such as total sleep time; see Supplementary File S1 for details.

#### 4.3.5. Qualitative Data Collection

We collected qualitative interview data on acceptability prospectively and retrospectively. Pre-intervention acceptability interviews (~5 min) assessed expectations for perceived effectiveness and self-efficacy and were completed by the chief investigator. Post-intervention interviews (~60 min) were conducted by an experienced qualitative researcher with personal experience of psychosis and of receipt of mental health services and explored acceptability, barriers and facilitators to adherence, and perceived intervention impact (see qualitative Supplementary File S2 for interview topic guides).

#### 4.3.6. Standardised Outcome Measures

We collected measures proposed as outcomes to be tested in a future randomised trial, which had been selected by the study team and endorsed by patient and public involvement (PPI) contributors:

- Insomnia Severity Index (ISI) [54,55];
- PROMIS-SD 8a (Sleep Disturbance) [56,57];
- PROMIS-SRI 8a (Sleep-Related Impairment) [56,57];
- Warwick–Edinburgh Mental Wellbeing Scale (WEMWBS) [58];
- EQ 5D-5L (5-level EQ-5D version, EuroQol) [59];
- PROMIS-AP 8a (Ability to Participate in Social Roles and Activities) [60];
- Clinical Global Impression-Schizophrenia (CGI- SCH) [61].

#### 4.3.7. Passive Data/Objective Data

We collected Withings Move step count data throughout, which was used both within therapy and in our analysis. We also collected CamnTech MotionWatch 8 accelerometry and light exposure (lux) recordings during specific periods (wrist worn and measuring environmental light in living and sleeping areas).

### 4.4. Intervention Content and Format

All participants received Light–Dark and Activity Rhythm Therapy (L-DART), delivered by the Chief Investigator (CI), an experienced mental health occupational therapist. L-DART was scheduled to be given over 12 weeks in 6–9 face-to-face sessions plus 3–6 phone calls as needed (‘hybrid’ delivery was planned from the outset and was not a pandemic-related adaptation).

#### 4.4.1. Assessment, formulation, and goal setting

L-DART begins with a three-week baseline period of passive monitoring of rest-activity patterns and light exposure. This is followed by a structured clinical interview evaluating the following:

Sleep priorities and participants’ desires from therapy;

Sleep behaviours, sleep duration, and timing;

Sleep environment, wider physical and social environment;

Symptoms of parasomnias and other sleep disorders;

OSA risk (using the BMI-NECK algorithm [62]);

Contraindications for light therapy;

History of sleep complaints;

Mental and psychological wellbeing and physical health;

Perceived impact of prescribed medications on sleep;

Use of hypnotics;

Use of alcohol, drugs, nicotine, and caffeine;

Roles, activities, and routines;

Perception of occupational balance (self-care, productivity, active leisure, and passive leisure).

Based on the passively recorded rest-activity data and the interview, an initial formulation is then drafted by the therapist for the next session, identifying strengths, issues, proposed target areas, potential challenges, and supports. This is edited and agreed upon with the client before any goals are set.

#### 4.4.2. Intervention Components and Delivery

L-DART addresses the following core areas:

- Light exposure patterns across the day;
- Nature, balance, and timing of activities;
- Sleep schedule modifications;
- Optional components (when relevant) include:
- Reducing or changing the timing of the use of caffeine, alcohol, illicit drugs, and over-the-counter medications;
- Altering or regulating the timing of sleep-inducing prescribed medications;
- Addressing meal timing;
- Addressing nightmares;
- Methods to reduce or avoid daytime naps.

Educational content was delivered through a series of short videos to be watched between sessions and discussed with the participant. Paper copies of intervention materials were provided in phases rather than in bulk at the start and collated in a file in the participant’s home. Worksheets such as activity recording templates or interest checklists were left with participants for completion or completed in session. Negotiated homework tasks were written down and left with participants. For an overview of therapy activity per week, see Supplementary File S1.

Rest activity pattern data from the Withings Move watch were used throughout for the participant and therapist to track progress during sessions and were viewable live on the participant’s smartphone or a loaned study phone. We did not use the data-view of the Withings HealthMate app but instead developed a study app that presented step/movement data as a rest-activity plot without summarising assumed sleep [63]. For an illustrative screenshot, see Supplementary File S1, Figure S3.

Some intervention materials were reviewed and commented on by participants in the expert opinion study [36]. The name and branding of L-DART were developed with input from the study’s own PPI group as well as input from another psychosis Service User Reference Group.

### 4.5. Analysis

Recruitment rates, reasons for ineligibility, and therapy-related reasons for declines are presented, as these could affect future implementation and testing [64]. Study attrition, demographics, attendance, delivery, and adherence are summarised using descriptive statistics.

Qualitative data were analysed using a framework approach in Nvivo 12 software. Three participants’ transcripts were separately analysed by the qualitative interviewer researcher and by the CI. An a-priori coding frame was developed containing descriptive codes, and further analytic codes were developed during analysis by both these researchers. Qualitative results have then been compared to quantitative results on the various topics presented below.

We used quantitative analysis (GraphPad Prism version 9, Boston, MA, USA) to compare self-report outcome measure scores and CGI-SCH between baseline, post-intervention, and follow-up, where there were no missing data. We used a non-parametric repeated measure statistical test: the Friedman test with Dunn’s multiple comparison against baseline. For Withings Move step counts and CamnTech MotionWatch 8 accelerometry and light exposure recordings, the data cleaning pipeline is described in Supplementary File S1. Descriptive statistics, non-parametric circadian variables, observed daily periods, and time-of-day distributions were calculated [19,53].

Sleep problem phenotypes for which the intervention is more or less adhered to or more or less effective are examined qualitatively, as quantitative data are underpowered to detect differences between groups.

Time and difficulty to deliver components and for participants to adhere (such as how often they are repeated and how much of the participant’s available effort and attention appear to be needed to complete the homework) are commented on, in case efficiencies or problems can be identified.

Outcome measures (both content probed and individual change) are compared to the qualitative data regarding what changes participants deemed important in order to inform the selection of measures and the selection of a primary outcome for future studies.

## Figures and Tables

**Table 1:** Sleep problems and treatment goals of participants.

Participant number	Sleep Problem Phenotype	Complaints/Treatment Goals to Change									
		Sol	Agitation and Psychological Distress in the Night	Sleep Maintenance	Sleep Quality/Refreshingness	Nightmares or Bad Dreams	Physical Sensations or Pain Around Sleep	Short Sleep Duration	Excessive Sleep Duration	Inappropriate Sleep Timing	Daytime Alertness
1	Insomnia	x	x	x							
2	Poor sleep hygiene/pain	x		x		x	x				
3	Insomnia/poor sleep hygiene/poor sleep environment			x		x		x			
4	Excessive caffeine/OSA (on CPAP)			x	x		x				
5	Non-24 h sleep phase disorder	x						x	x	x	
6	Irregular/reversed sleep timing	x	x		x	x					
7	Hypersomnia								x	x	x
8	Insomnia	x		x	x						
9	Paradoxical insomnia/daytime inactivity	x			x			x			
10	Poor sleep hygiene/poor sleep environment/pain	x		x	x						x

## Data Availability

Aggregated data, plots, and qualitative data extracts are available in the supplementary material. Other data are available on request from the corresponding author. Not all data are publicly available due to the increasing potential identifiability of participants.

## Supplementary Materials

The following supporting information can be downloaded at: https://www.mdpi.com/article/10.3390/clockssleep5040048/s1, File S1: Supplementary Tables, Figures, and information; File S2: Qualitative topic guides; File S3: Qualitative data extracts; File S4: Passively monitored rest-activity and light exposure plots.
