# Supplementary material for: Light–Dark and Activity Rhythm Therapy (L-DART) to Improve Sleep in People with Schizophrenia Spectrum Disorders: A Single-Group Mixed Methods Study of Feasibility, Acceptability and Adherence
Source: Clocks Sleep. 2023 Dec 4;5(4):734–54. doi: 10.3390/clockssleep5040048 (PMC10742153; doi:10.3390/clockssleep5040048)
Supplement: Supplementary file 1 [file clockssleep-05-00048-s001.zip › clockssleep-2657004-supplementary-S1.pdf]

Supplementary Tables, Figures, and information

Contents:

Additional information—methods
Participant inclusion and exclusion criteria (full detail)
Adverse events reporting procedure
Table S1: Data collection per week of participation
Custom measures
Figure S1: Research participation timeline
Figure S2: Therapy content per week
Figure S3: L-DART app, rest-activity pattern plot display
Objective data cleaning

Additional information—findings
Table S2: Outcomes of referrals
Table S3: Demographics of participants
Additional details on why sessions did not occur or were rescheduled

Additional information—methods

Participant inclusion and exclusion criteria (full detail)

Inclusion:

- Adult service users of the two participating mental health trusts in the northwest of England.
- With a diagnosis of non-affective psychosis (Schizophrenia, Schizotypal disorder, Delusional disorder, and Schizoaffective disorder (ICD-10 F20, F21, F22, F25, F28/DSM 295.*, 297.1)).
- Who express dissatisfaction with their sleep (length of time to fall asleep, amount of sleep, subjective sleep quality, broken sleep, unrefreshing sleep, difficulty waking up, unsatisfactory timing of sleep).
- Who are interested in receiving L-DART.

Exclusions:

- Untreated sleep apnoea (Apnoea Hypopnoea Index (AHI) Index >20, or symptomatic)
- Primary complaint was sleep apnoea, sleep-related movement disorder, or parasomnia.
- Diagnosis of narcolepsy or Rapid Eye Movement (REM) sleep behaviour disorder.
- Co-morbid learning disability, dementia, or moderate to severe neurological impairment.
- Alcohol or substance dependence (unsuitable if using heavily every day and/or not able to be sober for sessions).
- No fixed abode.
- Condition too unstable or acute (defined as follows: 1a. Change of medication within the last 1 month; 1b. Discharged from hospital within the last 1 month; 1c. Current inpatient or acuity of illness requiring home treatment team; 1d. Actively suicidal (expressing suicidal plans or intent); or 1e. Risk to others prevents lone visiting.)
- No capacity to give informed consent.

Adverse events reporting procedure:

Adverse events (AEs) were discussed routinely at intervals with the therapist researcher’s clinical academic supervisor, Professor Richard Drake. There was a reporting and escalation procedure for the assessment of more serious adverse events (whether intervention-related or not) to be discussed immediately with Richard Drake and then with an independent expert (Professor Bill Deakin). Serious Adverse Events (SAEs) were to be reported to the Research Ethics Committee if they were intervention-related and unexpected.

Table S1: Data collection per week of participation

	week	1	4	5	6	7	8	9	10	11	12	13	14	15	17	29	30
	**Measure/Period**	**B**	**therapy window**												**P**	**FU**	
Standardised	ISI	x													x	x	
	PROMIS-SD 8a	x	x	x	x	x	x	x	x	x	x	x	x	x	x	x	
	PROMIS-SRI 8a	x													x	x	
	WEMWBS	x													x	x	
	EQ 5D-5L	x													x	x	
	PROMIS-AP 8a	x													x	x	
	CGI-SCH	x													x	x	
	MotionWatch 8 (MW8) accelerometry and light	[------]												[------]		[------]	
	Withings Move activity watch	[--]															
	Environmental light, MW8	[---]														[------]	
Routine data	Initial assessment		x														
	Self-reported activities	-----------------------as required-----------------------															
	Self-reported sleep	-----------------------as required-----------------------															
	Therapy notes		x	x	x	x	x	x	x	?	x	?	?	x			
	Adverse effects			-----------------------as required----------------------------													
Custom measures and forms	Demographic information	x															
	Therapy satisfaction Likert		x	x	x	x	x	x	x	x	x	x	x	x			
	End point assessment														x		
	Qualitative pre-therapy questions		x														
	Qualitative post-therapy interview														--x--		
	Therapy delivery, fidelity and adherence		x	x	x	x	x	x	x	x	x	x	x	x			
	Attendance records		x	x	x	x	x	x	x	x	x	x	x	x			
	Recruitment log	n/a, data collected prior to recruitment															

B=baseline, P=post-intervention, FU=follow-up, ?=dependent on occurrence of session/phone-call (sessions may not occur every week)

Custom measures:

<u>The demographic information</u> collected was:

- Date of consent and baseline start date.
- Name of mental health team, name of care co-ordinator/named professional and name of referrer (if different).
- Which schizophrenia spectrum diagnosis and other diagnoses recorded.
- Height, weight, BMI, age, gender, ethnicity, and religious/spiritual orientation.
- Medication dose timing, frequency instructions, and 'date since' for current medication regimen.

<u>Satisfaction ratings</u> were collected using the following questions:

Please indicate how much you agree with each statement: (strongly disagree, disagree, neither agree nor disagree, agree, strongly agree)

- I like LDART (so far).
- I am satisfied with LDART (so far).
- I would recommend LDART to someone else with a similar problem.

<u>Post-therapy assessment questions</u> were:

- Re-rating three most important problems/issues: How much of a problem is this for you? (1-5 no problem at all—a really big problem)
- How long are you asleep per night on average?
- How long do you stay in bed after waking up?
- When do you usually 'go to bed'? (e.g., when you get in bed and turn out lights to sleep)
- How long does it take you to fall asleep? (hrs, or hrs and min)
- What time do you wake up?
- How long does it take you to feel fully awake and alert?
- How many times a night do you wake up during your sleep?
- How long are you awake each time?
- Do you ever feel tired, fatigued, or sleepy during the day?
- Do you ever fall asleep in the daytime without meaning to? (e.g., whilst doing something)
- How often do you take a nap?
- How long are your naps?
- How often in the last 3 months has your sleep been affected by the following reasons? Mind racing, don't feel tired at bedtime, psychotic symptoms (e.g., voices), frightened to sleep, get frustrated, bad dreams/nightmares, disturbed by noise, need the toilet, too hot, too cold, pain, snoring/coughing/gasping, wake up, don't know why, reason not listed here.
- Do any of these apply to you? (strongly agree, agree, somewhat, not really, not at all) I have not enough to do, I have too much to do, I don't do enough physically active things, I don't do enough mentally challenging things, I don't do enough relaxing things I don't do enough fun and enjoyable or exciting things, I don't do enough satisfying productive things, I don't spend enough time looking after myself.

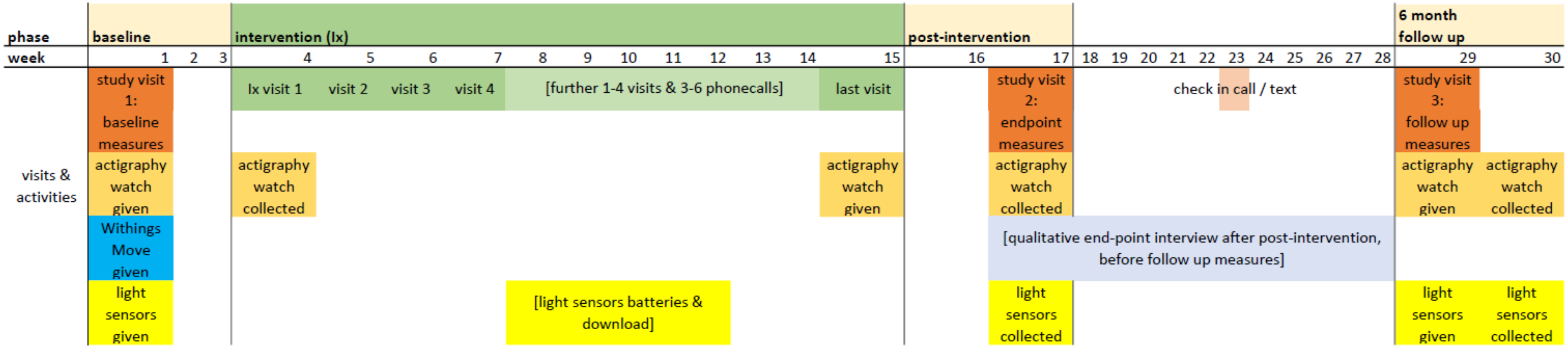

Figure S1: Research participation timeline.

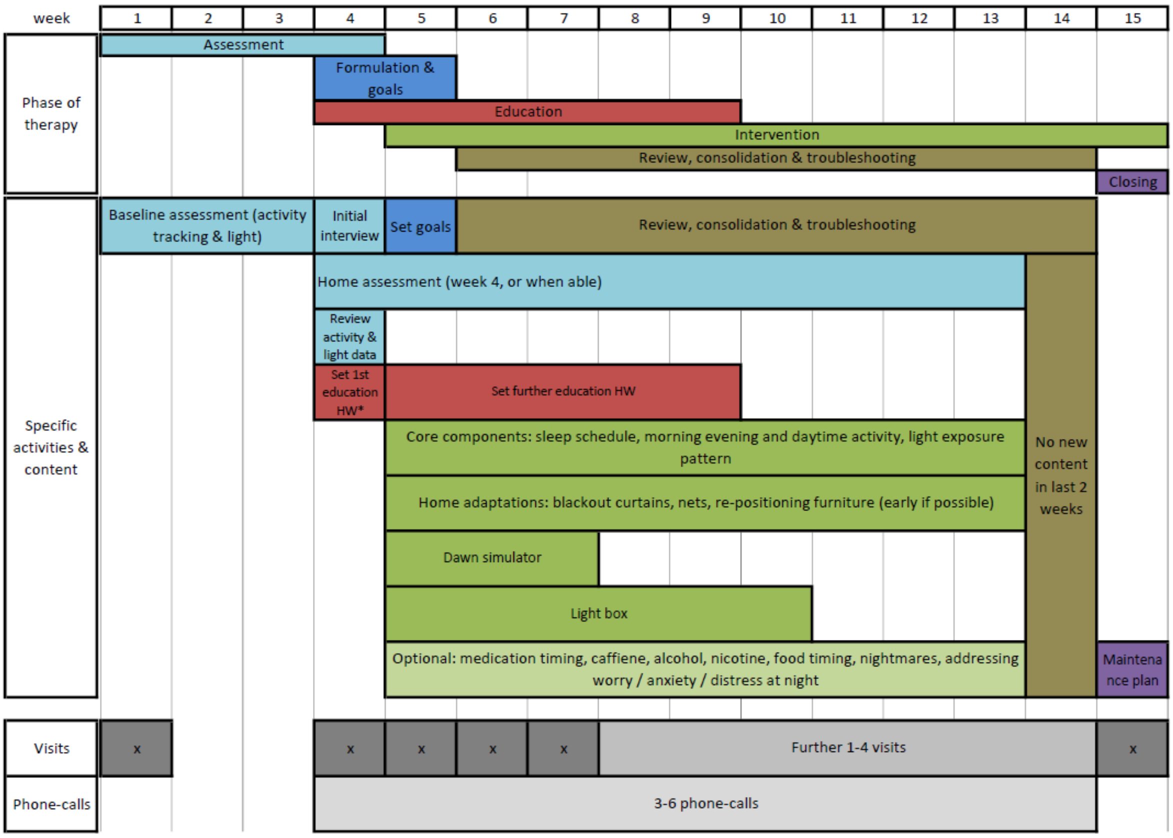

* HW = homework

Figure S2: Therapy content per week.

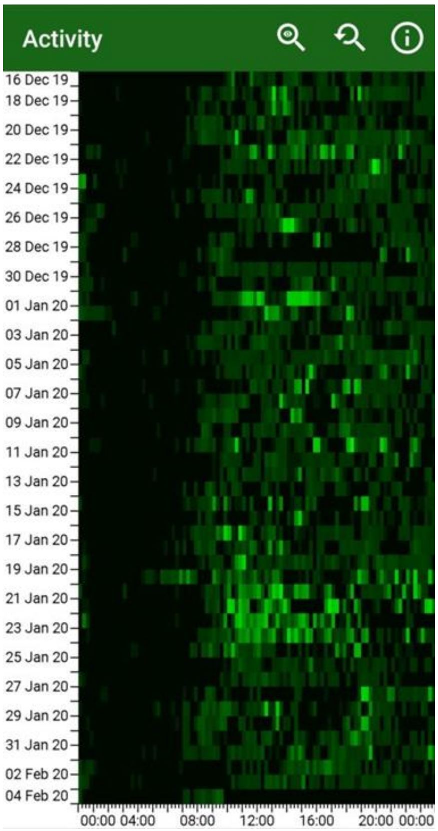

Figure S3: L-DART app, rest-activity pattern plot display.

Objective data cleaning

We collected Withings Move step counts throughout, which were used both within therapy and in our analysis. We also collected CamnTech MotionWatch 8 accelerometry and light exposure recordings (Lux) during specific periods (both wrist-worn and environmental light mounted in the home bedroom and living room). All data cleaning, analyses, and graphing were performed using R version 4.0.4 (2021) and GraphPad Prism version 9.4.1 (2022). Withings Move step counts (WS) were accessed as raw CSV files. CamnTech MotionWatch 8 data (MW) were accessed as raw MTN files, and using the MotionWare Software, they were converted to raw XLSX format. In the beginning of the data collection, start times were synchronised via the experimenter's computer system. The raw data were manually controlled for the starting date/time using the experimenter's notes. Daylight saving times were manually adjusted, and timestamps were kept as the local time of the observations. If the researcher or participant reported a device error or non-wearing period, the observations were manually removed from the raw data. WS data were collected as 1-minute epochs, while MW had 30-second epochs. MW activity count (ac) and light brightness in lux (lux) were averaged for 1-minute bins. Light exposure values were analysed after log-normalisation. Data with available information >23h in a day and valid activity in a day (any levels of activity at least 30 minutes) were kept. Using the experimenter's date notes, the data were divided into baseline (B), post-intervention (P), and follow-up (F). In the end, we had the following datasets: WS, MW-ac-wrist, MW-lx-wrist, MW-lx-bedroom, and MW-lx-living room. Daylengths, sunrise, and sunset variables at the measurement date were calculated using the R package 'suncalc' (0.5.1). For step counts or activity counts, the following variables were calculated: total, mean, maximum, interdaily stability (IS; similarity of daily patterns calculated by comparing the variance of average hourly means to the variance of all hourly means), intradaily variability (IV; fragmentation in daily patterns calculated by comparing the mean square deviation of hourly means from the previous hour to the variance of all hourly means), mean of the least active 5-hour period (L5), mean of the most active 10-hour period (M10), relative amplitude (RA; M10-L5/M10+L5), times of L5 and M10, period of the best-fit harmonic fit line that explain the observed pattern[1,2]. For light exposure measurements, the following variables were calculated: total, mean, maximum, IS, IV, L5, M10, times of L5 and M10, RA, observed period, exposure duration above 500 lx, night (0-4 am) average, and duration above 10 lx. Light exposure time-of-day distributions were calculated in 4 categories: < 10 lx, 10 – 100 lx, 100 – 500 lx, > 500 lx. For each participant, the following plots were presented: 1) Light exposure time-of-day frequency distributions for

wrist MW, bedroom wall MW, and living room wall MW; 2) Withing step count actogram (black: any step count; purple: MW activity count was used if WS was missing); 3) Light exposure rhythm for bedroom wall MW and living room wall MW (gradient from grey-darkness to red-10^6 lux; light intensity was in log-scale).

1 Gonçalves, B. S., Adamowicz, T., Louzada, F. M., Moreno, C. R. & Araujo, J. F. A fresh look at the use of nonparametric analysis in actimetry. *Sleep Med Rev* **20**, 84-91 (2015). https://doi.org:10.1016/j.smrv.2014.06.002

2 Skeldon, A. C., Dijk, D. J., Meyer, N. & Wulff, K. Extracting Circadian and Sleep Parameters from Longitudinal Data in Schizophrenia for the Design of Pragmatic Light Interventions. *Schizophr Bull* **48**, 447-456 (2022). https://doi.org:10.1093/schbul/sbab124

Additional details—findings

Table S2: Outcomes of referrals.

Outcome	Number of referrals
Recruited	10
Lost contact	4
Declined (total)	6
Declined, no further info	2
Declined due to don't want to participate in research	1
Declined due to study/therapy is too much contact/too much commitment	3
Ineligible (total)	8
Ineligible due to wrong diagnosis	5
Ineligible, due to another exclusion	2
Unable to understand study info to provide informed consent	1
Patient referred for OSA assessment, clinician withdrew referral to await result	1
Study waiting list already full at time of referral	7

Table S3: Demographics of participants.

Characteristic	n (%)	Data completeness
**Total**	10	
**Gender**		100%
Female	2 (20%)	
Male	8 (80%)	
**Diagnosis**		100%
Schizophrenia	10 (100%)	
Schizoaffective disorder, delusional disorder, schizotypal disorder	0 (0%)	100%
**Ethnicity**		100%
White British	10 (100%)	
Other	0 (0%)	
**Religious status**		100%
No formal religion	3 (30%)	
Christian (various denominations)	7 (70%)	
	mean (SD)	
**Age**	50.4 (8.59)	100%
**BMI**	30. 58 (4.43)	90% (1=weight out of date on notes, unable to weigh)
**CGI-SCH score at baseline**		100%
**Medications**		100%
Taking clozapine	4 (40%)	
Taking other oral antipsychotic	5 (50%)	
Anti-psychotic depot	4 (40%)	
Antidepressant	4 (40%)	
Hypnotic	3 (30%)	
Other medications not itemised here included those for blood pressure, thyroid, allergies, painkillers including opiates, anti-histamines, diabetes medications, reflux medications, and medications for psychotropic side effects		

Additional details on why sessions did not occur or were rescheduled

Reasons no session was scheduled were:

- participant unavailable (n=3);
- therapist unavailable (n=8);
- both available but no session required (n=1).

The reasons for cancellation, postponement, or change of format were:

- therapist rescheduled (n=2);
- participant cancelled/postponed, no reason given (n=4);
- due to family or friend commitments (n=8);
- due to other health appointments (n=4);
- cancelled/postponed, for COVID-related reasons (n=5);
- face-to-face switched to phone, for COVID-related reasons (n=10);
- due to illness (non-COVID) (n=2);
- due to poor sleep (n=2).
