# Supplementary material for: Light–Dark and Activity Rhythm Therapy (L-DART) to Improve Sleep in People with Schizophrenia Spectrum Disorders: A Single-Group Mixed Methods Study of Feasibility, Acceptability and Adherence
Source: Clocks Sleep. 2023 Dec 4;5(4):734–54. doi: 10.3390/clockssleep5040048 (PMC10742153; doi:10.3390/clockssleep5040048)
Supplement: Supplementary file 1 [file clockssleep-05-00048-s001.zip › clockssleep-2657004-supplementary-S2.pdf]

<u>Qualitative Topic Guide—Prospective acceptability</u> (pre-intervention)

At the moment, how likely do you feel it is that L-DART will work for you or someone with similar problems to you?

What makes you feel it is unlikely to work?

What makes you feel it is likely to work?

How confident do you feel about your ability to stick to the recommendations L-DART might make and to complete the ‘homework’?

Qualitative Topic Guide—Acceptability (post-intervention)

How did you feel about L-DART when you were first starting out with it?

> Was any aspect of it frustrating? Was any aspect of it satisfying?
>
> Was there any aspect you enjoyed? Was there any aspect you did not enjoy?

How well did you feel that you understood LDART and how it is supposed to work?

> Were there ever things you were being asked to do that you weren't sure how it was supposed to work / to help?
>
> What about the information in the videos? Did that make sense? Was that useful and did it add anything to have those?
>
> Did how well you understood it change as time went on? Were there any particular parts/things that made more sense or made less sense?

Were there any parts which you really agreed with / disagreed with?

[using prompts about intervention components] I understand when you are working on improving sleep, there are sometimes things you are asked to do that seem like the opposite of what you might want to do? Was that ever the case? [for instance, about waking up times, or about getting out of bed if you aren't asleep]

How well do you feel you were able to stick to the recommendations and complete the 'homework'?

What did you think about the way L-DART approached light exposure?

> What did you think about: the sun-rise alarm / light box / the graphs from the light sensors / having blackout curtains fitted / having net curtains fitted / getting more natural light / changing what light you are exposed to in the evening / avoiding screens in the evening? (delete as appropriate)

What did you think about making changes to your daytime activities and their timing?

What changes were you pleased with? Were there any activities you tried but didn't like?

What did you think about using the Withings Move and the L-DART app? Did it help?

How did you find it changing the time you go to bed and get up in the morning?

(delete) how did you find it trying to reduce or avoid daytime naps?

How did you find (delete): addressing caffeine / alcohol / medications / illicit drugs ______________ / changes to timing of sedating medications / addressing meal timing   / addressing nightmares / addressing worry / worry postponement / fear / symptoms / voices, _________   /   other

How difficult was it to participate in L-DART (seeing the therapist and completing the homework)?

In terms of practicalities? (time, amount to do, physical factors, travel)

In terms of mentally or emotionally challenging aspects?

Did it get any easier or harder as time went on?

Did you have to make any sacrifices to engage in LDART?

Was it worth the sacrifices and effort?

[RESULT / CONCLUSION]

Has your sleep pattern changed as a result of L-DART? How has it changed?

How have these changes to your sleep affected other areas of your life?

Which aspects of the changes you have made do you intend to carry on into the future?

Are there any changes you don't plan to carry on with (and why)?

How confident do you feel about maintaining changes after the end of the study?

Are there any areas that will be challenging to maintain?

How do you feel now about L-DART?

If you had to describe L-DART to someone who hadn't heard of it before, what would you say?

Was there anything you think should have been done differently or which could have improved your experience?
