# Supplementary material for: Light–Dark and Activity Rhythm Therapy (L-DART) to Improve Sleep in People with Schizophrenia Spectrum Disorders: A Single-Group Mixed Methods Study of Feasibility, Acceptability and Adherence
Source: Clocks Sleep. 2023 Dec 4;5(4):734–54. doi: 10.3390/clockssleep5040048 (PMC10742153; doi:10.3390/clockssleep5040048)
Supplement: Supplementary file 1 [file clockssleep-05-00048-s001.zip › clockssleep-2657004-supplementary-S3.pdf]

Qualitative findings regarding specific intervention components (adherence, barriers and facilitators, acceptability, views of impact):

**Intervention component**<br><br>*Background information on delivery of component for context to findings.*	**Theme/sub-theme**	**Data extracts/evidence**
**Educational content (study videos)**<br><br>*Short study videos (<5min) given to watch as homework during first one or two weeks of therapy, content then re-enforced/ reviewed verbally. Videos hosted online with links for access. Sheets for notes given. Given downloaded onto a tablet device if no internet available.*	Technical skills and confidence could be a barrier to access or delay access.<br><br>Ease of use of access method is key.	"…not confident finding the browser address bar. Assisted with accessing on laptop through instructions and encouragement" (therapy notes extract)<br><br>"[Participant] had not watched due to some difficulty getting onto the site, set-this running and [participant] watched whilst I installed curtains." (therapy notes extract)<br><br>"The one thing I did find that, you have to scroll down on the page to actually see the videos and it's, it doesn't tell you to scroll down, and I couldn't find it […] the first time I [went] looking for it" (post-therapy qualitative interview)<br><br>"…maybe cos it was technical for me […] I couldn't really understand what it was when I used to play it

Some information delivered verbally if videos not watched.		back on the laptop" (post-therapy qualitative interview)
	Supported versus unsupported access: when opportunity presented, videos were sometimes given to watch during the session	"…struggles to press play on them on the tablet lent, and struggles to select the next video to watch, I put one of the two which are most relevant for [participant] ready to play and left the tablet like this for [participant] to watch this." (therapy notes extract)<br><br>"Not watched, I suggested [participant] watch a couple of them whilst I was there, which he did, he seemed reassured that it was easy to access them and that they were short as promised." (therapy notes extract)
	Useful/informative/rings true	"…at the time a lot of it clicked, I can't remember off the top of my head, but I know that when I watched 'em it explained a lot" (post-therapy qualitative interview)<br><br>"…[participant] spent the time whilst awaiting me watching the study videos however, and demonstrated some new awareness about sleep pressure in discussion" (therapy notes extract)

		"…identified with the idea of trying to force sleep and it not helping, [participant] was not sure if it was possible not to try to force sleep if you feel desperate" (therapy notes extract)
	Participants actively engaging with the content	"[Participant] has watched and made notes on 4 of 7, plans to re-review 2, one was self-explanatory and he felt needed no notes/discussion. [Participant] demonstrated a good initial grasp of the concepts included" (therapy notes extract)<br><br>"[Participant] had accessed the videos and made some notes." (therapy notes extract)<br><br>"[Participant] had watched those I starred and made comments/notes. " (therapy notes extract)
	Complex/lots to take in, may require more than one viewing.	"[Participant] said there is a lot in the videos and [participant] might need to watch more than once to take it in, I encouraged this" (therapy notes extract)

		"…didn't make sense at first, took a few goes […] they were useful" (post-therapy qualitative interview)
	Can sometimes still make progress without	"Not watched. As [the participant] has been following all behavioural instructions without the rationale given by these videos it may not matter if [the participant] doesn't watch them" (therapy notes extract, week 7)
**Education delivered directly by the therapist**	Participants described gaining understanding of how sleep works<br><br>(see also the overall acceptability section below: "Learning was valued")	"[The therapist] explained everything that would, that the sleep project was about, very well, and she helped me understand what the processes of sleep were" (post-therapy qualitative interview)<br><br>"…explained things thoroughly, and you know and gave what her reasons were for suggesting whatever she suggested […] made common sense of what she was saying, but I've never looked at in that way, if you know what I mean" (post-therapy qualitative interview)<br><br>• Also covered in relation to specific components in explanation of rationale for each.
	When not completed:	"Provided sheets for self-reporting sleep and activity pattern for 2 or

**Self-reported activity recording**<br><br>This was suggested to all participants; it was presented as useful but not totally essential to complete.		more days as a sample (provided sheets for up to 6 days). [Participant] joked about how likely it is that [participant] will complete these." (therapy notes extract)<br><br>• In 5/10 cases there was no specific reluctance to complete these expressed, but this was not done when reviewed.<br>• The lack of a sleep diary or our rest-activity recording sheet did not appear to present a significant barrier to intervention, as a clear enough picture was given by the combination of activity tracking from the Withings watch, and self-reported usual sleep and activity routine.
	When completed therapist and service user perceived utility varied:	• In one case this was completed but didn't add much new information compared to what was already summarised during initial interview.<br>• In one case this was completed and provided an insight into night-time socialising which we then discussed usefully.<br>• In one case a participant adapted the form to record their hypnotic use and sleep for several days, without the content on daytime activity<br>• In one case a participant adapted the form to record night-time bathroom trips and caffeine consumption<br><br>"**Interviewer: Did it seem at all challenging to keep remembering to put stuff into that diary**?

		Participant: No I found it interesting. I think I was just as much interested as what [the therapist] was" (post-therapy qualitative interview)<br><br>"**Interviewer: [Completing the diary] is that something you thought was useful or less useful overall?**<br><br>Participant: No, because you had to list all the things you done in between, and I don't do much in between other than listen to the radio, watch a bit of telly. So I didn't find that helpful for myself because all's I seemed to do is woke up, had a cigarette, had a cup of tea, sat in my chair, listened to the radio a bit or watched a bit of morning telly or things, so I didn't find that really useful" (post-therapy qualitative interview)
**Interests checklist**<br><br>This was offered to all; sometimes completion was supported if not done as homework.	Could take extra prompts to complete, or require supported completion	• Sometimes took more than one prompt to complete.<br><br>"[Participant] hadn't completed the interests checklist, we completed this in session." (therapy notes extract)

Prompting to complete stopped if enough information was already collected, or if other areas were being prioritised.		• Completing together in session was also useful as it enabled discussion of interests and didn't add too much time compared to just reviewing already completed answers.
	Utility varied when completed	• Sometimes this highlighted a very limited range of interests, which was then not able to be modified much within the course of therapy.<br><br>"[Participant] identified a range of interests and has decided [they want] to get back into cooking" (therapy notes extract)<br><br>• If there has already been a lot of discussion of interests and activity routines it sometimes became less relevant to still prompt completion.
**Use of passively monitored activity pattern plots within the therapy**	Time spent troubleshooting synching issues<br><br>App use was difficult for some participants	"…but we had a problem where the graphs stopped working, it was really peculiar. But yeah she showed me the graphs and everything and talked me through it, and looked at how many steps I was doing a day." (post-therapy qualitative interview)<br><br>"Withings app:<br><br>Re-synced, this had logged [Participant] out for some reason. " (therapy notes extract)<br><br>"I tried to talk [Participant] through syncing the Withings Move in order

		that we could look at up to date rest activity patterns together but [they] found it frustrating and couldn't find the app icon so we abandoned this until I see [them] in person." (therapy notes extract)
	Activity plots were useful within therapy to compare to self-reports and discuss<br><br>Occasionally, participants reviewed these independently between sessions	"Generated rest activity plot and discussed with [Participant], very regular pattern, long sleep as described in assessment, lots of movement after 9am though [Participant] does not then wake until midday." (therapy notes extract)<br><br>"…it's really interesting looking at the graph for when you're most active, and when we started we realised I was active in my sleep as well, movement-wise, I don't have problems getting to sleep, I just have problems staying asleep…." (post-therapy qualitative interview)<br><br>"Activity pattern shows a regular bedtime, variable getting up time, visible sleep interruptions, and short bouts of walking at various times of day similar to [Participant's] s report (e.g., walk to shops or friends)." (therapy notes extract)

		"[Participant] reports being up at 10 or 10:30, but there is little activity shown before about 1pm" (therapy notes extract)<br><br>"**Interviewer: What did you think about using the Withings Move and the L-DART app? Did it help?**<br><br>Participant: I found it very informative over time […] the graphs were helpful." (post-therapy qualitative interview)<br><br>"…it depends on your behaviour. So, you know I would look at that and I would see a block or a strip […] a vertical strip on the graph of inactivity versus activity, and even I can now see the […] clearer aspects to what I was doing." (post-therapy qualitative interview)
**Altering activity routines**<br><br>This was assessed with all participants, and attempts were made to address this	Sometimes various aspects of occupational routines were already supportive of good sleep	"Discussed where within morning routine lightbox use will fit. Discussed morning routine more generally, [participant] has a well structured morning routine which suits [them]. […] [Participant] is quite happy with [their] routine, [they are] not that keen to increase

wherever it appeared to be an issue.		levels of physical activity" (therapy notes extract)<br><br>"It helps that I'm working, gives me something to do, and if they're quiet at work I'm tired, if I've struggled. But if they're busy I'm fine, I just get on with it and before I know it it's home time." (post-therapy qualitative interview)
	Some perceived a clear links between daytime activity and sleep, repeated aspects of the rationale, and were motivated to make changes.	"…keep your mind active as well, another thing she was saying, keep your mind active, and get the fresh air […]as I said it was going out and doing things and doing the, more things, going out to the shops, going outside, listening to music. […] She did say you know try and do some things, don't just sit at home and do nothing" (post-therapy qualitative interview)<br><br>"[Participant] agreed that the answer to this might be more daytime activity in the afternoons, rather than retiring to bed early. […] [Participant later started going swimming]" (therapy notes extract)
	Sleep problems were also a barrier to occupational engagement and scheduling therapy	"…it's not good being in the darkness, cos you can't do anything, you can't, can't go to the supermarket, you can't get a taxi to the supermarket, you can't get a

		bus, you can't do anything in the darkness at night." (post-therapy qualitative interview)<br><br>"[Participant] has been good at calling me back despite missing calls due to sleeping at erratic times and was keen to participate." (therapy notes extract)
	Not everyone perceived a link between activity and sleep. Some people might want an intervention to improve their sleep, but not want or be prepared to change daytime activity	"…it's like I thought it was supposed to be like my sleep but the questions she was asking like what do I do all during the daytime […] I thought it's got nothing to do with sleep […] she give me a list of courses like what you can do and stuff like cake-making or woodwork or anything and all that lot, different things but I didn't want to do it. She was like 'Why not?', and I just didn't want to do it" (post-therapy qualitative interview)
	A lack of identified interests was a barrier to progress in other areas of the therapy, such as with sleep schedule goals, stimulus control, and managing worry	"[Participant] was concerned if [they] will be able to stay awake until 6pm because [they have] nothing to do today, we discussed things [they] could do and [they] made a plan" (therapy notes extract)

		“[Participant] has continued to rest in bed for large parts of the day when [they are] at home, but if [they are] at [family member’s] house (2 or 3 days this week), then [participant] does not rest in bed and is up and about doing things.” (therapy notes extract)<br><br>“Explored other activities to do in the night if fully awake and getting frustrated in bed or awake in bed for a long time. So far [participant] just identifies TV, I agreed TV is still better than lying in bed being frustrated even though the light exposure is not ideal” (therapy notes extract)<br><br>“[Participant] may have unused time devoted to rumination and it will be useful to discuss his self-reported activity at the next session” (therapy notes extract)
	Poorly timed goal directed activity (especially online) could also be a barrier to adherence to sleep schedule plans	“(Participant] becomes immersed and then cannot stop, and becomes stressed and cannot sleep.” (therapy notes extract)

	Major changes in daytime activity only occurred in a minority of cases, but appeared impactful on quality of life and on sleep when they did	"I'm starting to go out to [watch X, sport] again, which I enjoy [watching X, sport]. I enjoyed playing [X, sport] when I was able to. […] This is the first day out I've had in I can't remember how long" (post-therapy qualitative interview)<br><br>"[Scheduled two activities to try]. [Participant] followed through with both of the plans set, and tried doing [puzzles] on more than one day when [they] had some free time in the house, [they] enjoyed it, found it interesting, and plans to continue. [Participant] also went out for a walk down the road on the one day [they were] not going into [town]. [Participant] is pleased to have been out every day since we spoke" (therapy notes extract)<br><br>"[Participant] is really pleased with [their] progress with starting going to the gym, and is feeling positive in general. [Participant's] sleep has been drastically improved since starting at the gym." (therapy notes extract)
	Not relevant for all	• One participant was already highly physically active, and one was

**Increasing physical activity levels or step count**<br><br>This was suggested to most, unless it appeared to be already 'optimised'. The level of emphasis varied depending on how significant an issue this appeared to be.		highly physically active within the constraints of their health and mobility.
	Using passive monitoring supported goal setting and progress monitoring	"Maintaining a level of at least 5K steps per day, and usually closer to 10K, more than last month, last month had some days of only 2K or 3K steps, none like that this month. Gave encouragement to continue." (therapy notes extract)<br><br>"**Interviewer: …been useful in terms of having the watch on and being able to see your sleep pattern?**<br><br>Participant: Yes, I found it pretty helpful because it monitors, it monitors my steps, it monitors my sleep, and I can look at it on the phone" (post-therapy qualitative interview)<br><br>"I mean I've never heard of anything like that before, about the graphs working out your sleep and your getting up, your walking. And it was very good to do that" (post-therapy qualitative interview)
	Adherence was influenced by social network, reasons	• Large increases in physical activity levels were created when a change in routine including others (e.g., friends or family) occurred, which were generally not sustained when others were away or unavailable.

	to go outdoors and destinations to walk to.<br><br>If unable to leave the house, it was challenging to find non-exercise physical activities to recommend.	• Some activities participants were not interested in doing alone, only if they could find a friend or family member to go with.<br>• People with dogs had more reason to go for walks with no specific destination.<br>• Homework or goals to taking a walk purely for health and sleep reasons were less adhered to than when there was a destination specified.<br>• Making an ongoing commitment to a routine such as gym sessions or a walking group was successful in some cases.<br><br>"[Participant] likes physical activities but not exercise for its own sake, practical tasks, and helping others." (therapy notes extract)<br><br>"…roughly the same, I do go out but not much like I'm not really doing much. Unless I've got an appointment to be or somewhere to be" (post-therapy qualitative interview)<br><br>"…step count remains very low […] Explored possible other means of getting physical activity if not leaving the house, [participant] was initially sceptical of using any kind of exercise video but then said I

		should send some links." (therapy notes extract)
**Alteration of meal timing**	For the participants recruited this was not a major focus.	• None reported excessive late night eating which might affect sleep<br>• None reported night eating.<br>• Some started having breakfast or a hot drink as part of their new morning routine.
**Hypnotics –**<br><br>Altering timing, frequency or dose (reducing) if relevant, or liaison with prescribers.<br><br>We did not require participants to withdraw from hypnotic use before starting therapy and did not necessarily work on eliminating hypnotic use unless this was identified during goal setting.	Although most participants were not keen on hypnotics, some may have more mixed or positive feelings toward them.	"would like to be able to fall asleep more easily 'as if with sleeping pills', but [participant] acknowledges that using sleeping pills every night is not a realistic option." (therapy notes extract)<br><br>"I can go two days, a day and a half, two days or so and not sleep […] I have sleeping tablets where I will knock myself out [to sleep if I know something's upcoming." (post-therapy qualitative interview)
	One participant withdrew from hypnotic use before starting therapy without being asked, although this was not sustained	"[Participant] has stopped using Promazine and Promethazine - actually [they] did this [themself] in the baseline period before any advice." (correspondence with prescriber, in clinical notes)
	The main intervention completed was giving counselling to reduce misuse of prescribed hypnotics	"…what I used to do with my medication, I used to judge it by having a little swig, but [the therapist] suggested that I use a teaspoon or try and get a measuring

	against prescriber instructions.<br><br>Divergence from prescriber instructions sometimes only came to light during later sessions when discussing hypnotic use at more length.	spoon off the chemist or from somewhere." (post-therapy qualitative interview)<br><br>"[Participant] is often taking more than prescribed including sometimes both zopiclone and promethazine within one night […in a later session…] reports not using above recommended dose since our discussion last week as 'he knows I will report this, which is fair'" (therapy notes extract)
**Prescribed medications** – Intervention if needed around timing of prescribed medications other than hypnotics	Attempted to address divergence from prescriber instructions but without success.<br><br>There were no other cases where modifications were needed to timing of medications to support sleep timing changes.	"[The therapist] did suggest about my medication, taking my morning medication and the night-time medication both separate but I can't do that in case I forget. So what I do is I take all my medication in the morning." (post-therapy qualitative interview)<br><br>"…due to [participant's] sleep timing having been erratic and the concept of 'morning' and 'evening' is confusing as is it before sleep or in the actual evening." (therapy notes extract)
	Hypersalivation	• This was an issue for two participants, purchasing absorbent

		pillow protecting pads was found beneficial.
**Smoking –**<br><br>Reducing or altering timing. This was only attempted when participants expressed interest and readiness, or if it appeared to be a significant sleep disturber (e.g., heavy smoking during the night)	Delivering advice about nicotine being a stimulant appeared to effectively motivate reduced smoking in the night, and was remembered.<br><br>Sometimes people may become more interested in quitting or reducing smoking when they realise it could improve their sleep, as they may not have been aware of this before.	“And you now my usual pattern of behaviour was to have a brew, have a cigarette then go back to bed and try again. So you know but we changed that cos obviously you know tobacco is a, is a… trying to think what the word is, yeah it keeps you awake” (post-therapy qualitative interview)<br><br>“[Participant] agreed to move the cigarettes and ash tray out of the bedroom into the living room whilst I was there, and did this.” (therapy notes extract)<br><br>“Participant: She said about not smoking in bed when I woke up. I was smoking more cigs in the middle of the night than I was during the day. I’ve stopped smoking cigs in the night-time now<br><br>**Interviewer: And what do you think about that… does that seem ok for you or was it a bit difficult?**<br><br>Participant: No it’s better, I did it within a week, stopped it within a week

		**Interviewer: Brilliant, that's impressive… It sounds from the way you're talking about it that seems like a positive to you, is that right, to have changed?**<br><br>Participant: Yeah yeah<br><br>**Interviewer: Ok… And can you remember what [the therapist] would've said about why, why change the time of smoking a cigarette**<br><br>Participant: Cos of the nicotine or something can keep you awake" (post-therapy qualitative interview)<br><br>"[Participant] mentions [they are] taking a renewed interest in quitting or seriously reducing smoking, I encouraged and explained how nicotine affects sleep (adversely). […at a later week…] has reduced [their] smoking from 30g per 2 days to 30g per week, I congratulated [them] and encouraged [them] to continue reducing" (therapy notes extract)
**Caffeine** – reducing or altering timing	One participant reduced caffeine before starting	"[Participant] was consuming a very high amount of caffeine per day, over 1000mg once added up on some days, included 10-20 coffees.

	therapy, without being asked, this was sustained	[They had] actually already reduced this before we spoke about it today" (therapy notes extract)
	Calculating and communicating average daily caffeine consumption in mg and comparing to daily recommended levels was surprising to many participants and often resulted in motivation to change behaviour	"Yeah, she said you should have between three and three hundred and fifty, and I was topping a thousand a day, and now I've got it down to two hundred, maybe two hundred and fifty, and that has helped massively, everything, almost everything I have hot or cold is decaf now […]the caffeine diary, I was *shocked* how much caffeine I have in a day." (post-therapy qualitative interview)
	Homework to try decaf versions completed	"Caffeine recorded daily and slightly reduced, [Participant] has not purchased decaf yet but plans to soon. […at a later week…] [Participant] has now purchased decaf and plans to switch to this during the afternoon each day and report back […at a later week…] [Participant] has got decaf Kenco, tried it and likes it, uses the caffeinated coffee 'as needed', I suggested keeping this to the earlier part of the day (e.g., till midday if possible), and described typical caffeine half life." (therapy notes extract)

<table>
<tr><td>Addressing comorbidities

Using worry postponement to address worry or psychosis-related distress

Problem solving sleep issues related to physical health issues, sleep apnoea or parasomnias</td><td colspan="2">“Revisited conversation about making a note of what you are working on in order to return to it later and be able to leave it alone in order to rest or sleep.” (therapy notes extract)

“…when I’m trying to relax and calm the voices down I’ll listen to the radio, and I’ll sing along in my head sometimes and that helps, that’s a really good sleeping aid, listening to a very quiet radio.” (post-therapy qualitative interview)

“I just don’t have the ability to switch off. I really find it hard to really calm down and stop being restless and fidgety.” (post-therapy qualitative interview)

“I don’t go out as much during the night or early evening, I don’t like going out when it’s dark, but.. it just, it’s, as it says, ‘Seasonal Affective Disorder’, and it does make me depressed sometimes, when it goes dark early, I’ll just, I’ll be stuck in all day or I’ve not been up to much and.. It’s the isolation that gets to you.” (post-therapy qualitative interview)

• There was a moderate amount of time spent during therapy discussing and trouble-shooting impact on sleep from a range of physical health conditions, this is not described using specific examples here due to potential identifiability.</td></tr>
<tr><td>Sleep compression and reducing the time in bed window</td><td>Using sleep compression to recruit sleep pressure in people with insomnia-type</td><td>“me and [the therapist] talked and she says ‘Start going to bed a bit later, maybe ten o’clock, and start getting up earlier even if I haven’t</td></tr>
</table>

Gradually reducing the time in bed window and reducing or eliminating naps.	problems was adhered to and positively evaluated	got work’. So that’s what I’ve done, and I feel like it’s working because I go to bed about ten half-ten, something like that now, and I’m tired when I got to bed, I’m really tired. I’ll get in bed, doesn’t take long to get off” (post-therapy qualitative interview)<br><br>“Compressing my sleep pattern, so going to bed later but getting up earlier and changing what I do during the night if I am awake, so, yeah, you know that was what I found really helpful.” (post-therapy qualitative interview)
	Reducing excessive time in bed window or excessive sleep duration was mostly successful	“…this week [Participant] has got up at 9am each day despite it being difficult and [they have] found [they have] felt actually more awake in the day not less. I encouraged that this is good and what we would expect to happen. [Participant] had excessive length of time in bed before (13hrs).” (therapy notes extract)<br><br>“Yeah, we worked on a schedule of lowering the length of time that I was in bed, cos I was resting a lot.

		[…] So we started off at about eight hours, and then we got it down to six hours. […] I did struggle keeping to the times of falling and going to sleep at twelve and getting up at six, sometimes I stayed in bed for an extra hour or something like that. But I did my best and it did improve my sleep overall, I managed to get- I wasn't getting any sleep at first and I managed to get about two and a half hours, three hours sleep a night. So for me it was a success" (therapy notes extract)<br><br>"About two or three weeks ago I was getting all that right, and then all of a sudden insomnia just kicked in, I've been awake a long time, I've been asleep a long time, I'm awake a long time and then asleep a long time. But [the therapist] suggested that the ideal pattern to get into is to have seven or eight hours of sleep, maybe eight and a half at a push, is adequate sleep to get you through the next day". (therapy notes extract)
	Reducing or eliminating napping when relevant was generally successful	"…fallen asleep by accident on a few occassions if he is not out, and if he is reclining listening to music.

		[…] fallen asleep unintentionally around midday for about an hour a few times but most days is not napping". (therapy notes extract)<br><br>"….reports success avoiding daytime naps". (therapy notes extract)<br><br>"I don't like falling asleep after I've woken up because it makes, I think it makes me more tired." (post-therapy qualitative interview)<br><br>"**Interviewer: …any changes that you've made with [the therapist] that you think are worth keeping up for the future?**<br><br>Participant: I'll probably try not to nap in the day.<br><br>**Interviewer: Not napping, yeah, ok. And in your own words [name], what difference do you think that makes to not have that nap in the daytime?**

		Participant: Otherwise I won't feel tired at nighttime." (post-therapy qualitative interview)
**Stimulus control** ('go to bed only when sleepy', 'bed only for sleep' and 'the 15 minute rule')	Sometimes there was resistance to these suggestion, but not often	"Although it takes [Participant] a long time to fall asleep he is not keen to only go to bed when sleepy, he describes being quite relaxed in bed, and nothing like the description of sleep effort." (therapy notes extract)
	Going to bed only when sleepy was harder for those with limited activities and interests, than those who went to bed early due to excessive sleep concern.	(see above, in section "Altering activity routines')
	Participants bought into the rationale for the 15 minutes rule and tried this thoroughly often with good results	"…you know when I wake up, to not just lie in bed and try to ger back to sleep, to get up and then re-approach the sleep again after a while you know […]<br><br>**Interviewer: How did you find doing that, was that something that came naturally or did it take much practice to put that into play?**<br><br>Participant: Yeah, yeah, yeah, I had to practice to do what I was, what we'd worked on, yeah." (post-intervention qualitative interview)

		“Participant reports doing this all week, he watched TV, did not try putting the radio on, he found that once he was up watching TV he did not feel sleepy and was then awake until morning, but he did find that he got longer time asleep this week before first waking up”. (therapy notes extract)<br><br>“You are already doing the right thing if you cant sleep in the night you get up rather than lying in bed getting frustrated.” (from post-therapy maintenance plan)<br><br>“You have removed the TV from the bedroom! This is great and will be helping your sleep a lot. If you keep other activities like reading and using the i-pad out of the bedroom, or at least out of the bed, this will also help strengthen the link your brain makes between bed and sleep.” (from post-therapy maintenance plan)

	Using the bed (sleeping surface) only for sleep is difficult if participants do not sleep in their bed	• In three cases the participant did not sleep in their bed, either mostly or entirely sleeping in the livingroom on the sofa. Thus, many activities were completed in the sleeping space.<br>• One did not have a suitable bed during the therapy window and there was no financial resource to address this.<br>• Two attempted to start sleeping in the bedroom. Despite trials on several nights both reverted to sleeping in the living room by the end of therapy.
**Regular rise time**<br><br>Advised for all, more of a priority where rise time was more irregular at baseline.<br><br>During intervention this can be looked at in relation to adherence to recommendations. Whilst developing the ability and pattern to sustain a regular rise time later during follow-up may more accurately considered an outcome.	This was usually accepted as a suggestion and attempted to some degree, but with varied levels of success.<br><br>With those two participants with irregular or non-24 sleep-wake timing, we were not successful in establishing a regular rise time for a sustained period.	"Before my suggesting it [participant was already keen to have the same wake time through week and weekend." (therapy notes extract)<br><br>"I couldn't regularise myself […] I mean I just flop, I just crash when I'm ready, or if I've taken a sleeper." (post-therapy qualitative interview)<br><br>"Yeah well I'll keep on getting up earlyish all the time, because I've got used to it now and the, my bodyclock's working now" (post-therapy qualitative interview)<br><br>**"Interviewer: Do you think some of the changes you've started with**

		**her, do you think you'll keep them going, and maybe do a bit more of something?**<br><br>Participant: I think I'll look at spending less time in bed and more time being up, like because I was just lying there being restless. So I'll take influence from that, and going to bed at certain times and getting up at certain times, having more of a routine. So that's it." (post-therapy qualitative interview)<br><br>• See also rest-activity pattern plots for change in regularity of rise times
**Morning routine**<br><br>This was addressed at the same time as regular rise time and setting the dawn simulator up. Less so if the existing routine was already helpful.	Participants often tried out changes, some small changes were sustained and became habits	"activity pattern together it shows little activity during the morning […] discussed […a later week…] [Participant] now has a drink [of tea] in the morning and watches news, compared to previously doing nothing at all until much later, [they are] pleased so far with this change and happy to try other changes" (therapy notes extract)<br><br>"[Participant] has been opening the blinds and going outdoors to shops or friends in the morning. […a later week…] [they] stopped going

		outside each morning." (therapy notes extract)
**Altering overall sleep timing** (bed time and rise time)	Collaborative process of negotiating target schedule	"Discussed desired rise time: [Participant] had been keen to aim for waking at 7 or 8, however I discussed with him that as he as always been a late type before his current non24hr pattern, that it might be more realistic to have a regular rise time of 9 or 10. Would this be more desirable to him than his non24hr pattern? He said yes late was preferable to sometimes nocturnal" (therapy notes extract)<br><br>"[Participant] sleeps at unconventional times preferring [this] […a later week…] After some discussion [participant] and I have settled on aiming for 2-3am bedtime, and wake time of 11am." (therapy notes extract)
	The process of therapy was necessarily quite different for non24 pattern than those with an entrained rhythm at baseline. Optimal timing of sessions was not the same as when delivering a more insomnia focused approach.	"I have instructed [participant] to continue to allow his sleep and wake to advance by 2hrs per day, writing down approximate times predicted for sleep and wake between now and then, and scheduled our next contact for when

	Although we were able to hold a diurnal pattern briefly on two attempts, this was not sustained.	it will naturally arrive at a diurnal pattern.” (therapy notes extract)<br><br>“I’ve overslept one day and then it’s, it’s set me pattern off, I’ve overslept by about three or four hours and then of course you’re awake longer then. And then when it comes time to go to bed you’re asleep again for a long time […] I’ll look again at managing that myself. I have noticed a change in my sleep pattern over the past six months […]. The only problem is when I over-sleep when I don’t get out of bed after 8 hours, I find that I’m groggy and that and I can’t get up and I’m in bed for ten twelve hours” (post-therapy qualitative interview)
	(see below section ‘Even when sleep pattern had not changed significantly, improved understanding was valued)	(see below)
**Dawn simulator/wake up light**	Overwhelmingly positive experiences with dawn simulator	“Yes I found it quite helpful because the light comes on, the birds are tweeting, and it makes you feel fresh when you wake up. You can come round with that light, and

		I feel good and ready to face the day." (post-therapy qualitative interview)<br><br>"…pleasant […] Better than a screeching you know or a klaxon or something." (post-therapy qualitative interview)<br><br>"And the light, the alarm wasn't that loud so it didn't really wake me up you know that suddenly, it sort of like brought me to but gradually." (post-therapy qualitative interview)<br><br>"So she gave me one of them which […] triggered me to get up and do what has been suggested." (post-therapy qualitative interview)<br><br>"…it slightly lightens up the room, which will wake me up, and it comes on at eight, and it always wakes me up. And then the alarm the birds tweeting that goes off at half-eight […] instead of snoozing like I used to do I get up." (post-therapy qualitative interview)

	Some may not have used an alarm clock much/at all before	"I've never tried to use an alarm to tell you the truth. I just wake up when I wake up and go to sleep when I go to sleep." (post-therapy qualitative interview)
	Brightness was adjustable, occasionally this may be needed	[set to] about 2/3 brightness […] Full brightness seems dazzling to [participant]." (therapy notes extract)<br><br>• All other participants used on full brightness
**Increasing daytime light exposure**<br><br>By going outside more and opening the curtains	Going outside more was a difficult behaviour to change	"I was asked to go outside prior to the light being brought, and I said 'I just can't do that', you know on a regular basis, I just can't roll, and you know go out and spend half an hour walking or something like that, just not gonna happen. So the light [box] was brought in." (post-therapy qualitative interview)<br><br>"Get light in the daytime - You go out a fair amount some days but not others. It will help your sleep more if you can get outside every day and especially in the morning." (from post-therapy maintenance plan)
	The rationale was often bought into, which only sometimes led to change	"Well I think it was going outside was more important [than using a light box]"

		"It's very important you see she said for going out in the light, it's very good, you know, you've got to out and get fresh air and get the light." (post-therapy qualitative interview)<br><br>"I was sat in the garden the other week when it was nice. So at least, I'm thinking well at least I'm getting the fresh air and the light. I sat in the garden for an hour, just to get some more daylight outside while I'm out." (post-therapy qualitative interview)
	Opening the curtains was an easier behaviour to change once any privacy issues were addressed with net curtains if needed	"So it was, we was working like literally of, working with natural light, just in terms of, just getting like more natural daylight cos I was always having the curtains closed as well. So I was opening the curtains." (post-therapy qualitative interview)<br><br>**"Interviewer: …if you had the blinds closed before maybe perhaps for privacy or just more comfortable?**<br><br>Participant: Yeah it's just something, it isn't for any reason I just didn't think to open 'em, I just

		used to switch my light on. But when [the therapist] said to start opening your curtains […] I just open my blinds […] in the morning." (post-therapy qualitative interview)
**Light box**	Participants were less motivated to use the light box if they could not perceive any immediate effect	"I just used it for half-hour intervals, like [the therapist] told me to do, because I wasn't getting out much, so I was using it to just get some sunlight. I didn't really feel that it made a difference, I didn't notice a difference to myself being more awake, because I'd started getting sleep so that made me feel like that was the reason that I was more awake." (post-therapy qualitative interview)<br><br>**"Interviewer: …whether that felt like it was making you more awake?**<br><br>Participant: […] I'm not sure I'm that sensitive […] I just use it, and expect my circadian rhythm to kick in, with the light effect on my whatever it is […] in the brain, and help regularise you know, I have no particular sensation [of it having any effect] […] and I can't say I had,

		no.” (post-therapy qualitative interview)
	In one case the participant felt more awake after use and was very positive	“…one of the biggest aids there is to help is that light box. It’s amazing […] I wish I’d got one sooner.” (post-therapy qualitative interview)
	Participants sometimes declined before trying it, if it wasn’t the highest priority this was not pushed	“Discussed light box and dawn simulator alarm:<br><br>[Participant] declined both for now but was open to revisit. ” (therapy notes extract)<br><br>“Offered light box to use in morning to supplement daytime light, [participant] declined. ” (therapy notes extract)<br><br>“[Participant] often gets light very soon after waking, outdoors, I advised to continue or increase how often [they are] outdoors soon after waking. Offered light box if desired but [participant] feels [they go] out so often it is unnecessary which seems fair.” (therapy notes extract)
	In one case it was accepted but not tried sufficiently	“…to be quite honest with you I’ve only used it about, must be about a dozen times.” (post-therapy qualitative interview)

	In one case it was tried and not tolerated	"…it was too strong for me, the light, it didn't help me, it made me feel dizzy. So she said 'I won't use that then'. I was gonna use it but as I said I gave it a try but it didn't work for me that." (post-therapy qualitative interview)
**Reducing light at night**	It was necessary to advise participants to turn the light off or shut the existing curtains at night	**"Interviewer: [Are there any other] process[es] that might still be ongoing and that you can still kind of benefit from?**<br><br>Participant: I started shutting the blinds [at night in the bedroom].<br><br>**Interviewer: […] did that seem to make sense?**<br><br>Participant: Yes, yeah, it did yeah." (post-therapy qualitative interview)<br><br>"[Participant] has continued to have the side lamp on, [participant] feels it doesn't stop [them] sleeping. Discussed rationale for having this off, or switching to the dim red light on the dawn simulator, played [Participant] the videos on timing of light and strength of rhythm, discussed that the negative effect of continuous light at night may not be immediate but over a longer period will impact circadian rhythm.

		[Participant] agreed to switch to using the dim red light. […at a later week…] [Participant] has now begun turning off the side light at night. [Participant] continues to turn off the side light at night, and use a torch to get into bed safely (side light not reachable from bed).” (therapy notes extract)
	Blackout curtains when relevant were readily accepted and positively evaluated	“Bedroom curtain allows a lot of light in during the early morning which wakes [the participant] up at 4 or 5am, or makes it more difficult to get back to sleep. [Participant] accepted blackout fabric and fitting offered.” (therapy notes extract)<br><br>“…so the light streams in in the morning. But I did find that I was waking up before the set time […] you know I get very little sleep generally, it’s erratic, sometimes long sometimes short. Yeah. But they serve their function, I appreciate those [blackout] curtains.” (post-therapy qualitative interview)<br><br>“**Interviewer: [had some blackout curtains fitted] and what did you think of that as an idea, did that seem like something that might be useful?**<br>Participant: Yeah miles better it was, a hundred per cent better<br>**Interviewer: […] Do you mean by that that you are getting better sleep because of that, have you noticed an effect, on your sleep?**<br>Participant: Yeah […] I was getting

		up later, instead of like five o'clock four o'clock when it became light." (post-therapy qualitative interview)
	Having a not-fully dark dim light option to offer for at night was occasionally very important	"[The therapist] suggested I use the dark light instead of the lamp that I have in my bedroom, because it's not as bright as a lamp […] And I found that a little bit helpful, rather than sleeping in the dark, because […] I get nightmares when I sleep in the dark. So the nightlight has been pretty useful." (post-therapy qualitative interview)

Barriers and facilitators to adherence for the intervention as a whole:

Theme/sub-theme	Data extracts/evidence
**Expectations**<br><br>Expectations of success were sometimes low due to duration or severity of problem	"Participant: I've been through a lot of stuff, you know trying different things and it really hasn't made any difference, so yeah, you know I'm not overly confident […]<br>**Interviewer: And what makes you feel it would be likely to work?**<br>Participant: Nothing [laughing]. Sorry." (post-therapy qualitative interview)
Participants were motivated despite this	**"Interviewer: Can you remember how you would've felt getting started with L-DART, perhaps the first time you met with [the therapist] or anything like that?**<br>Participant: Well I was thinking it probably wouldn't work for some reason, I was thinking 'I bet it doesn't work' […] it's been twenty years this has been going on, so why haven't people helped me before." (post-therapy qualitative interview)

	"I'm just not hopeful in general because of how much I struggle with my sleep. That's really what it is." (pre-therapy expectations mini-interview)<br><br>**"Interviewer: But you sound quite committed to trying to change it?**<br>Participant: Oh yeah I'm gonna try and change it." (pre-therapy expectations interview)
**Confidence to adhere varied**<br><br>Some admitted expecting to have difficulty adhering	"[Participant] says [they] definitely wants to give it a go although [they] can't promise [they] will take everything on board straight away, [participant] also worries nothing can possibly help [their] sleep." (therapy notes extract)<br><br>**"Interviewer: What makes me feel it isn't likely to?**<br>Participant: Knowing my own habits so far, for the past especially twelve eleven years here in [area], yeah." (pre-therapy expectations mini-interview)<br><br>**"Interviewer: …how confident do you feel about your ability to stick to the recommendations that L-DART might make, and complete the homework?**<br>Participant: Yeah, yeah, quite confident with that, yeah I'll definitely do it yeah, yeah." (pre-therapy expectations mini-interview)
**Participants were often keen**	Participants were often keen and started taking steps during the baseline period despite receiving no advice yet, including:<br><br>• Reducing caffeine (this was sustained)<br>• Stopping using hypnotics (this was not sustained)<br>• Starting tracking daily steps using the Withings app before being advised to (L-DART app not installed until week 4, initial assessment) and comparing to subsequent sleep
**Participants will sometimes try off-protocol things**	"[Participant] had made wake up time much earlier before being advised to do so and had set his alarm for 5:30, bed at 11pm, (sleep window 6.5hrs)." (therapy notes extract)<br><br>"One day this week [participant] had an early start, [they] used lavender on [their] pillow the night before, and fell asleep without a problem. This is not part of L-

	DART but I encouraged [participant] that as it seems to work for [them] to use this when [they feel] it is relevant." (therapy notes extract)
**Format—in-person vs. over the phone**<br><br>Either was fine in many instances<br><br>In person preferred by some generally<br><br>In person preferred by some for understanding intervention rationales<br><br>In person preferred for explaining technology	"…she'd be on the phone quite regularly, you know just to see how I was going on and what, if there was anything she could tweak." (post-therapy qualitative interview)<br><br>"…because I couldn't make it on time for the meeting so she said 'can I phone you later, can we do it over the phone later?' so she done that […] I prefer to do it face-to-face. I tend to take it in a bit more. If it's a phone call my mind can go with the fairies a little bit and I drift off thinking about other stuff. But if it's a one-to-one like me and you talking you've got my attention." (post-therapy qualitative interview)<br><br>**"Interviewer: Do you prefer to meet in-person, or would you be happy if it was conducted on the phone, which do you think is better for you?**<br><br>Participant: I'm not sure to be honest, they're both as good as each other." (post-therapy qualitative interview)<br><br>**"Interviewer: [if the therapist sent] out a watch or an app to you to use [then gave] advice over the phone, or do you think it was easier if she was there to go through those with you?**

	Participant: I find it easier when someone shows me how to do something.” (post-therapy qualitative interview)<br><br>“When she came she used to play it [step-tracking data] back and you could see it all green coming out which was, you know, where you’ve been sleeping and where you’ve been waking up. Couldn’t imagine doing that on the phone.” (post-therapy qualitative interview)<br><br>“…to get an explanation over the phone of something is harder than being in-person. If somebody can show you something that they can talk you through, so yeah it’s better in-person really. I feel that if it’d have been all over the phone it wouldn’t have been as interesting or, or ability to understand really, yeah.” (post-therapy qualitative interview)
**Scheduling**<br><br>Therapist was usually able to be flexible with session timing which was appreciated<br><br>Reminders were offered and sometimes used, reducing missed appointments	“…always gave me plenty of notice and all that so, you know and I’d make sure I was available when, when she was either ringing or calling you know.” (post-therapy qualitative interview)<br><br>**“Interviewer: [Anything] to do differently, so coming at a different time of day or anything like that?**<br><br>Participant: No because she used to arrange with me what times it was, I give her the times what, you know

	when she was, she came then." (post-therapy qualitative interview)<br><br>"…she's worked around me rather than me working around her." (post-therapy qualitative interview)<br><br>"Participant doesn't use a diary so suggested I contact him nearer the time with day and time." (therapy notes extract)<br><br>"…texted ahead of planned time, and [participant] replied requesting to reschedule to tomorrow. I replied offering time options." (therapy notes extract)
**Duration/intensity of intervention**	"**Interviewer: how long the sessions went on with her for, any communication between sessions like texts or calls, how do you feel that went for you?**<br><br>Participant: Alright. OK." (post-therapy qualitative interview)<br><br>"…we always had something to talk about if you know what I mean, you know so yeah I don't think it needed any extra visits, we covered the ground that needed covering you know in the visits that were made." (post-therapy qualitative interview)<br><br>"Once a week was alright, visiting, there wasn't any need to visit more or have a conversation on the phone with [the therapist]. She explained everything and wrote things down, so I just used to do my best and try and do the things what she's wrote down to do." (post-therapy qualitative interview)
**Interpersonal factors -** Meeting and speaking to the therapist before the initial	"I was nervous about it, because I'm not very good meeting new people […] Yeah as I got used to [the therapist] and [the therapist] got used to me I could open up more definitely." (post-therapy qualitative interview)

interview may have helped with rapport for those who find new people difficult	
**Interpersonal factors -** Questions on daytime activity could seem invasive if the reason for asking wasn't properly conveyed or understood	"I just kind of felt the questions was a bit, like I say personal / [Yeah] /. That was all, cos it's like I thought it was supposed to be like my sleep but the questions she was asking like what do I do all during the daytime." (post-therapy qualitative interview)
**Interpersonal factors -** Participants spoke positively of the therapist, whenever possible reasons for positive views were given this was most often: explaining things well, being clear, and making things understandable, and sometimes: listening, and feeling enough time was given.	"**Interviewer: [how] did a first meeting go when she explained things to you?**<br><br>Participant: Yeah she was dead nice and she explained what was gonna go on and stuff.<br><br>"Yeah, she was fine, yeah she was very welcoming. She talked me through everything that was gonna happen and it was about a sixteen week project I think it was about. Yeah she was fine yeah, she was great." (post-therapy qualitative interview)<br><br>"…the amount of time taken to share my answers to your questions […] I feel more involved, yeah. And I feel that I'm being listened to more." (post-therapy qualitative interview)
**Social environment**<br><br>Impact can be positive or negative<br><br>Having commitments can improve routine	"I have commitments as well, you know that I've got to keep going for." (post-therapy qualitative interview)<br><br>"… friend got Covid so I couldn't go up [to visit] so I didn't do as many like in the last week, as many steps." (post-therapy qualitative interview)

Lack of people to plan an activity with limits interest sometimes	"I asked if he might go [to do this exercise activity] other times even when [their young family member] is not staying but [participant] feels [they] would not enjoy it if not taking [their young family member]." (therapy notes extract)<br><br>"I noticed that [participant] socialises in the middle of the night not infrequently and will discuss with [them] how to manage any pressure [they] might feel to be awake at times other than [their] preference." (therapy notes extract)<br><br>"…worse sleep during the last week due to family-related stressors." (therapy notes extract)<br><br>"Visit as arranged by phone, rearranged from yesterday by [participant] as [their young family member] would have been present yesterday." (therapy notes extract)
**Physical environment**<br><br>Noise, physical comfort, pets, and staying at other's houses, may all impact on intervention adherence and effectiveness	"[Participant] reports still waking up intermittently from 12 until 2 or 3 when the traffic goes quieter." (therapy notes extract)<br><br>"…sleeps in bed with [their pet]." (therapy notes extract)<br><br>"[Participant] sleeps in the same place [they spend] most of [their] waking time on the sofa. This is not long

	enough for [them] to stretch out to [their] full length so [their] legs are on the sofa arm." (therapy notes extract)<br><br>"[Participant] is away all of next week staying with family, so it will not be practical for [them] to follow these instructions then." (therapy notes extract)<br><br>"[Participant] accepted a second light box (the study had spare) to keep at [their family member]'s house to reduce having to transport it back and forth." (therapy notes extract)
**Impact of COVID**	• Participants reported having no issues with COVID measures adhered to during visits, including mask wearing, and some visits being switched to phone due to COVID infection or COVID exposure.<br>• Some therapy time was lost due to COVID (see cancelled/postponed visits reasons), and some progress with goals was delayed or hampered due to missed time due to COVID or post-COVID fatigue.<br>• There were limited measures in place restricting activities during the study, and this was not cited as a reason for not pursuing any social or daytime activity-related goals by these participants. If we had more participants who were extremely clinically vulnerable to COVID or have vulnerable household members this could have been an issue.
**Linking with other care providers and services**<br><br>Time spent seeking information or sending referrals to local sleep services<br><br>Time spent liaising with mental health teams	"…referred to a sleep clinic in the recent past, but they could not see [them] due to covid, from the notes it seems like this was for insomnia, however I have made contact to check if there was any other sleep disorder suspected." (therapy notes extract)<br><br>"My phonecall was returned by [staff member] from the sleep service, she thought that the bouts of movement

	could be caused by re-occurrence of apnoeas, which [participant's] machine shows are happening." (therapy notes extract)<br><br>"[Participant] has not had contact from anyone for a bit. I'm not sure if [they have] a key worker/CC allocated. If there is can I find out who it is please? Maybe we could liaise?" (email sent to care team, from therapy notes)<br><br>"I suggested [participant] took [their] night medications at night, [participant] did, and now complains of morning hangover/sedation. For now I have suggested [they] try taking it earlier in the evening. I wanted to ask if you had any other advice for me to pass on, and wondered if it would be useful to liaise? I wasn't sure if the [specific medication] was targeting depression/anxiety, or sleep, or both." (email to psychiatry consultant, from therapy notes)

Acceptability of intervention as a whole

**Theme/sub-theme**	**Data extracts/evidence**
Participants suggesting others should do L-DART	**"Interviewer: If you had to describe the L-DART study to someone who hadn't heard of it before, what do you think you'd say to describe it**<br>Participant: Just have a go at doing it, if your sleep's bad have a go at doing it, it's alright." (post-therapy qualitative interview)<br><br>"Participant: …Well worth taking part in. You know, give it a good go, that's what I'd suggest." (post-therapy qualitative interview)

	"Participant: I would advise people to go on this sleep therapy-<br>**Interviewer: You would**<br>Participant: -advise people yeah, cos it's worked for me hasn't it so that's a good thing." (post-therapy qualitative interview)
Positive overall comments	**"Interviewer: …could've been done anyway differently or which could've improved your experience of the L-DART project in any way?**<br>Participant: No, no, it was, it was, the way it was structured was good, well it was good for me." (post-therapy qualitative interview)<br><br>**"Interviewer: was there anything on that practical side** [the therapist] **might have suggested that you may have tried or thought about but it didn't really work or wasn't something that you liked?**<br>Participant: No I can't really think there was anything what wasn't helpful. I think it was, all the ideas were very good what [the therapist] was saying. I can't, I can't remember anything negative about anything." (post-therapy qualitative interview)
Difficult at first, takes some commitment	"It's something I strongly recommend to anybody who was, who had the right frame of mind to do it. If you'd offered me this twenty years ago I just wouldn't have took it, cos I wasn't in that frame of mind to think something like this would get, help me improve and get better sleep. You've got to want to change before you can change." (post-therapy qualitative interview)<br><br>"I would've thought no there's no way that I'll be able to do that, I'll just have to cope with [the problem]. But then as I said [the therapist] gave me the different things to do, getting up earlier, which was a bit difficult at first, but it wasn't too bad because it was in stages. It wasn't a big step from the beginning right to the next week, it took a few weeks for it to eventually go to the ideal time that I want to get up early." (post-therapy qualitative interview)<br><br>"I was naturally against like, sort of working against her in a, not in a.. wrongful sort of way, I just naturally didn't want to entertain that sort of thing because I saw it as stress. But I did do it, and I did adjust to altering the times I go to sleep and getting up earlier and things and using the Withings watch […] as weeks went on keeping to the time schedule got easier, I found it less distressing. Because at first I found it distressing, like going to bed at a certain time and getting up at a certain time, because I

	was used to just lying there resting, and rather than sleeping." (post-therapy qualitative interview)
Learning was valued for enabling better self-management	"'don't go to bed too early', […] she said 'no don't go to bed at 9, go at 10', it's too much to go to bed at 9. So she told me to go to bed at 10. So that's helpful to know, I thought I'd have to go to bed earlier, you know to sleep more hours, but that's not the case. […] she said to stay at 10 o'clock, because it's too much sleep otherwise, which is true isn't it really when you think about it." (post-therapy qualitative interview)<br><br>"…but it has changed the way I think about sleep. My approach to sleep it's changed." (post-therapy qualitative interview)
Learning was valued in itself	**"Interviewer: do you feel there was anything you enjoyed about being part of the L-DART project?**<br>Participant: It was ok doing it, learning about sleep and everything it felt good." (post-therapy qualitative interview)
Even when sleep pattern had not changed significantly, improved understanding was valued	"I'm more aware you know, and my sleep is still erratic but I am nevertheless aware that around half-two for some reason, three o'clock in the morning I ought to be sleeping […] and also the notion of using the light as a, almost control system or a prompt for your body to get into a rhythm in the morning, you know so the whole lesson […] has been digested by me, but I'm just not using it at the moment, but I do feel that it's been of value..." (post-therapy qualitative interview)<br><br>"**Interviewer: …anything in particular you remember that [the therapist] had kind of talked through with you, that did feel quite helpful for you?**<br><br>Participant: Just that daylight is better for you than night-light. That's what she taught me, daylight is more helpful […] so it has helped me in a way being awake during the day. […] I've learnt a little bit from doing the project, as to how to get a good nights sleep, and be up during the

	daylight which makes you feel better." (post-therapy qualitative interview)

Outcomes

Theme/sub-theme	Data extracts/evidence
**Improved sleep and functioning**<br><br>A range of improvements were described, especially improved perceived sleep quality and daytime functioning<br><br>No participants described the therapy making their sleep worse	"…eventually it clicked you know and like I say I have been sleeping through better. Been awake a couple of times a night, but a lot less than what it was." (post-therapy qualitative interview)<br><br>"The quality of sleep I get is good, really good. I might not be asleep for long, but when I am asleep I do get the benefit of it." (post-therapy qualitative interview)<br><br>"Since what she said and stuff I don't have as many [nightmares] now." (post-therapy qualitative interview)<br><br>"Well there's been positive results too generally, it's just that this has fallen flat over the past few weeks with this situation [external stressor] diverting me from the regularity that we were speaking about a minute ago, yeah." (post-therapy qualitative interview)<br><br>"I'm only having a small amount of sleep but it's so much more beneficial, I feel so much more awake and

	alive compared to how I was. So, that's it." (post-therapy qualitative interview)<br><br>"Yeah I'm getting a lot longer, a lot longer sleep." (post-therapy qualitative interview)<br><br>"Since I've been getting up earlier I've taken the medication earlier, and there's more time for going out and making appointments for in the mornings, cos one time I could never make an appointment anywhere in the morning. But at this time now I can make more appointments and I can go out more. It's changed my life, this therapy." (post-therapy qualitative interview)
