# Supplementary material for: Light–Dark and Activity Rhythm Therapy (L-DART) to Improve Sleep in People with Schizophrenia Spectrum Disorders: A Single-Group Mixed Methods Study of Feasibility, Acceptability and Adherence
Source: Clocks Sleep. 2023 Dec 4;5(4):734–54. doi: 10.3390/clockssleep5040048 (PMC10742153; doi:10.3390/clockssleep5040048)

Participant 1

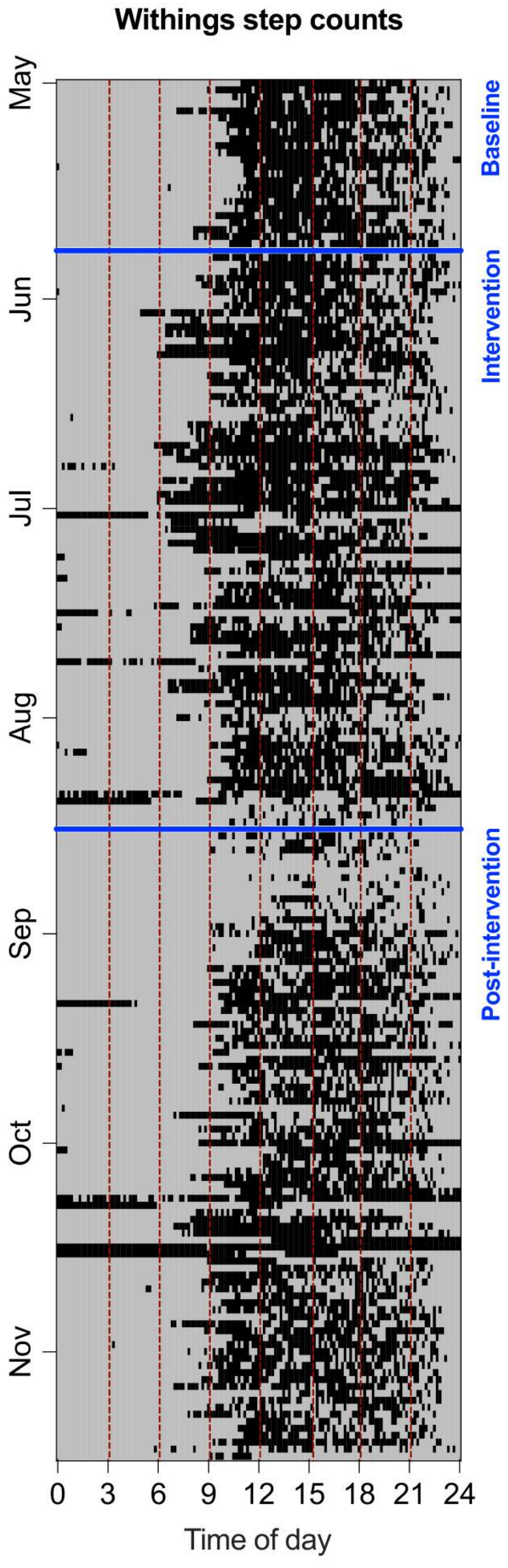

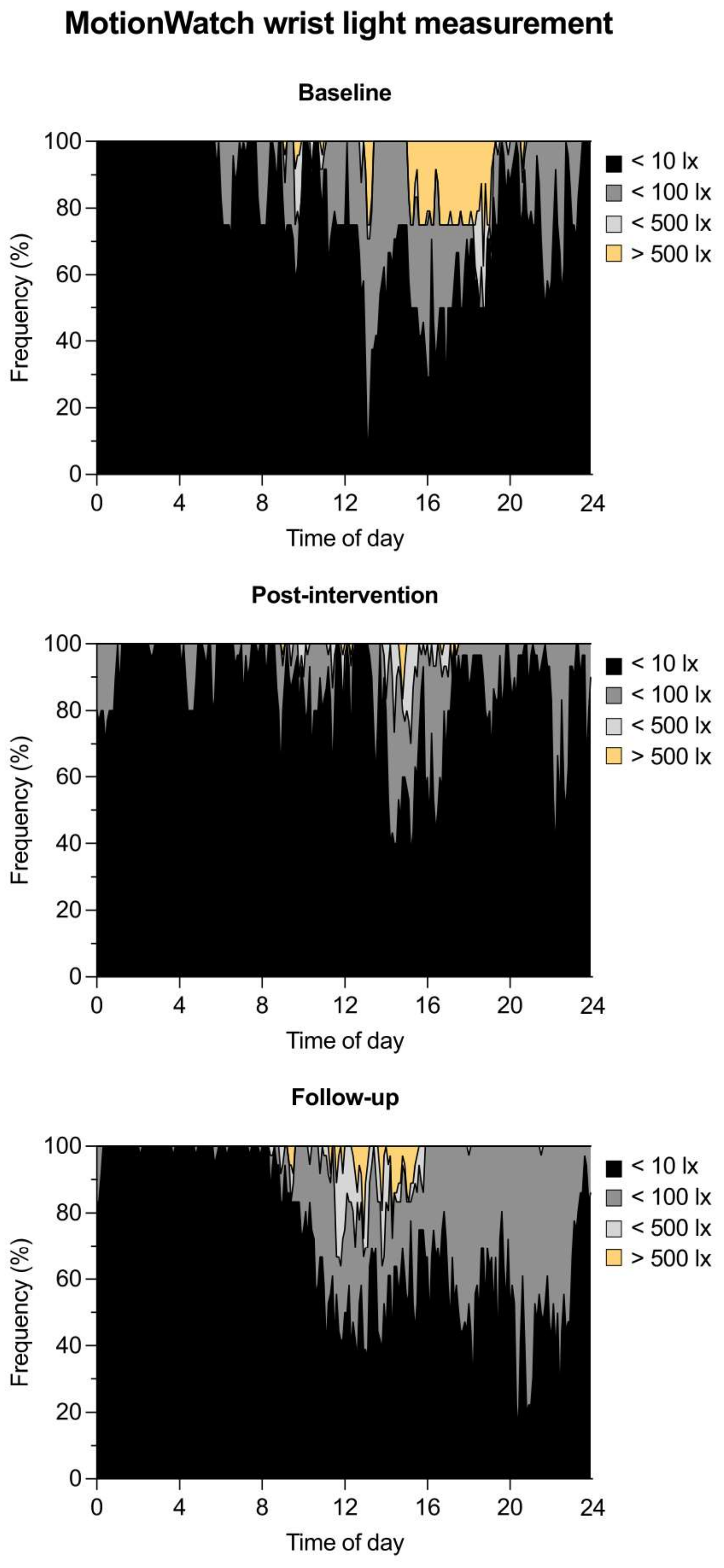

Participant 1

Bedroom

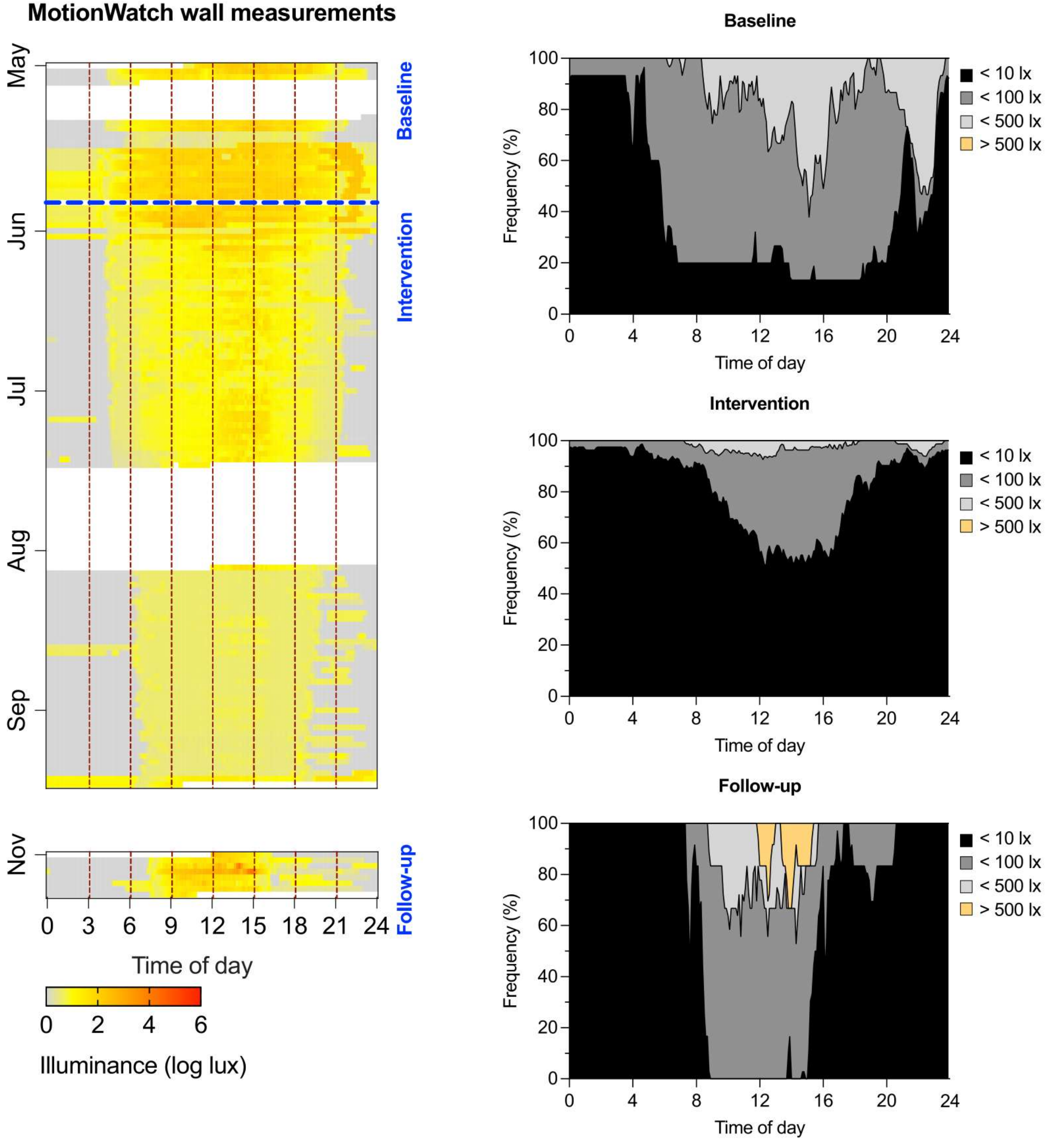

Participant 1

Living room

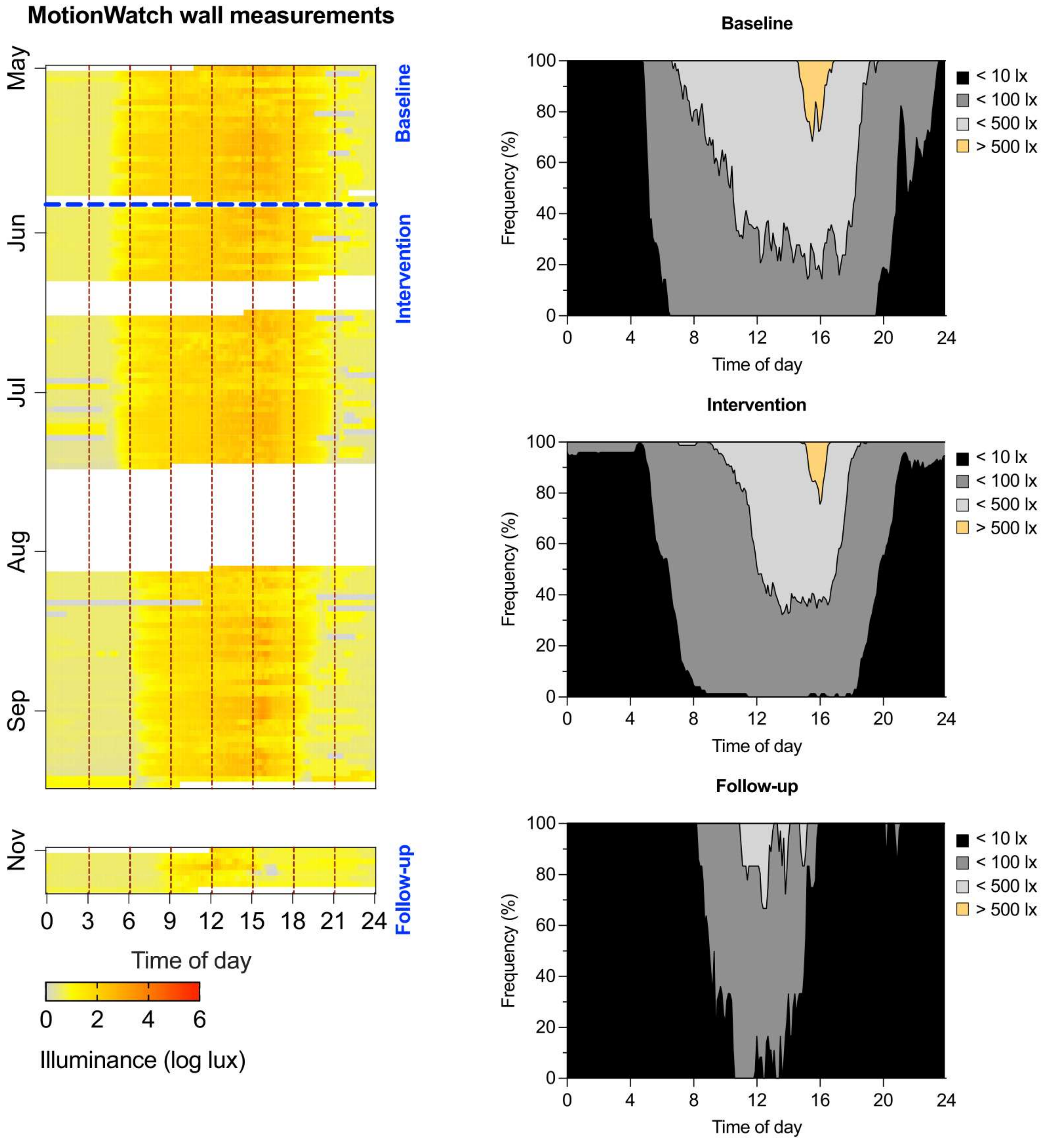

Participant 2

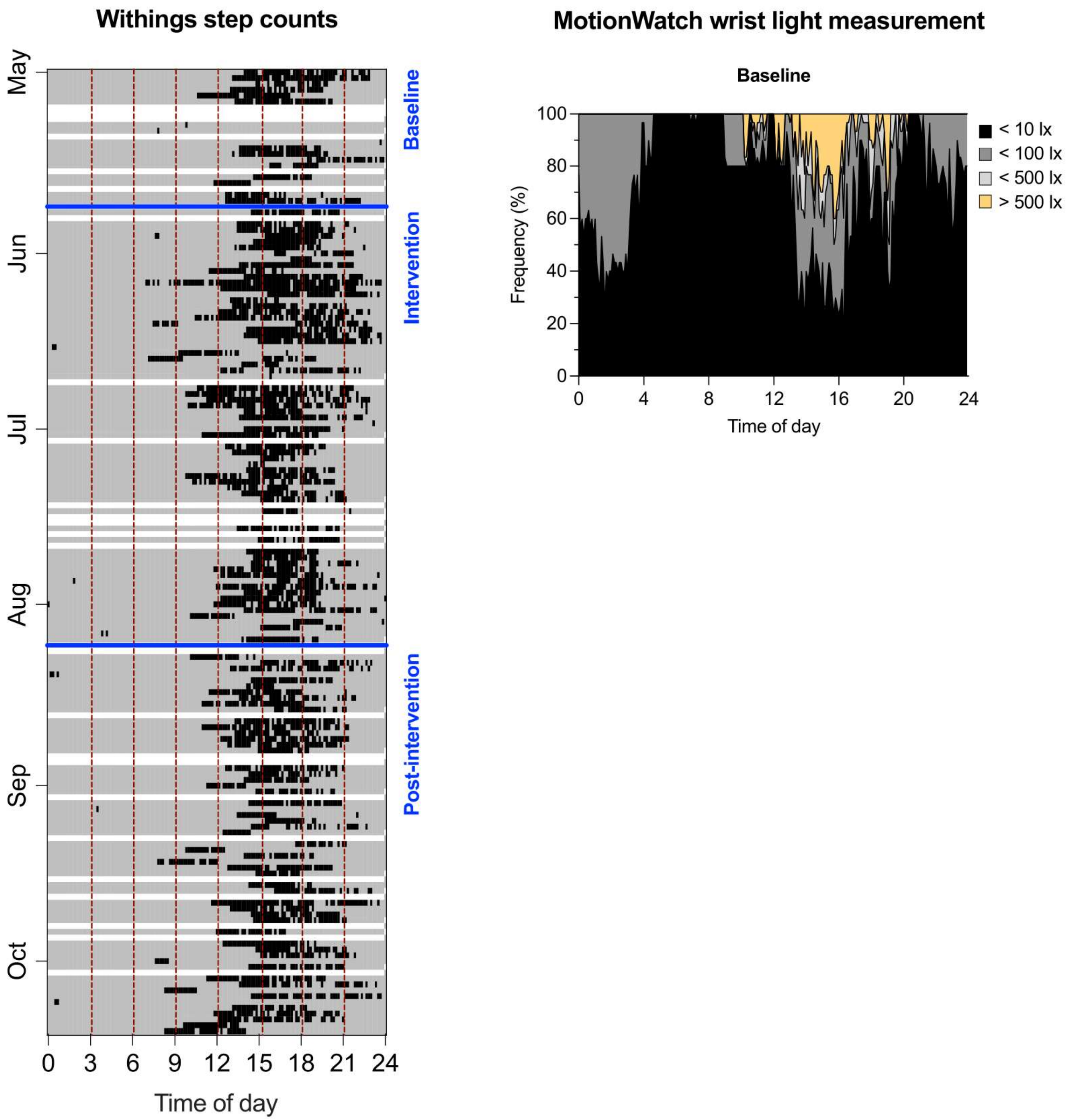

Participant 2

Bedroom

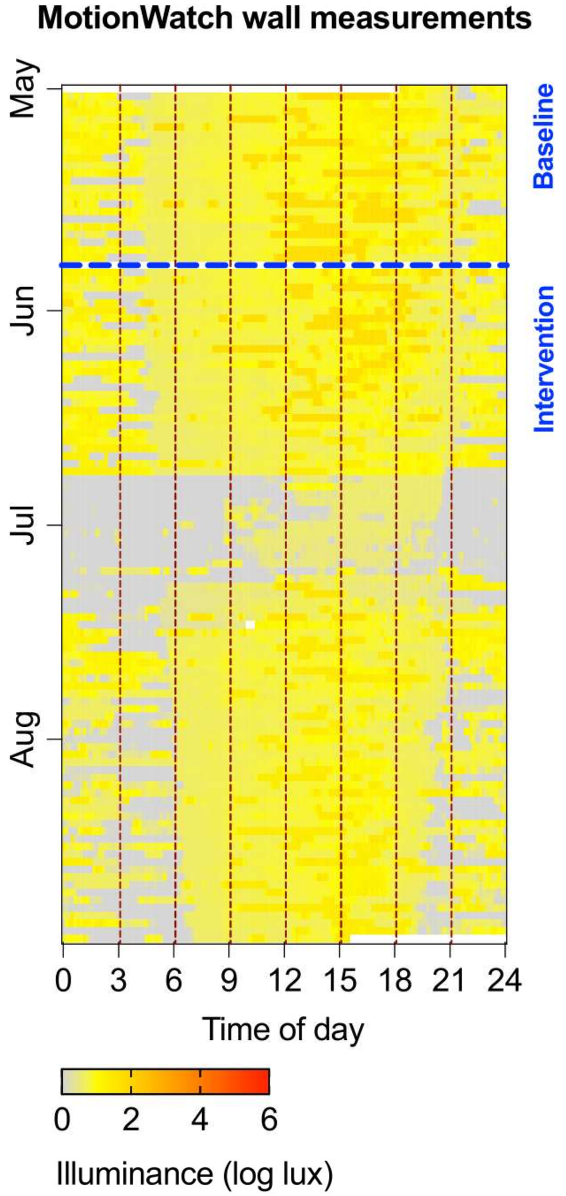

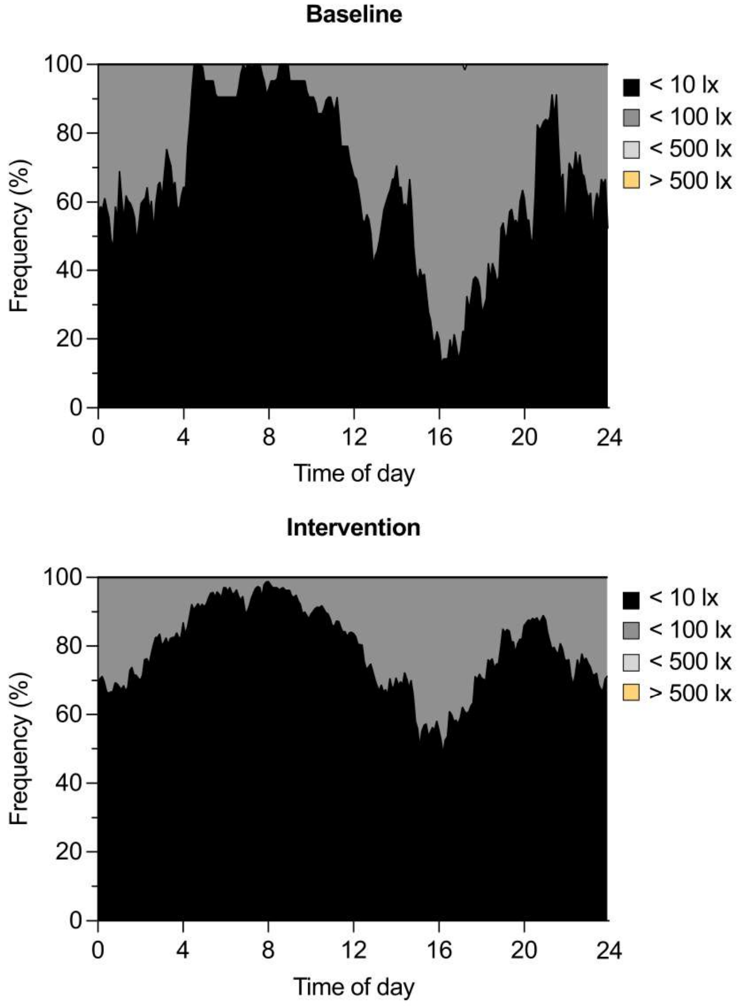

Participant 2

Living room

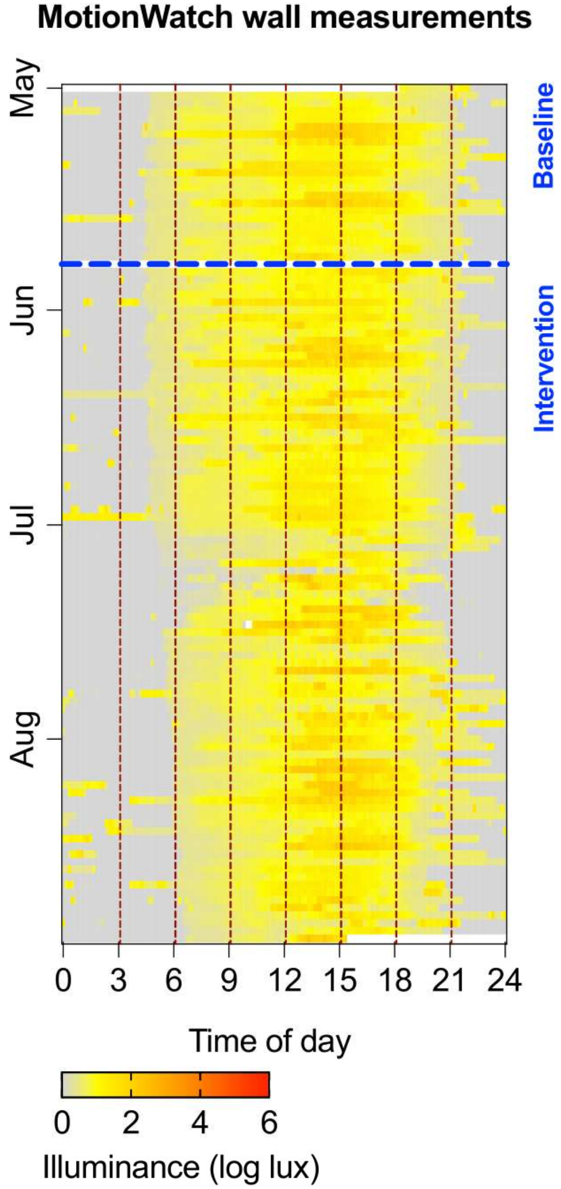

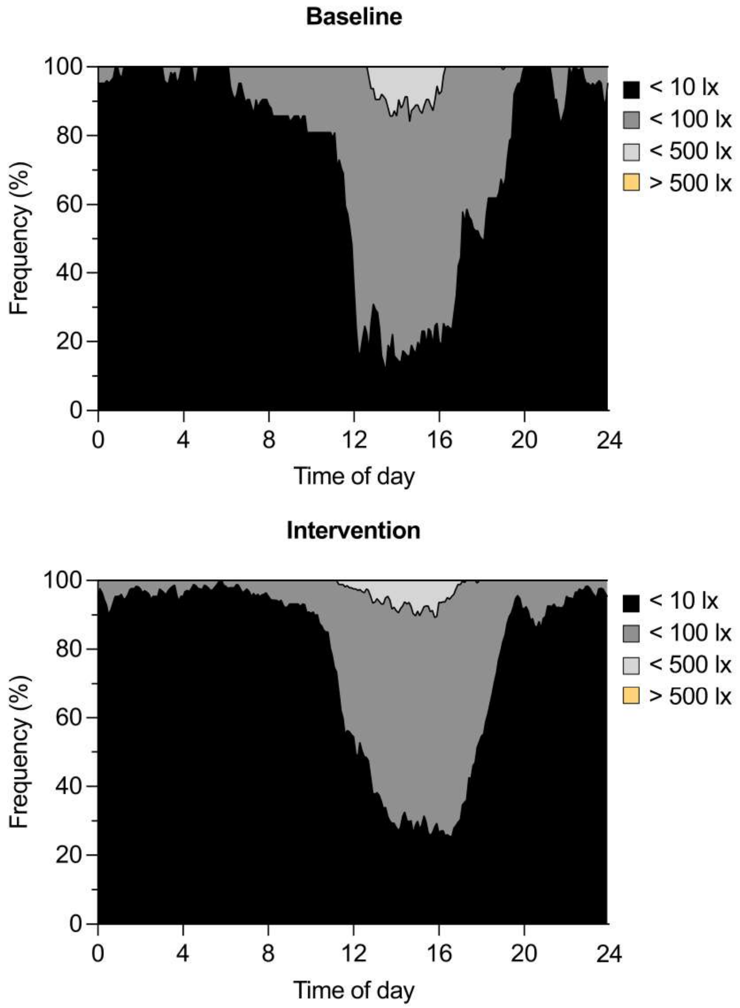

Participant 3

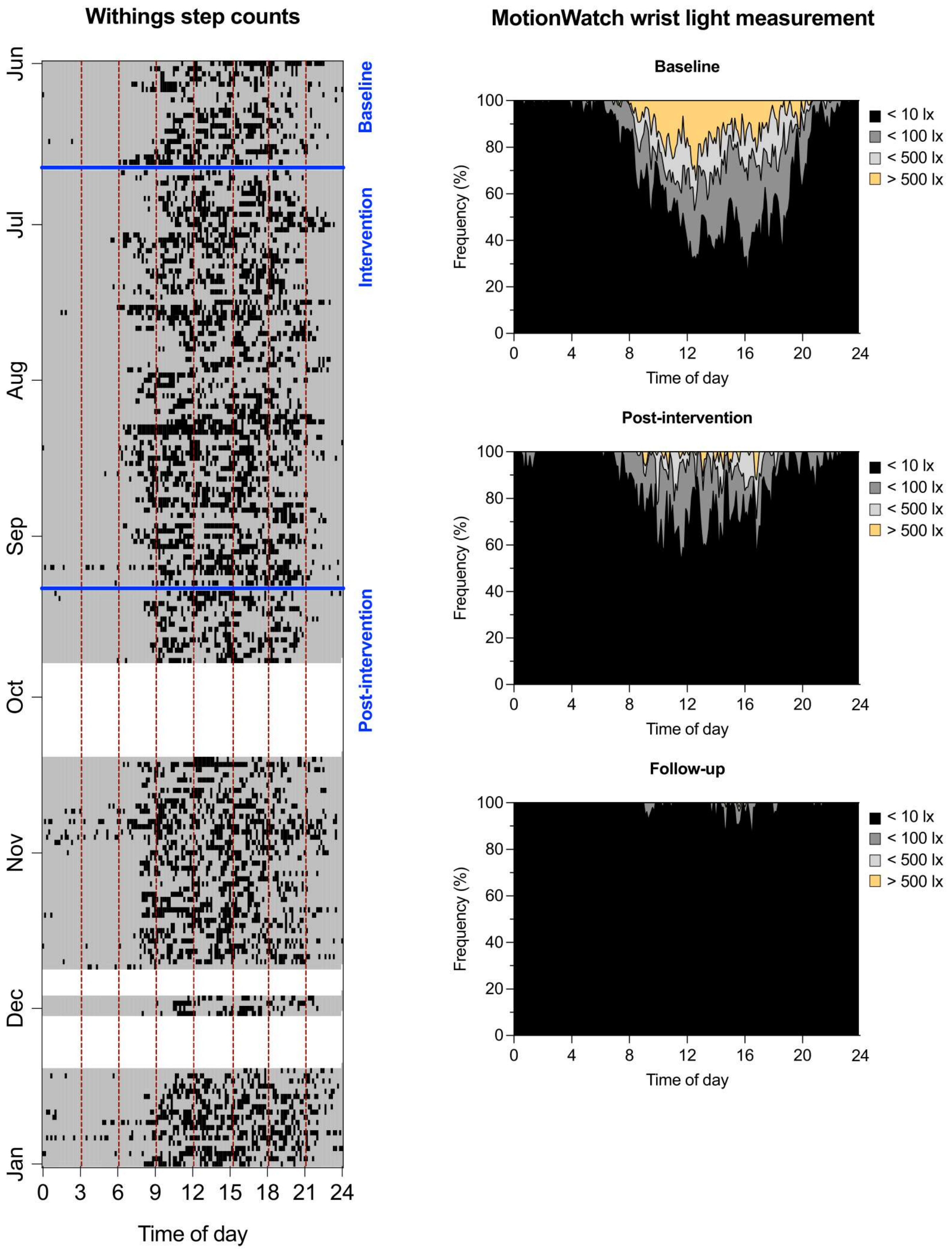

Participant 3

Bedroom

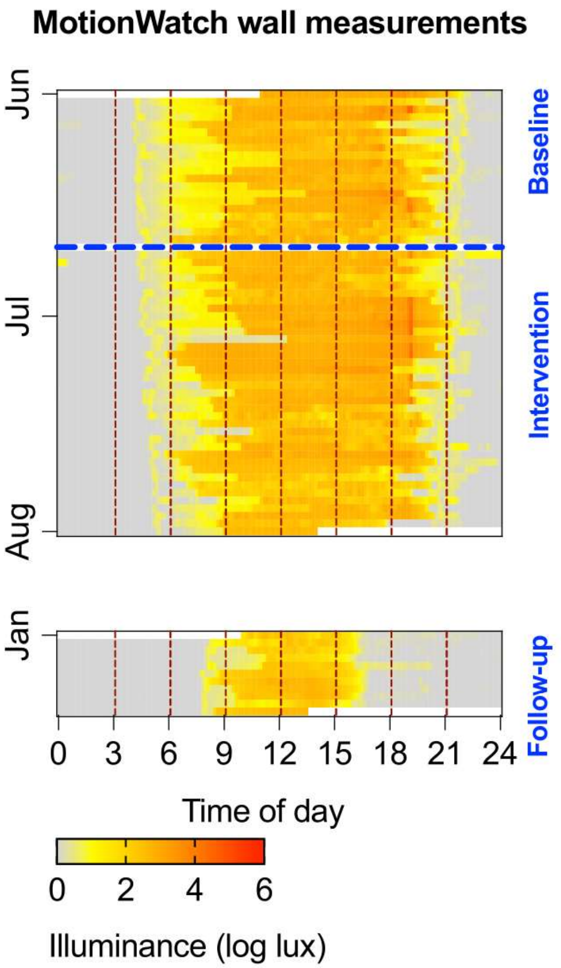

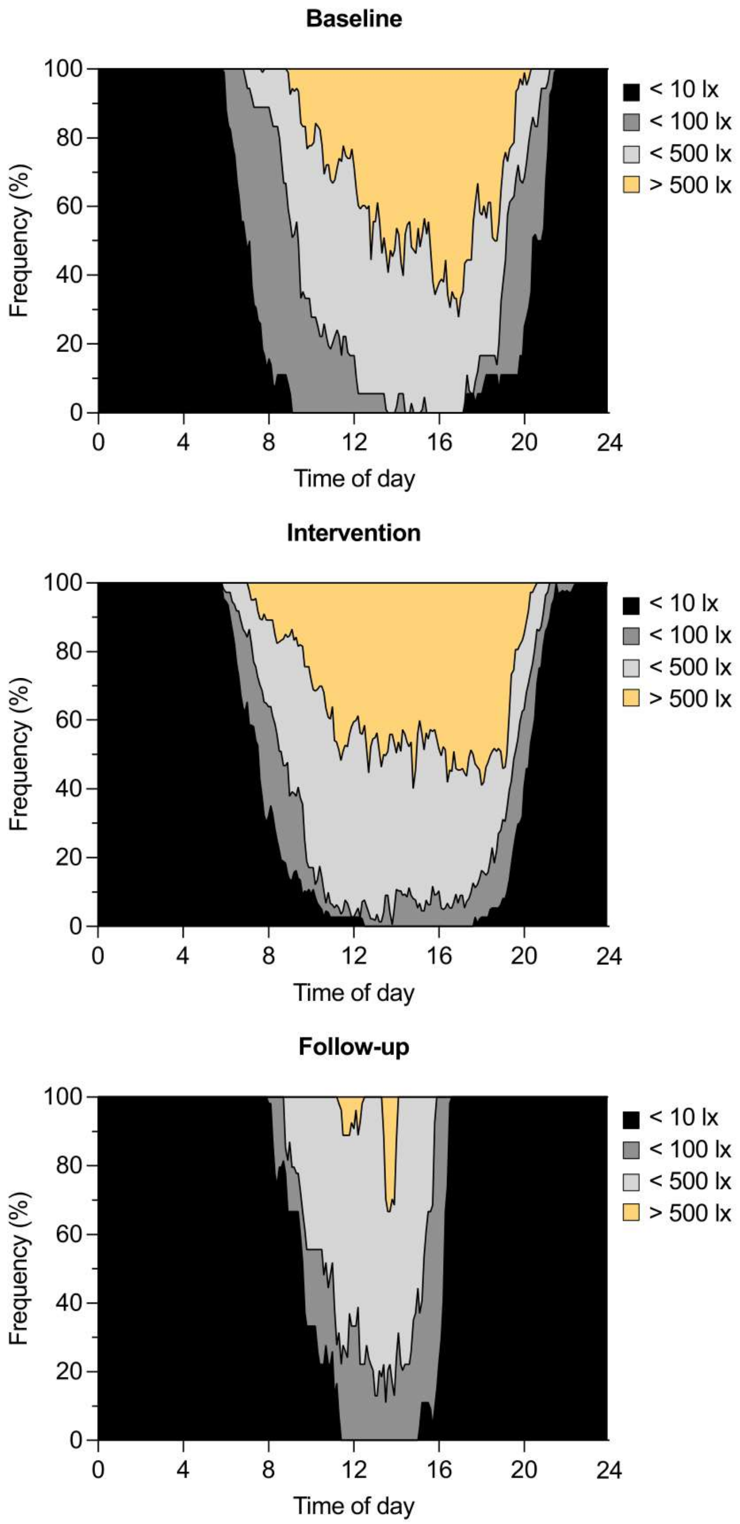

Participant 3

Living room

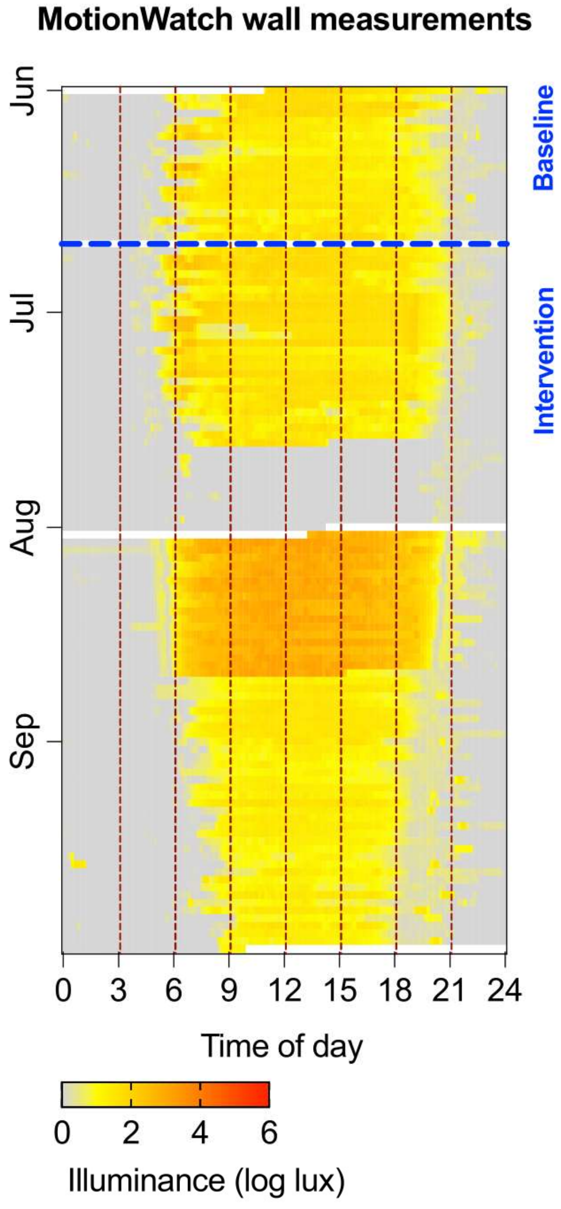

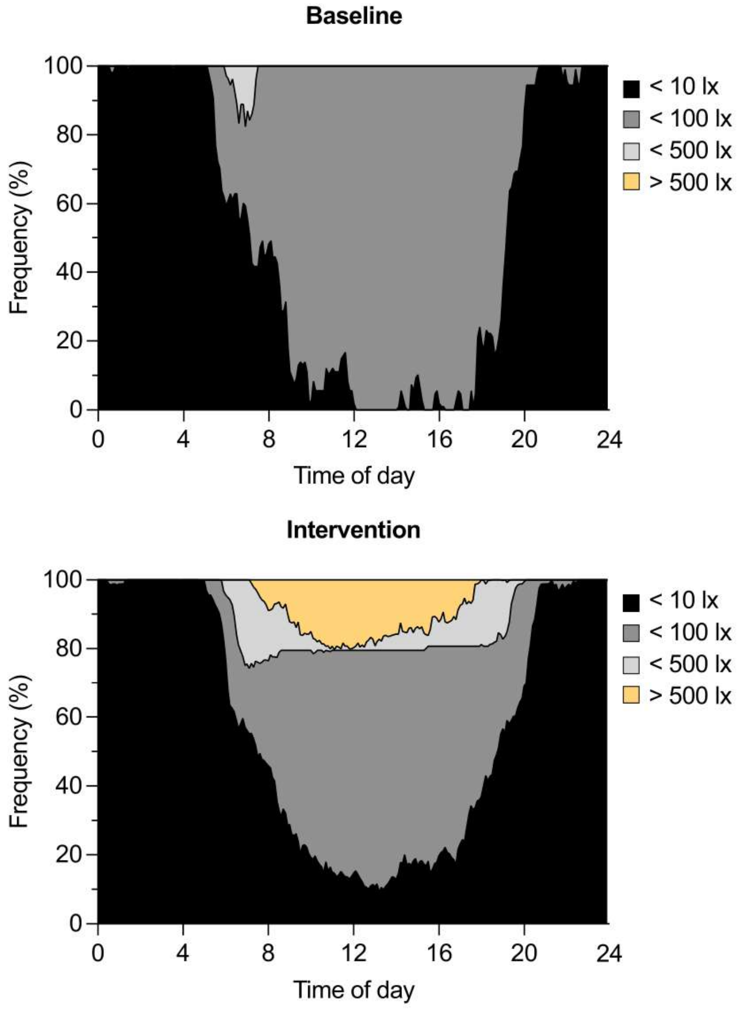

Participant 4

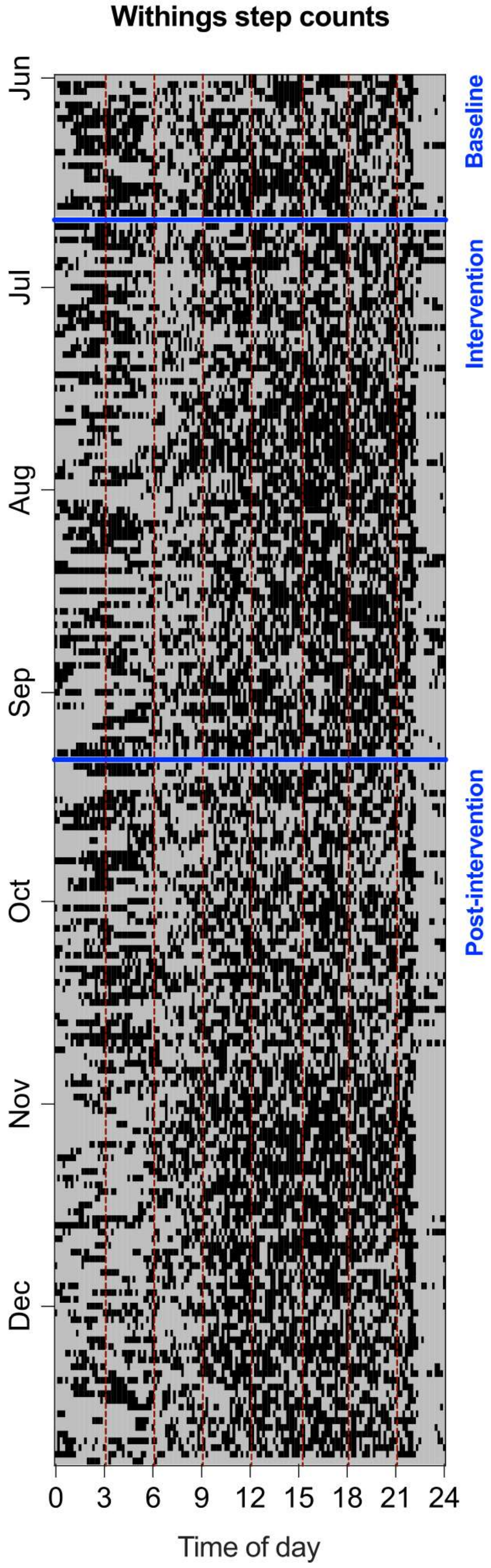

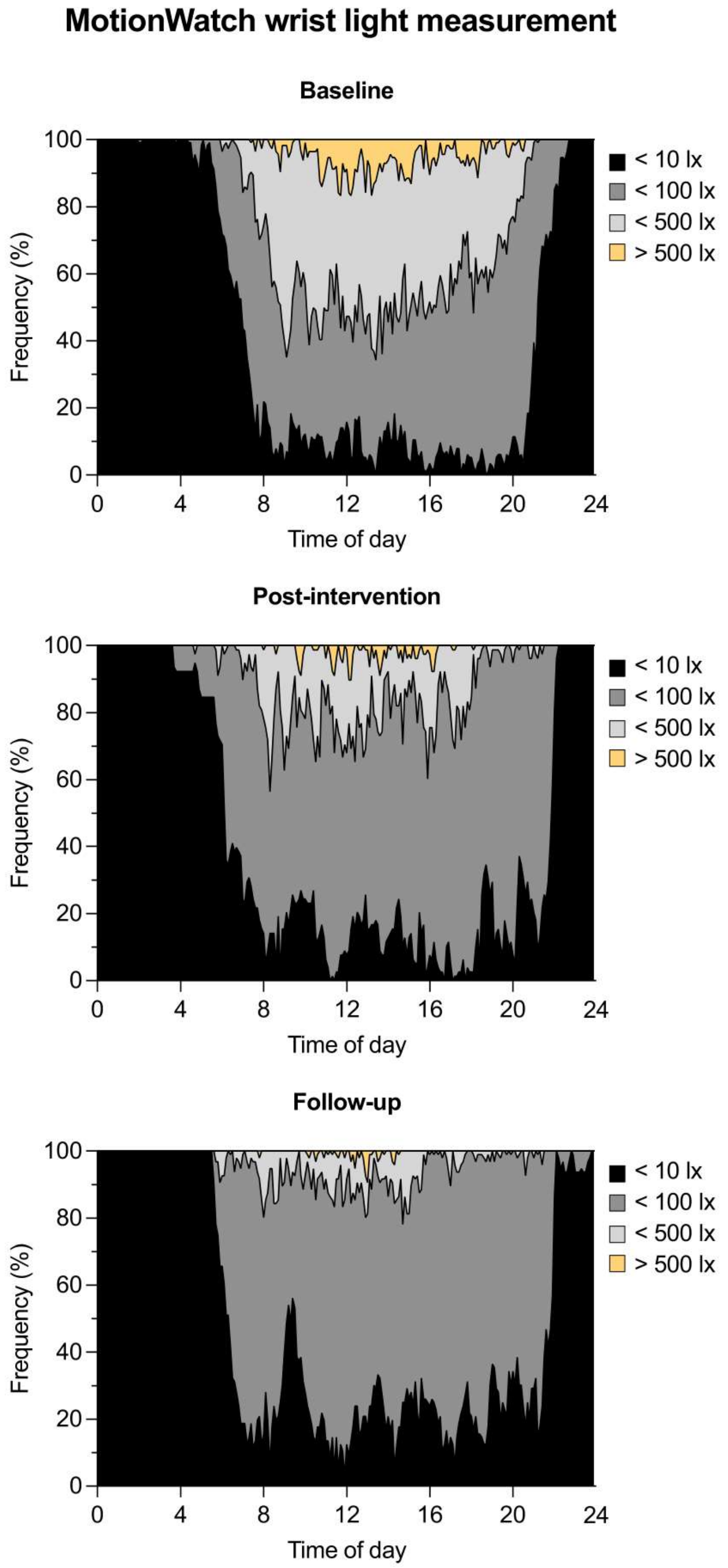

Participant 4

Bedroom

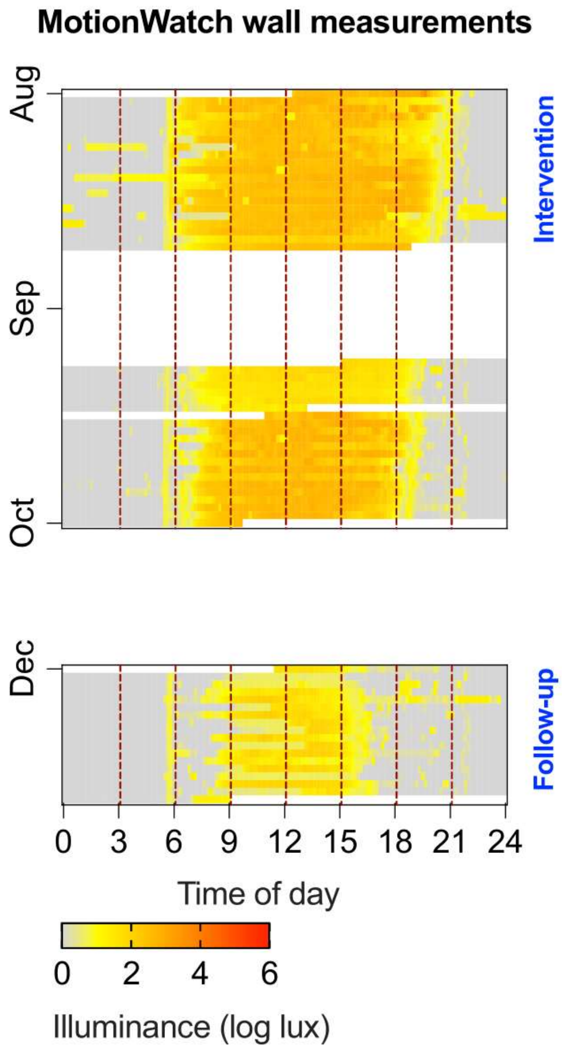

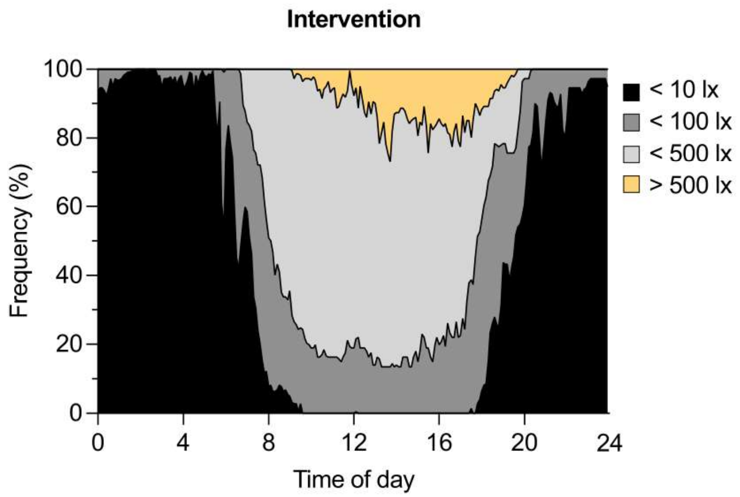

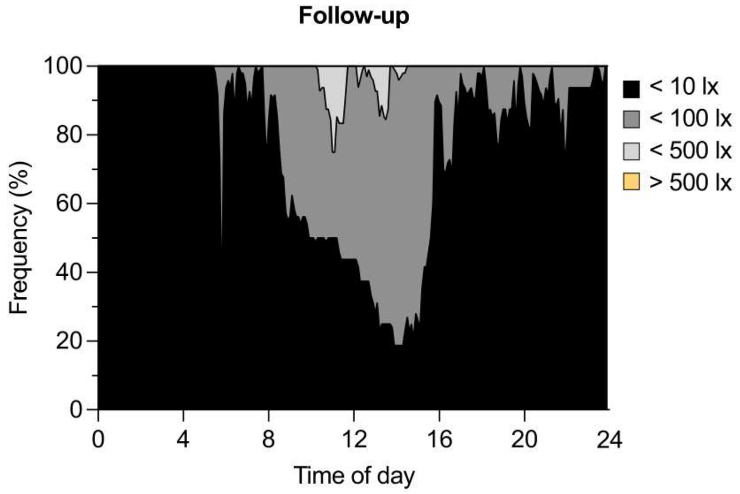

Participant 4

Living room

MotionWatch wall measurements

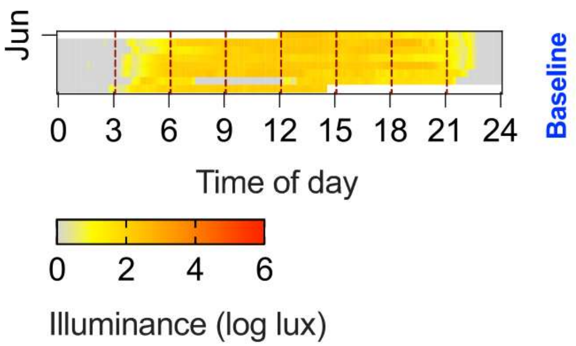

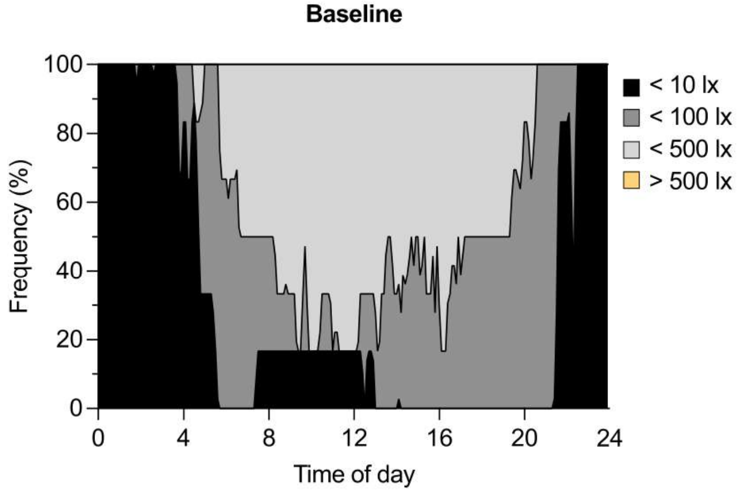

Participant 5

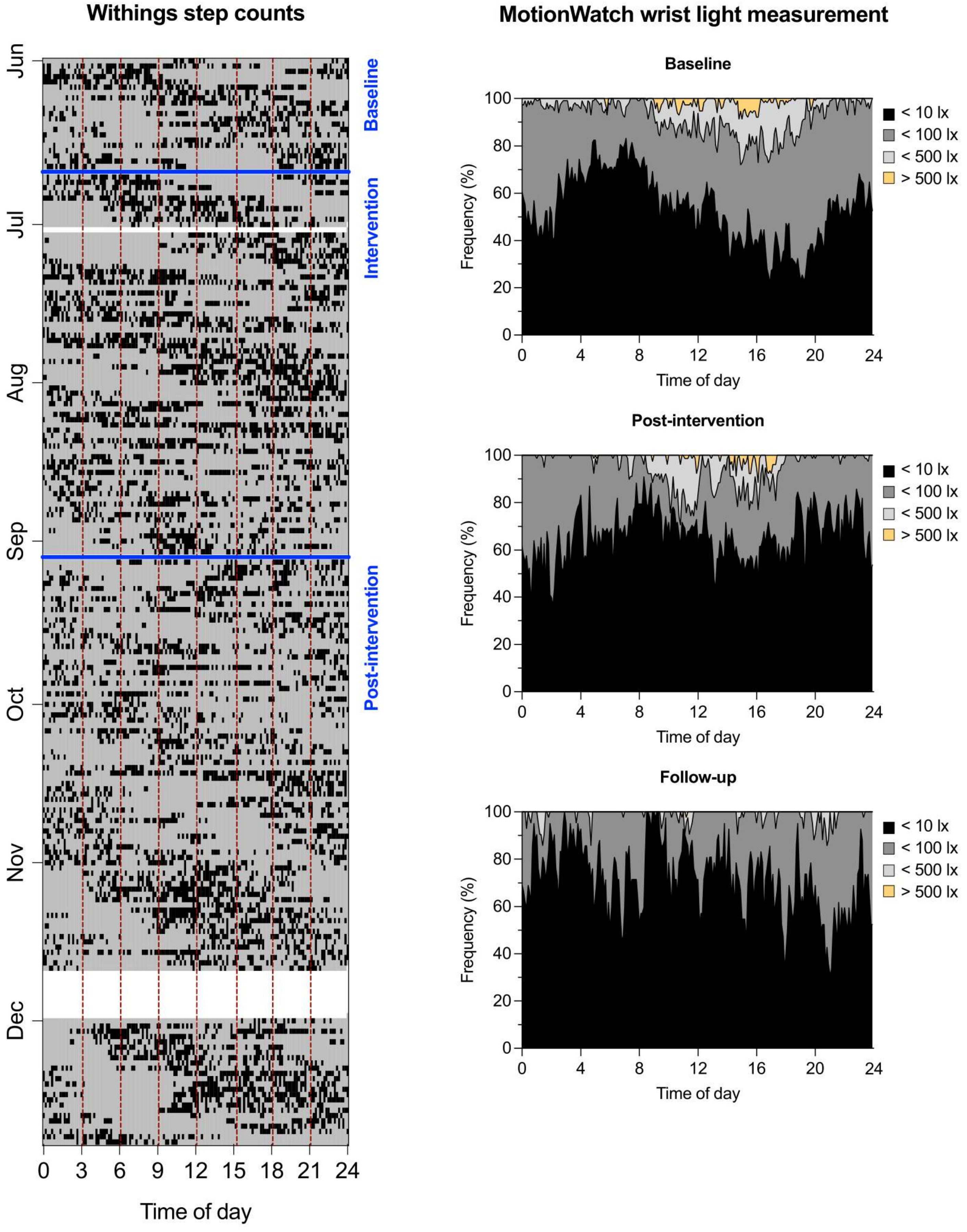

Participant 5

Bedroom

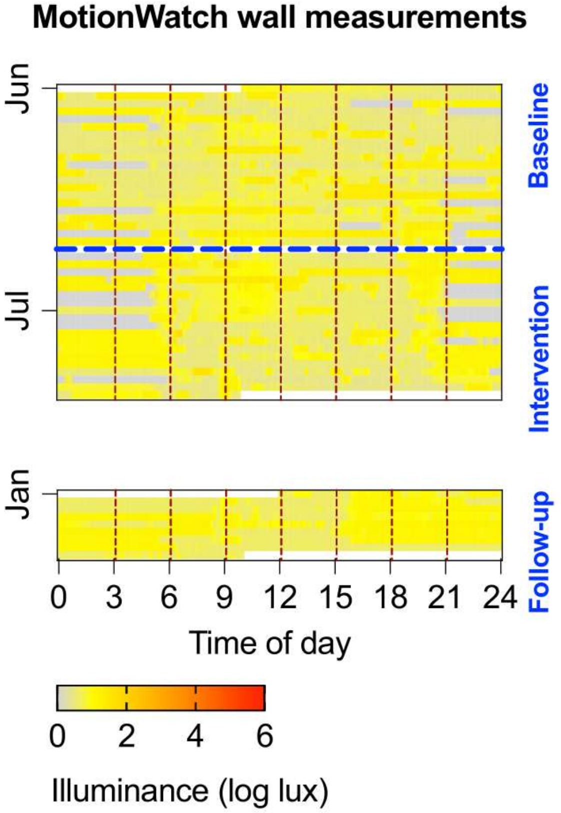

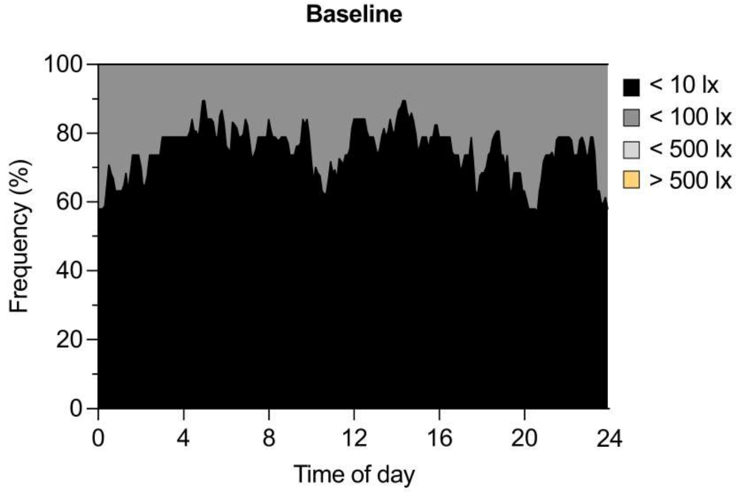

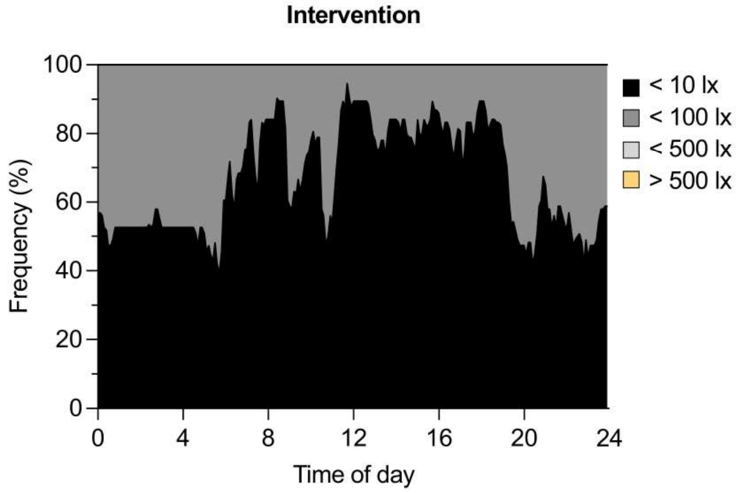

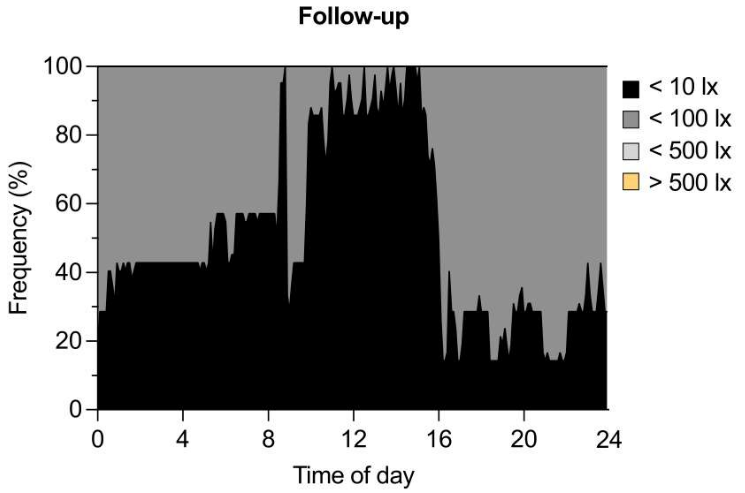

Participant 5

Living room

MotionWatch wall measurements

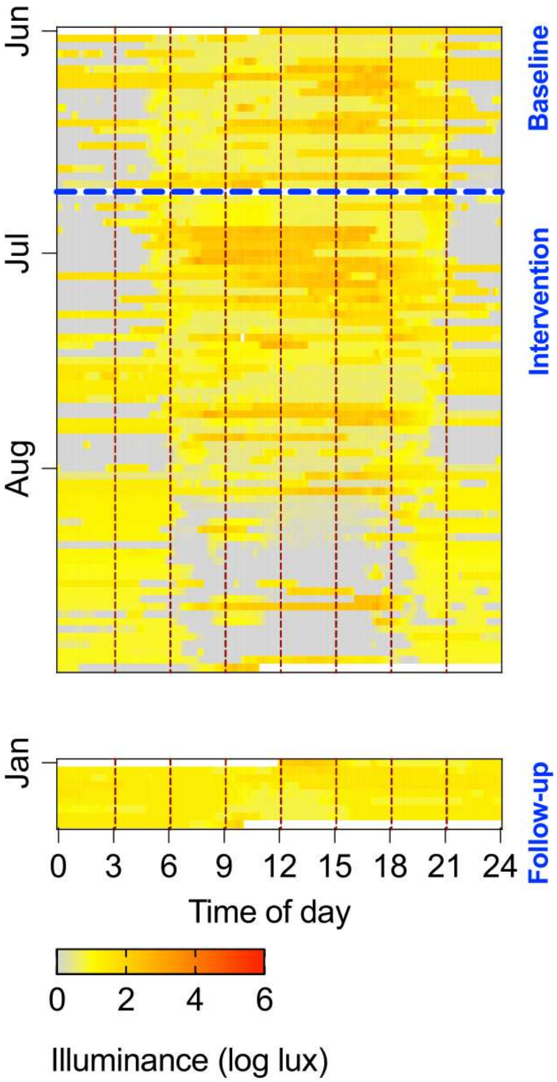

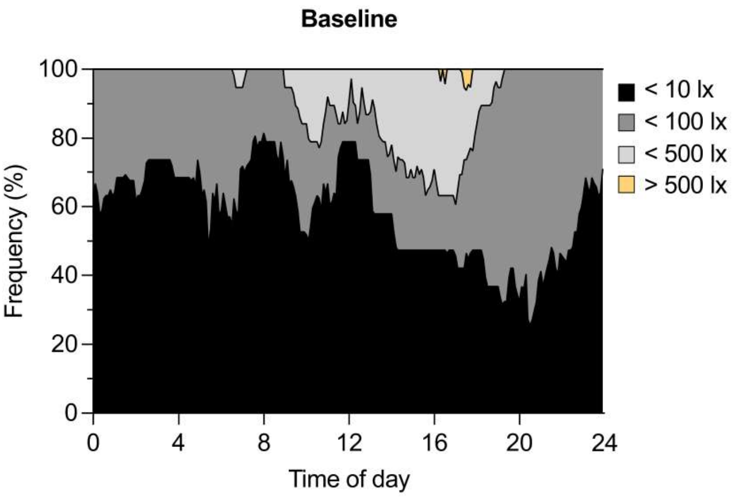

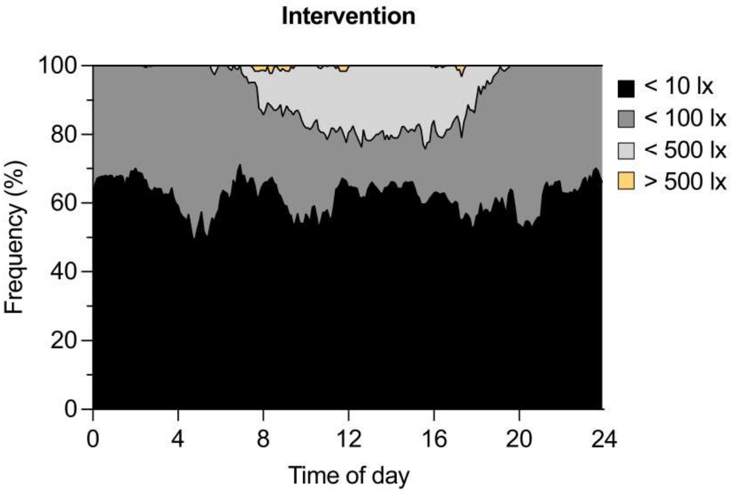

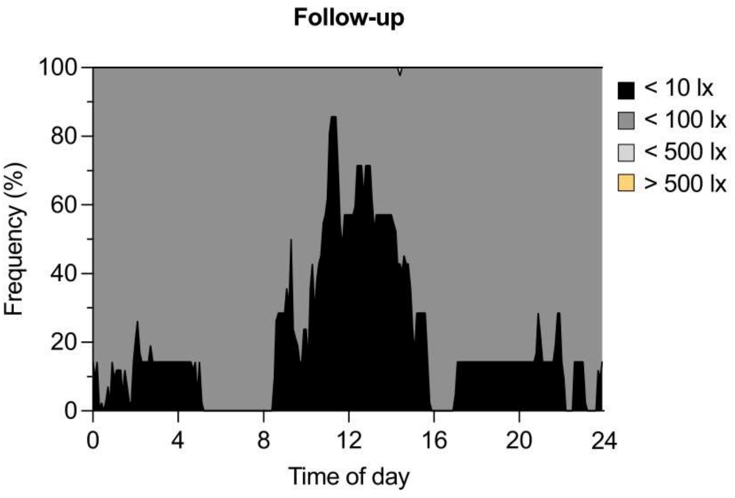

Participant 6

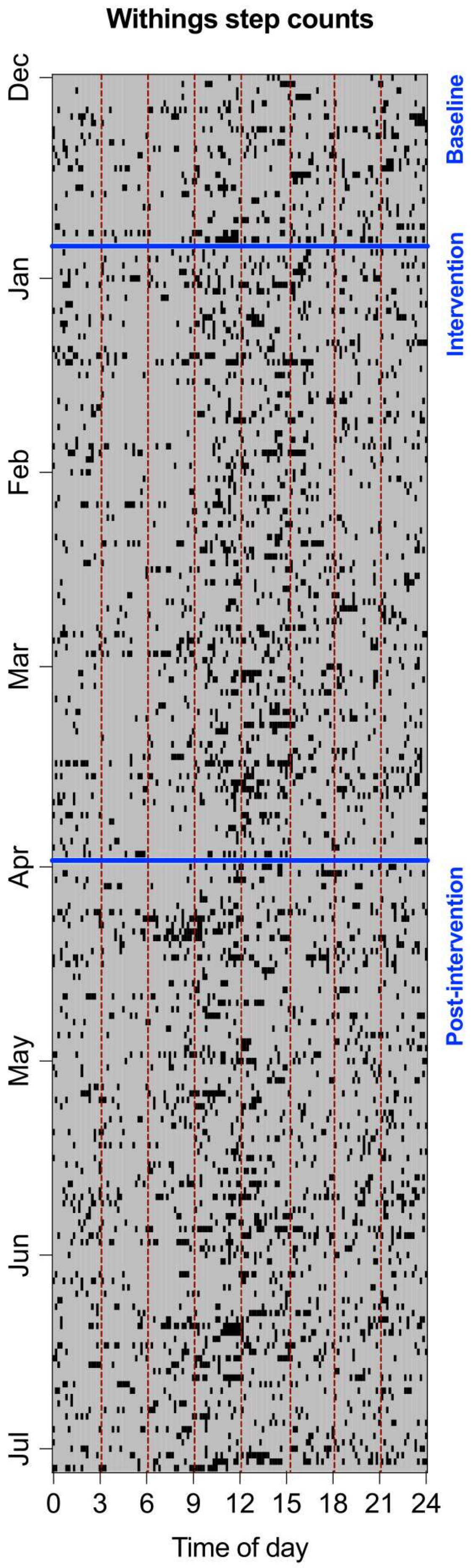

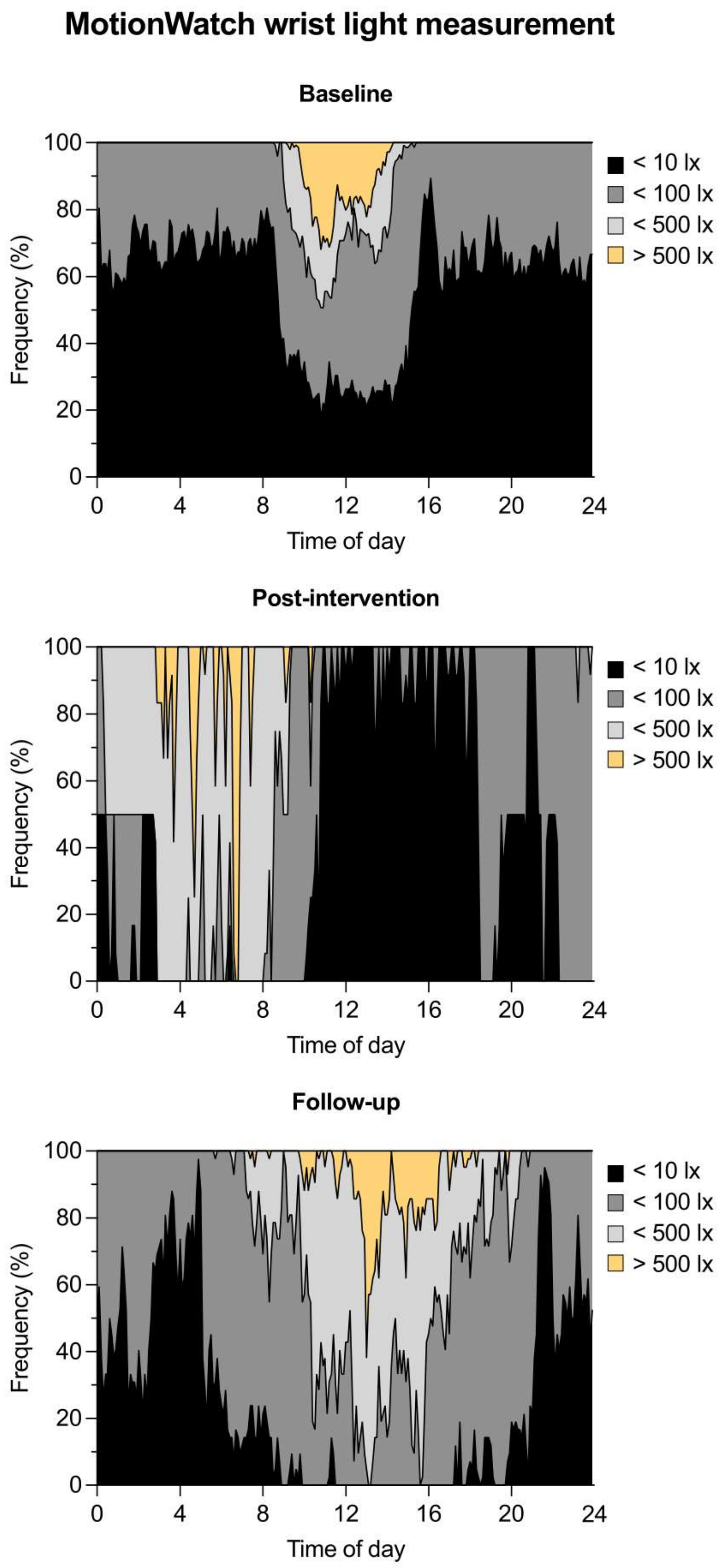

Participant 6

Bedroom

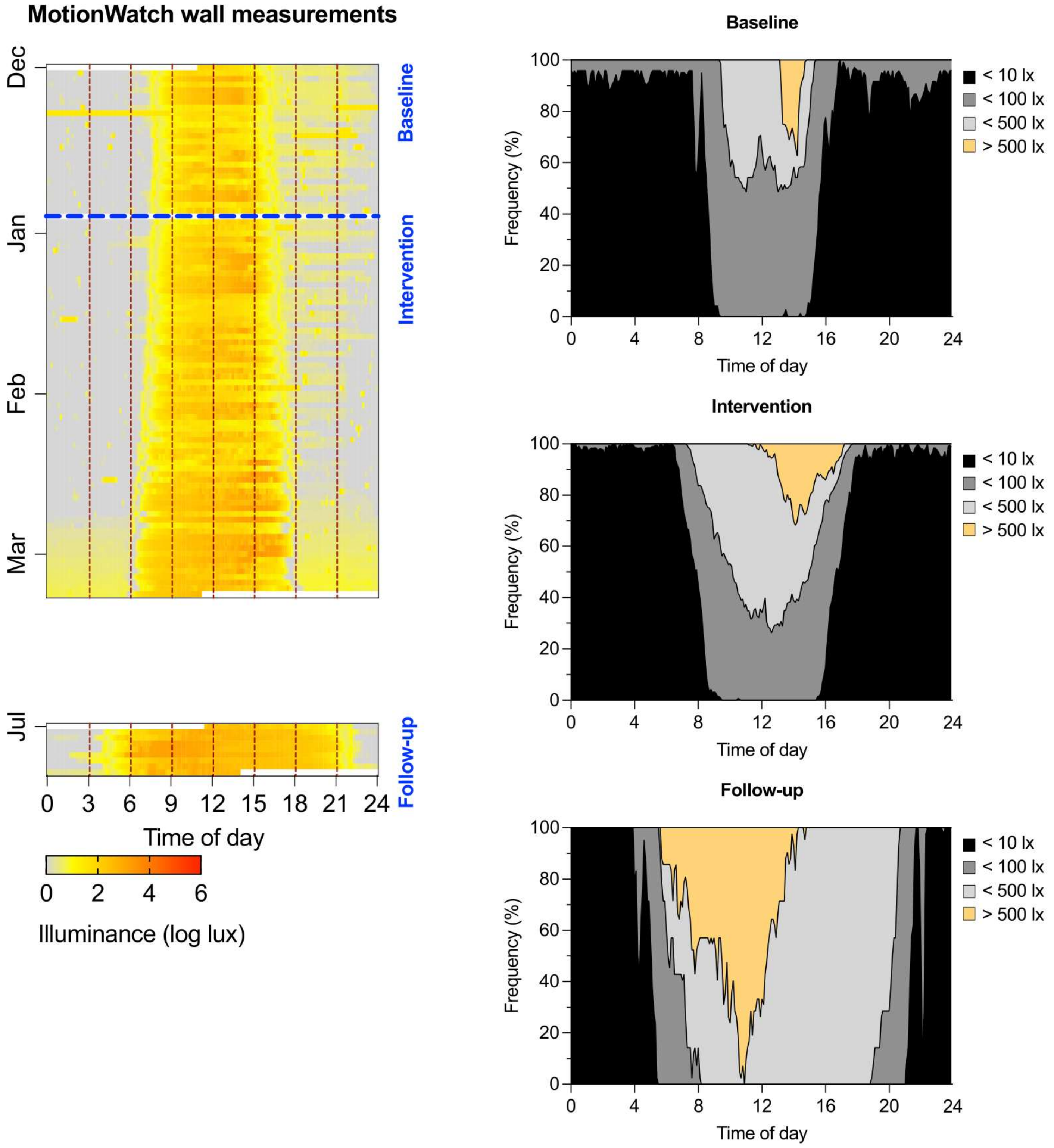

Participant 6

Living room

MotionWatch wall measurements

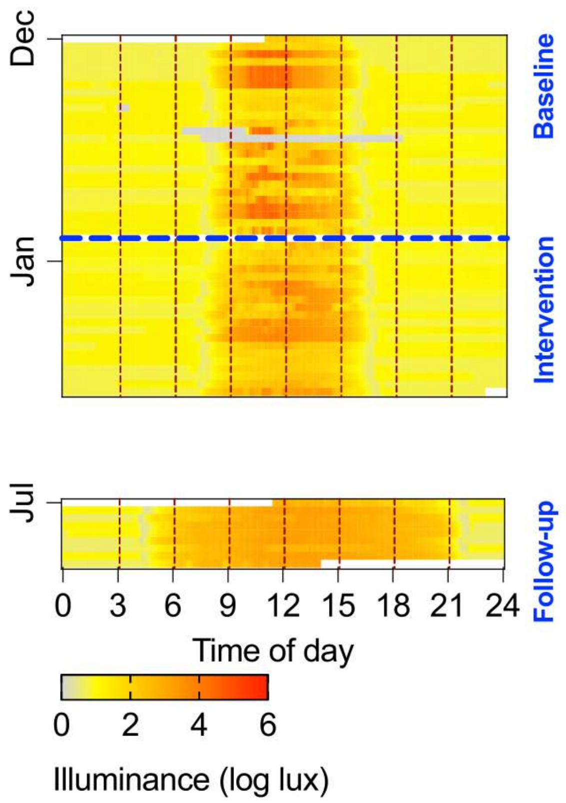

Baseline

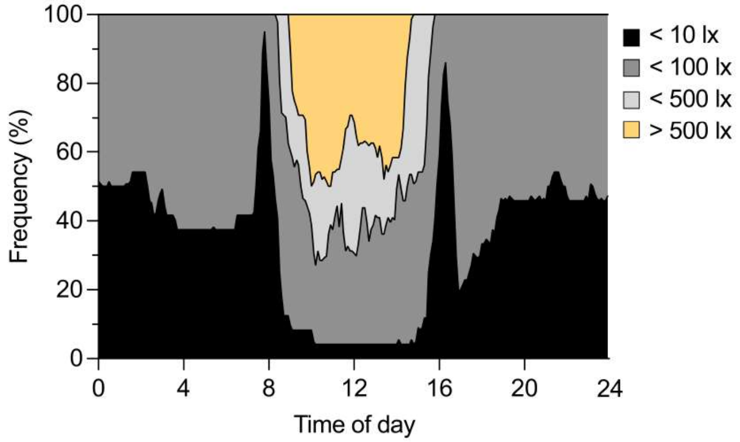

Intervention

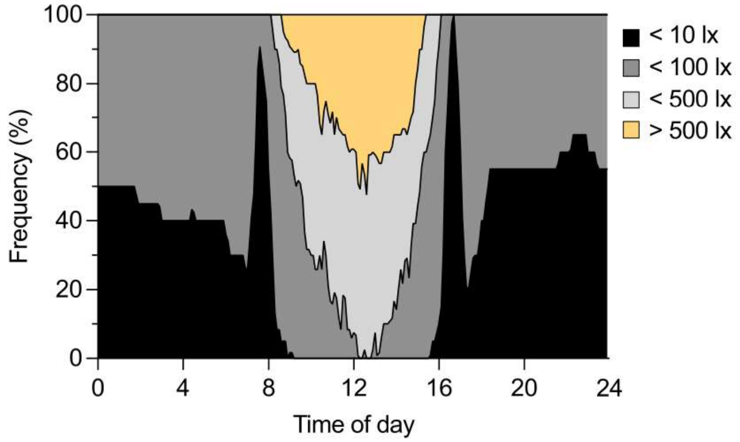

Follow-up

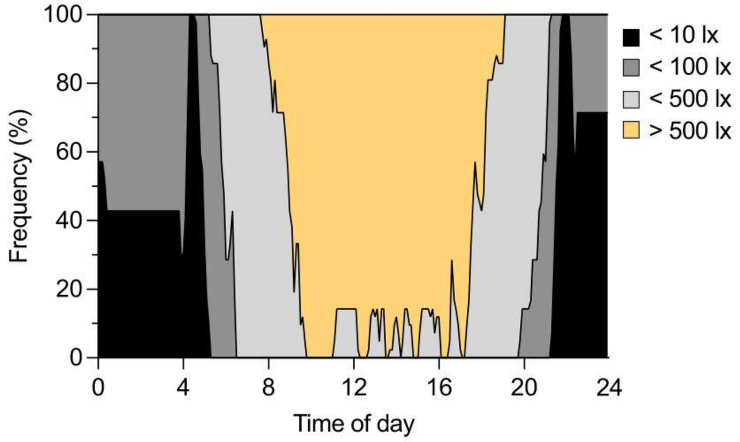

Participant 7

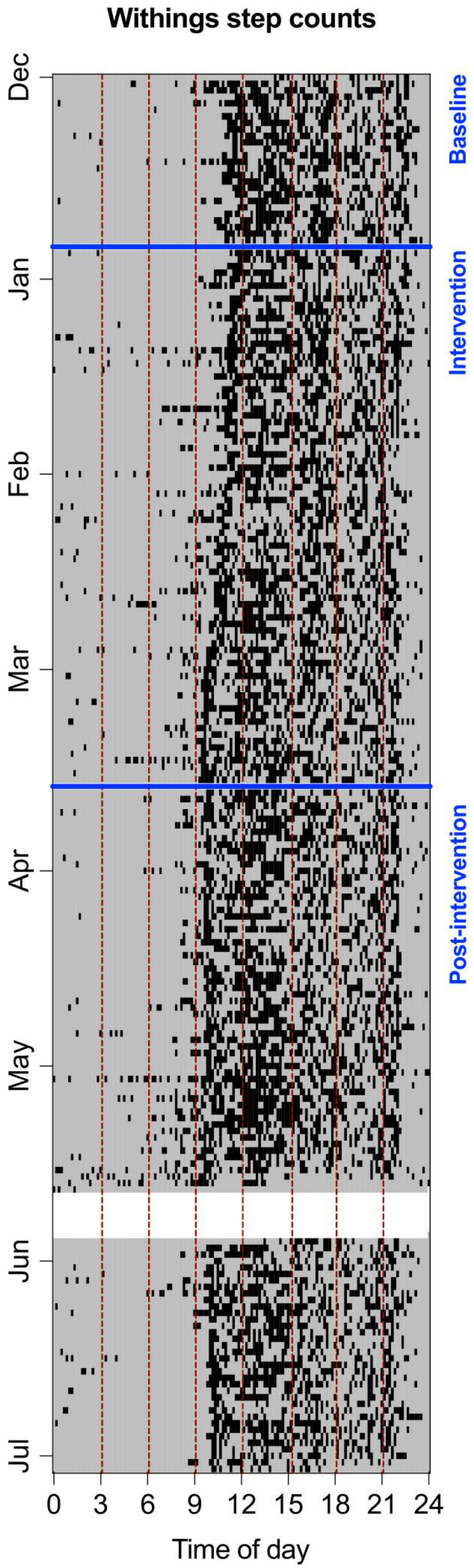

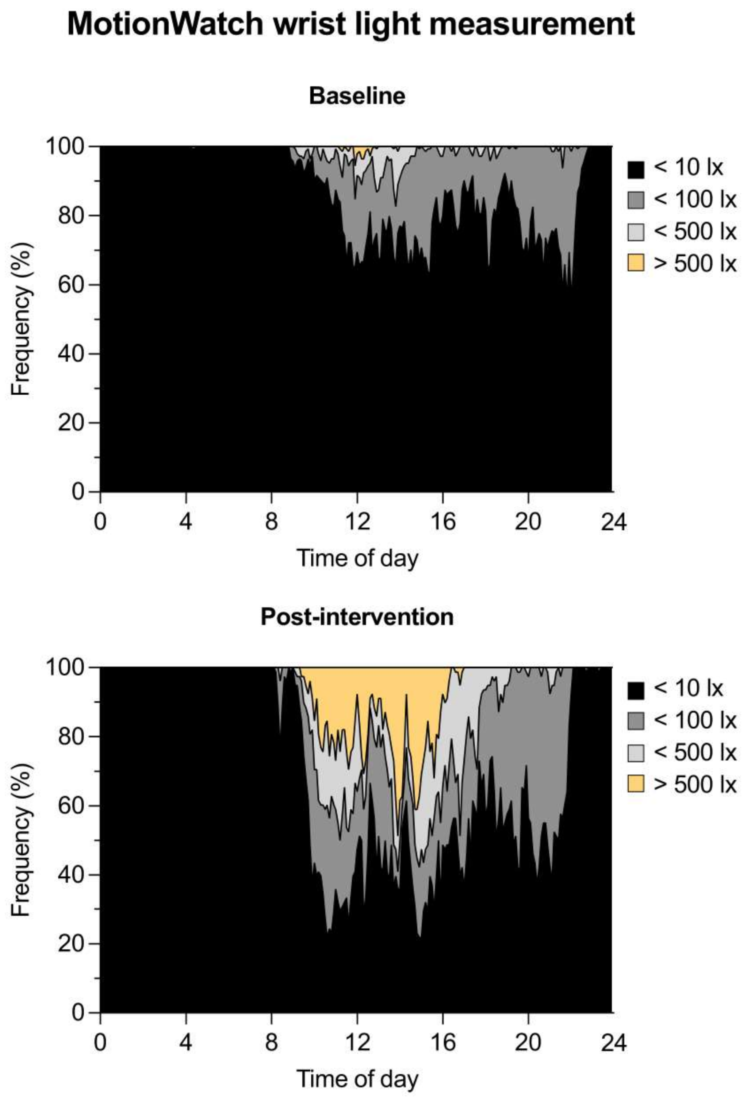

Participant 7

Bedroom

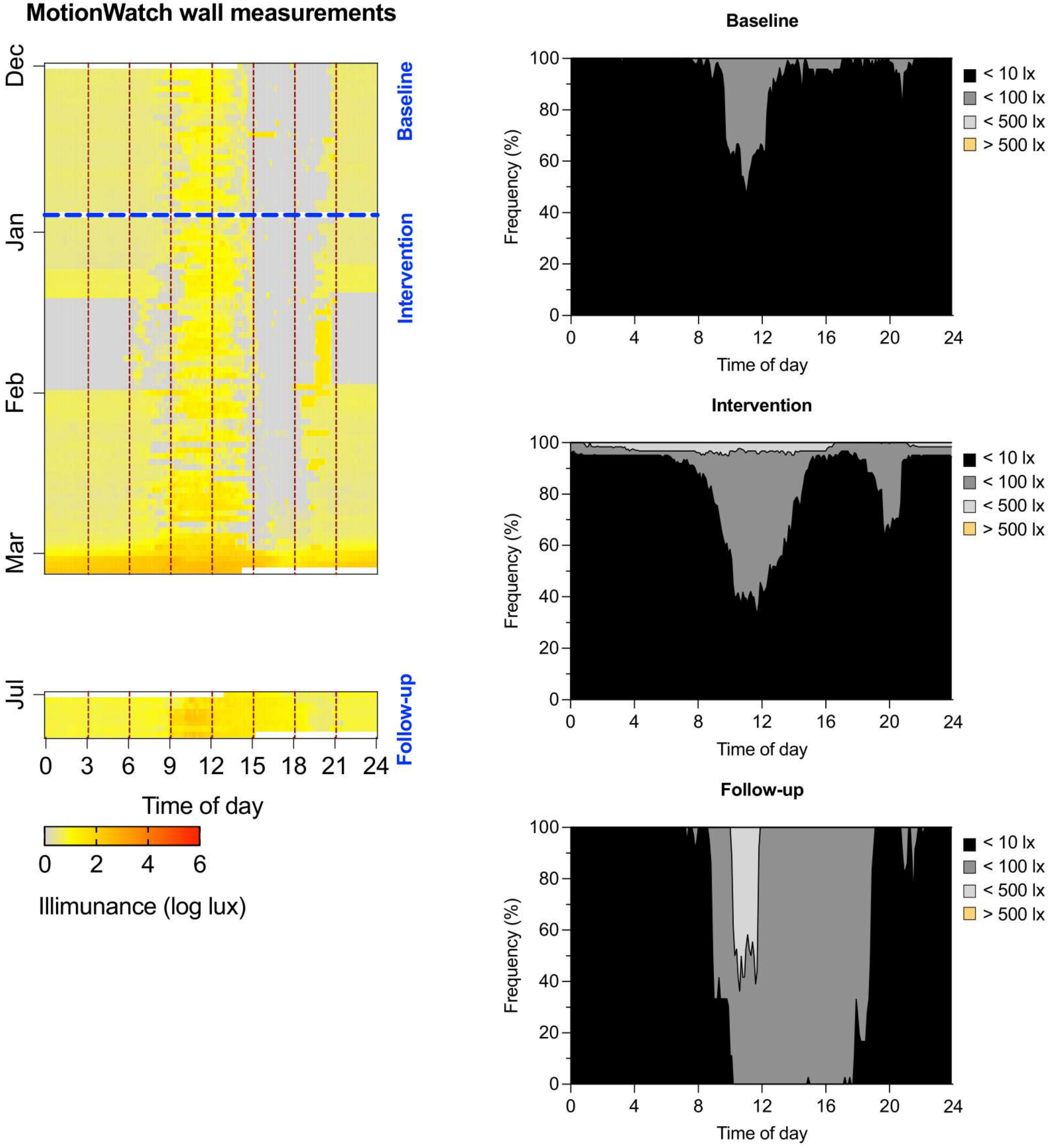

Participant 7

Living room

MotionWatch wall measurements

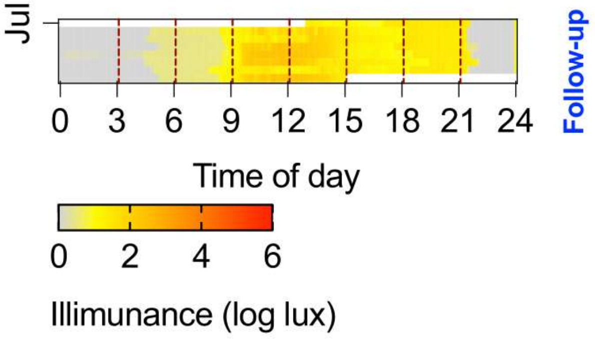

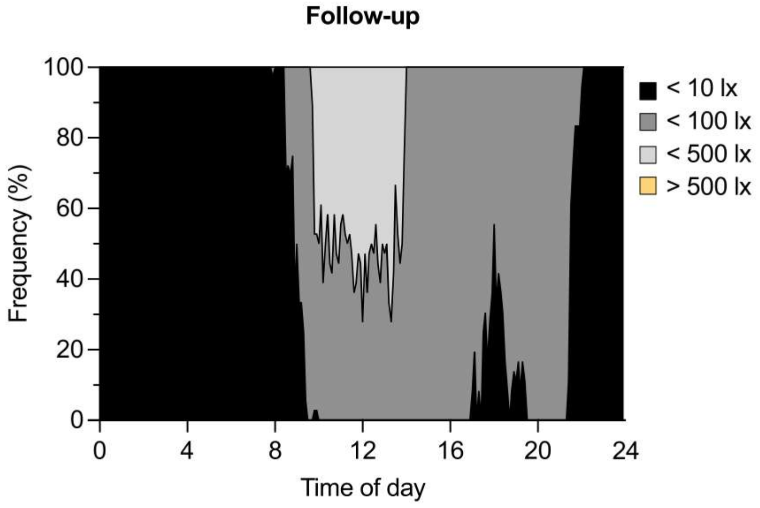

Participant 8

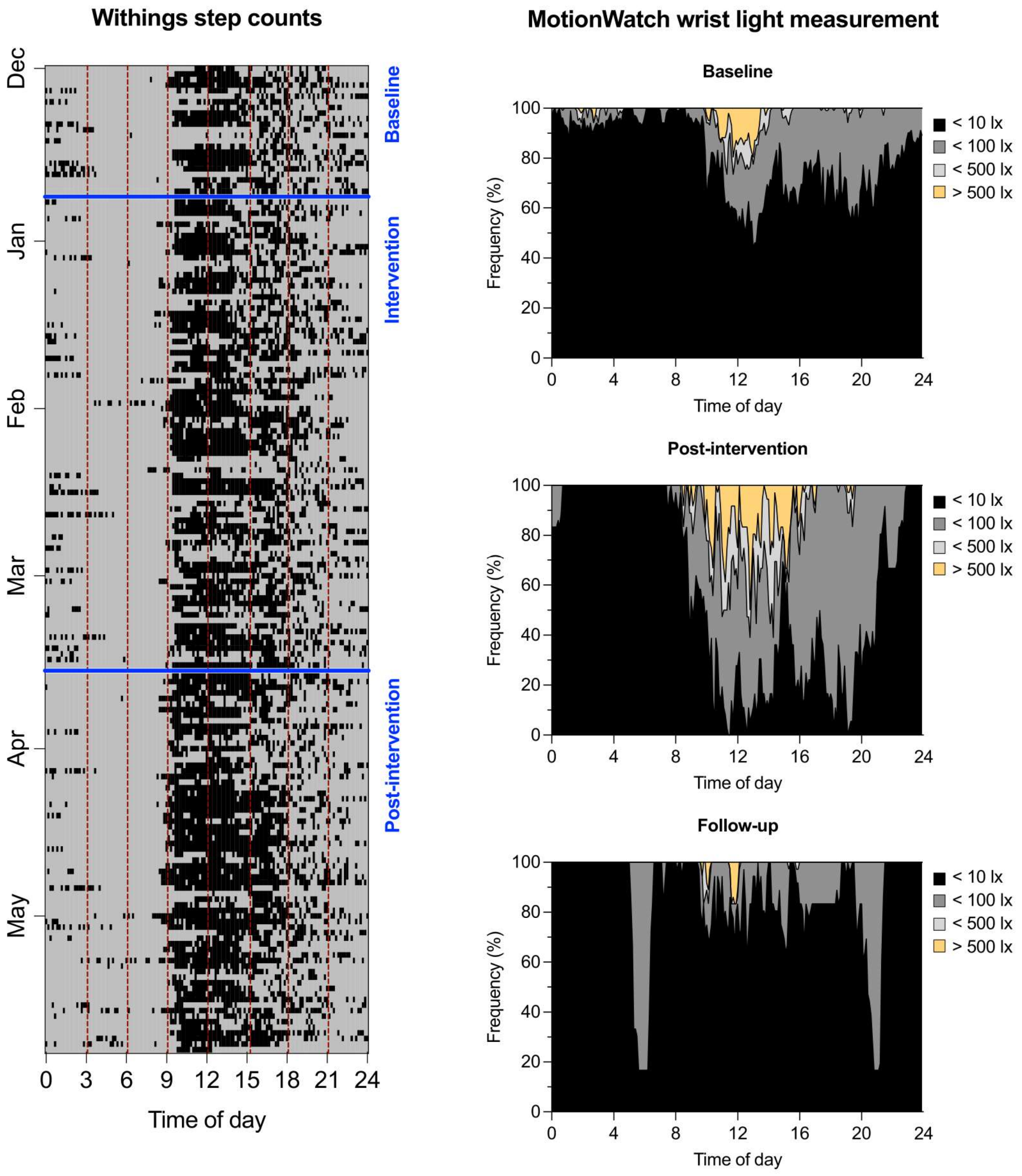

Participant 8

Bedroom

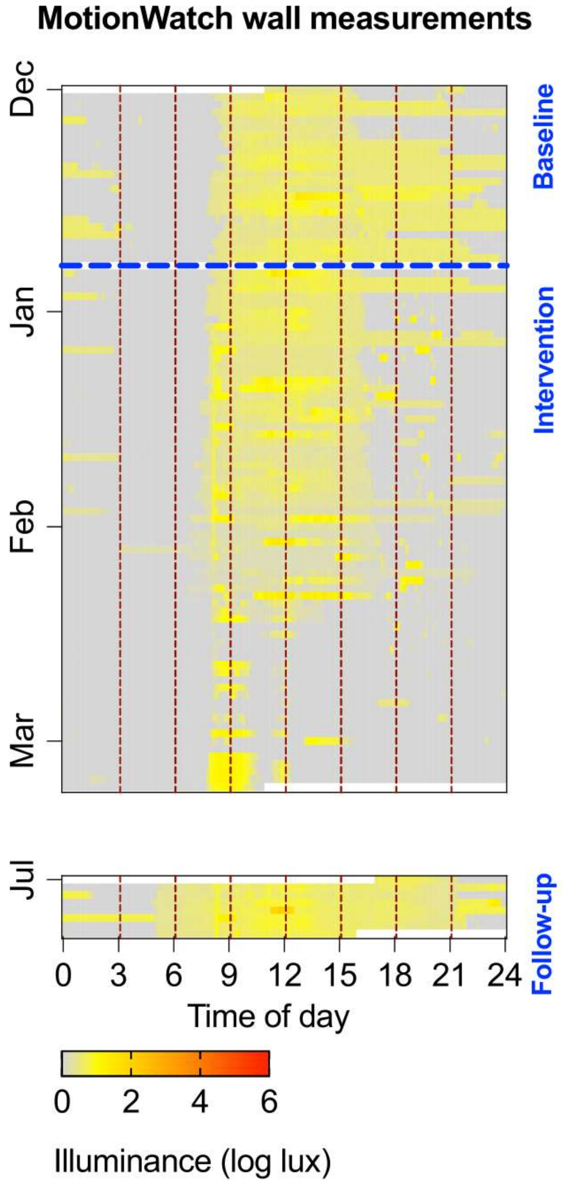

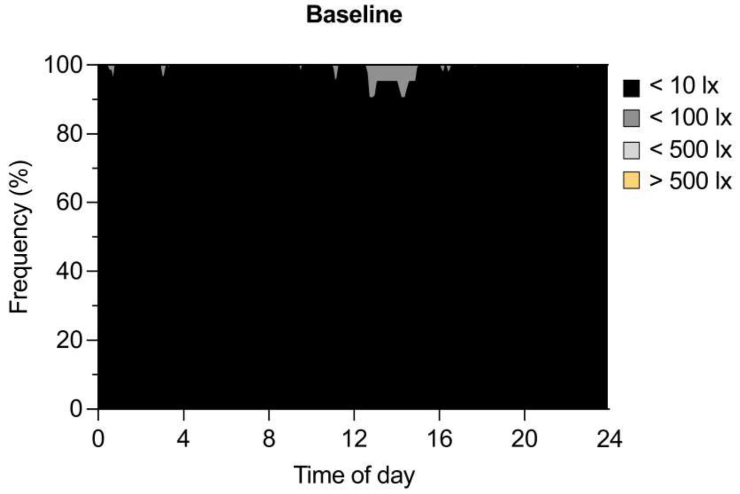

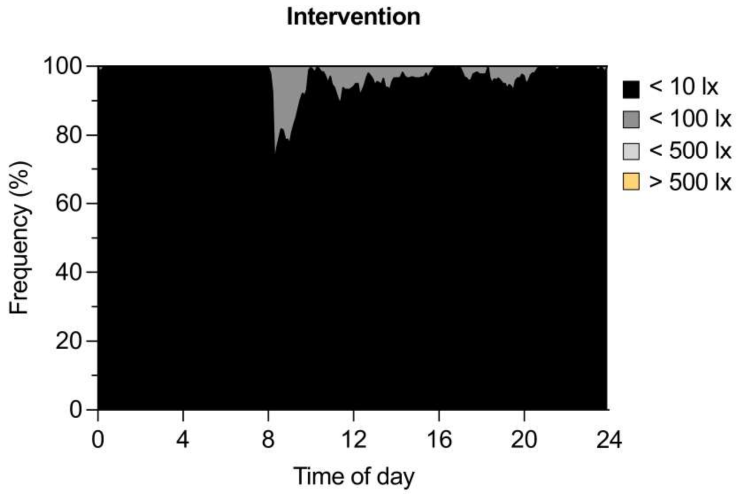

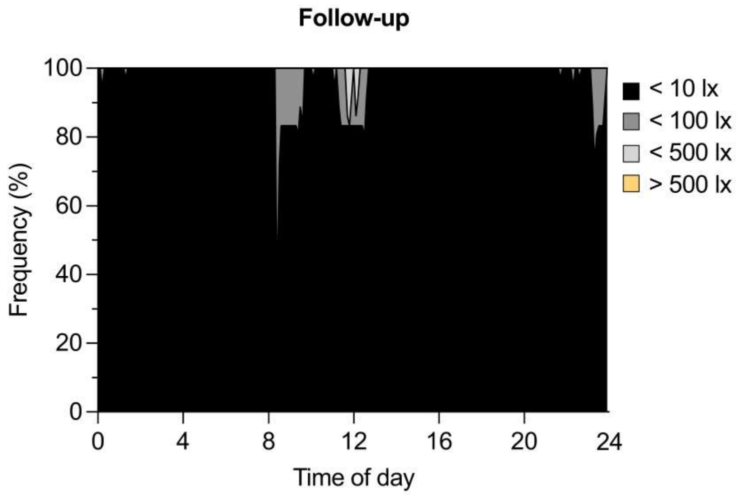

Participant 8

Living room

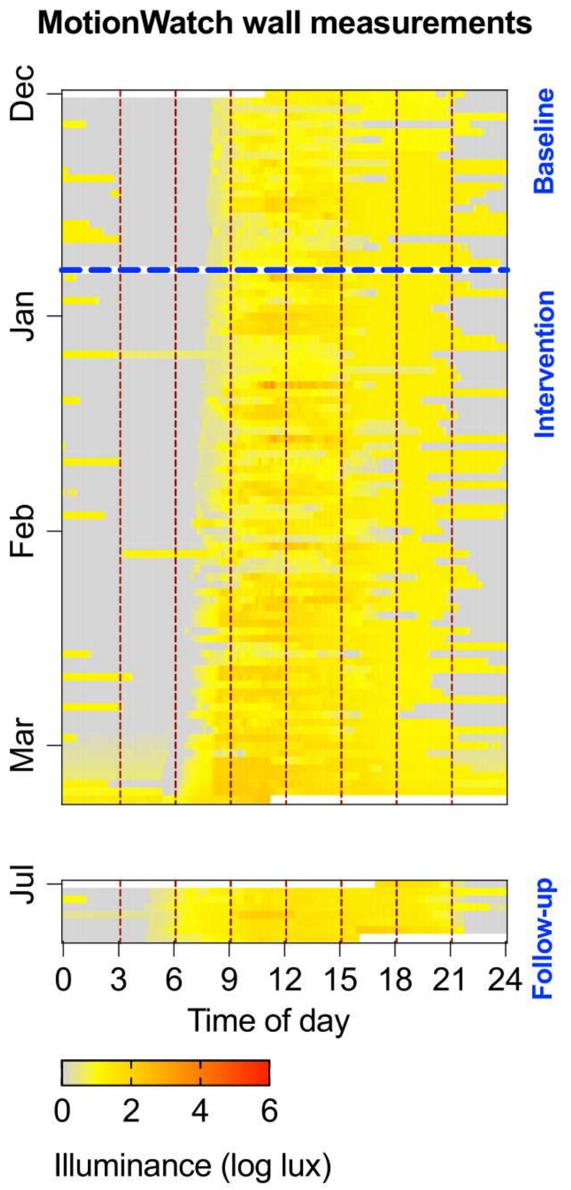

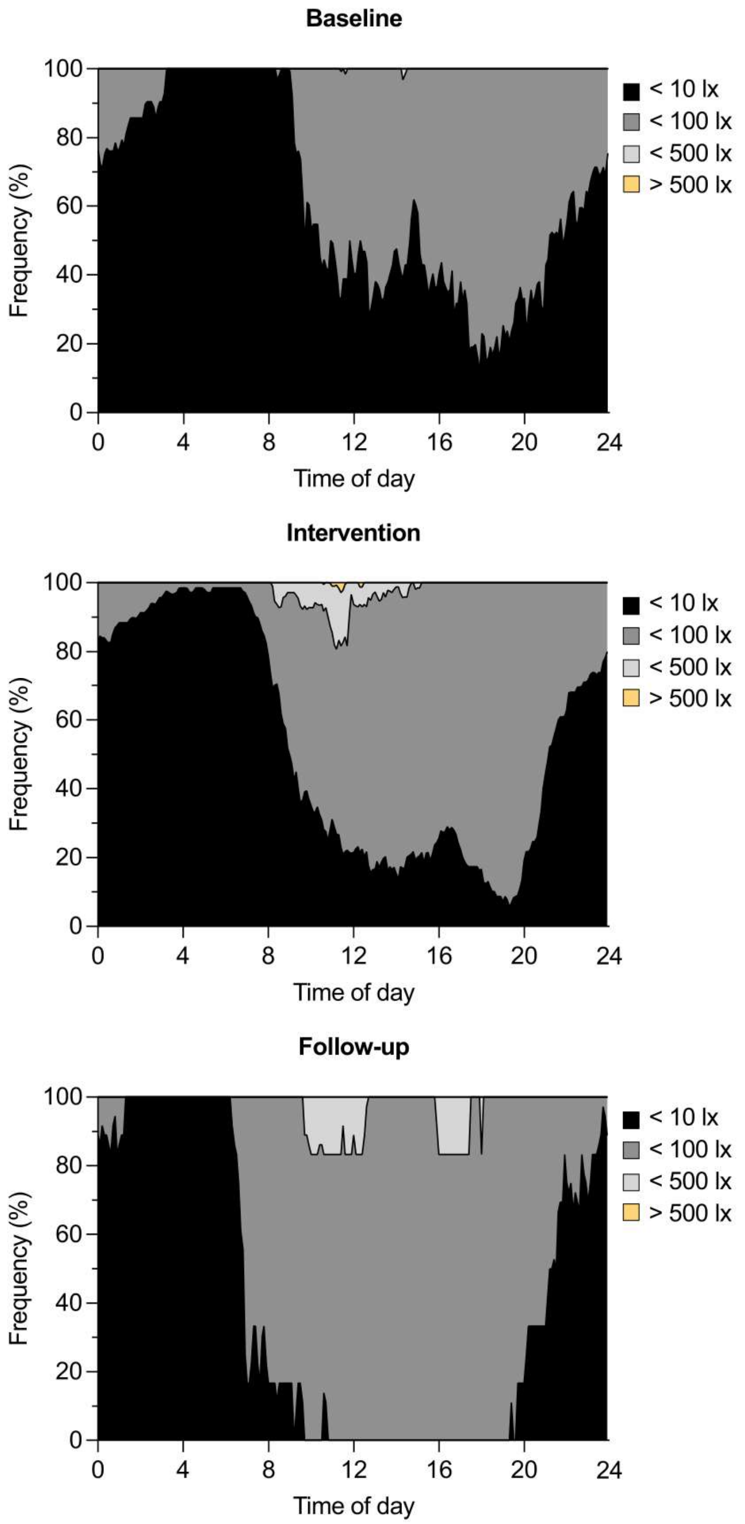

Participant 9

Withings step counts

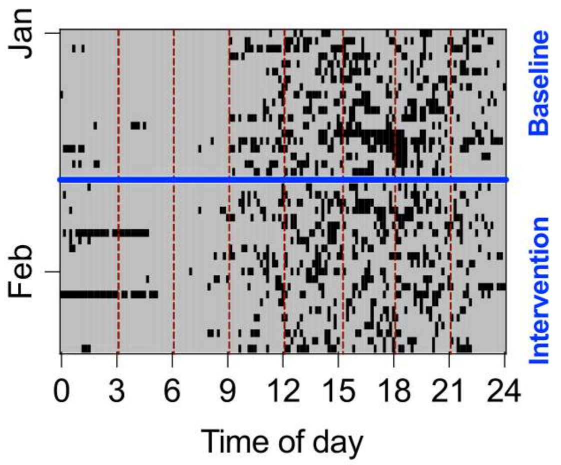

MotionWatch wrist light measurement

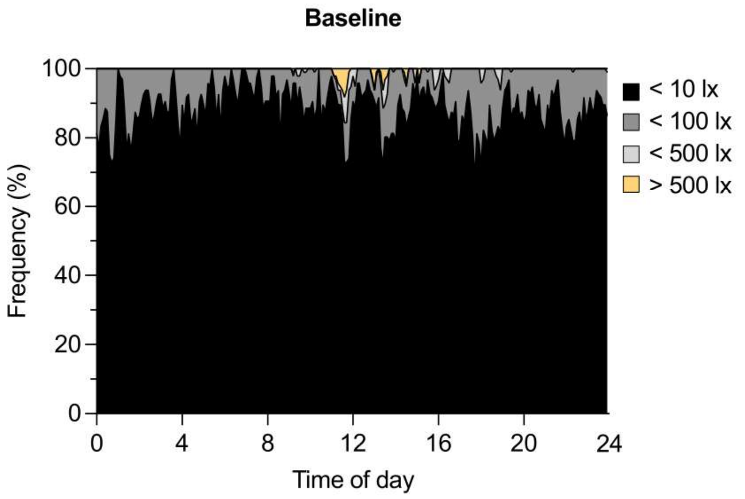

MotionWatch activity

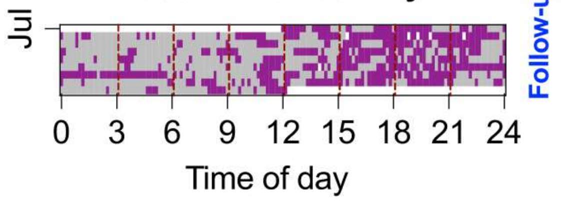

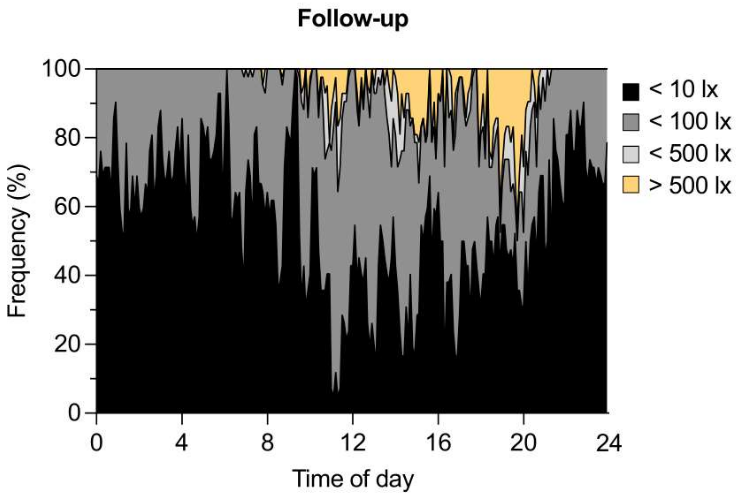

Participant 9

Bedroom

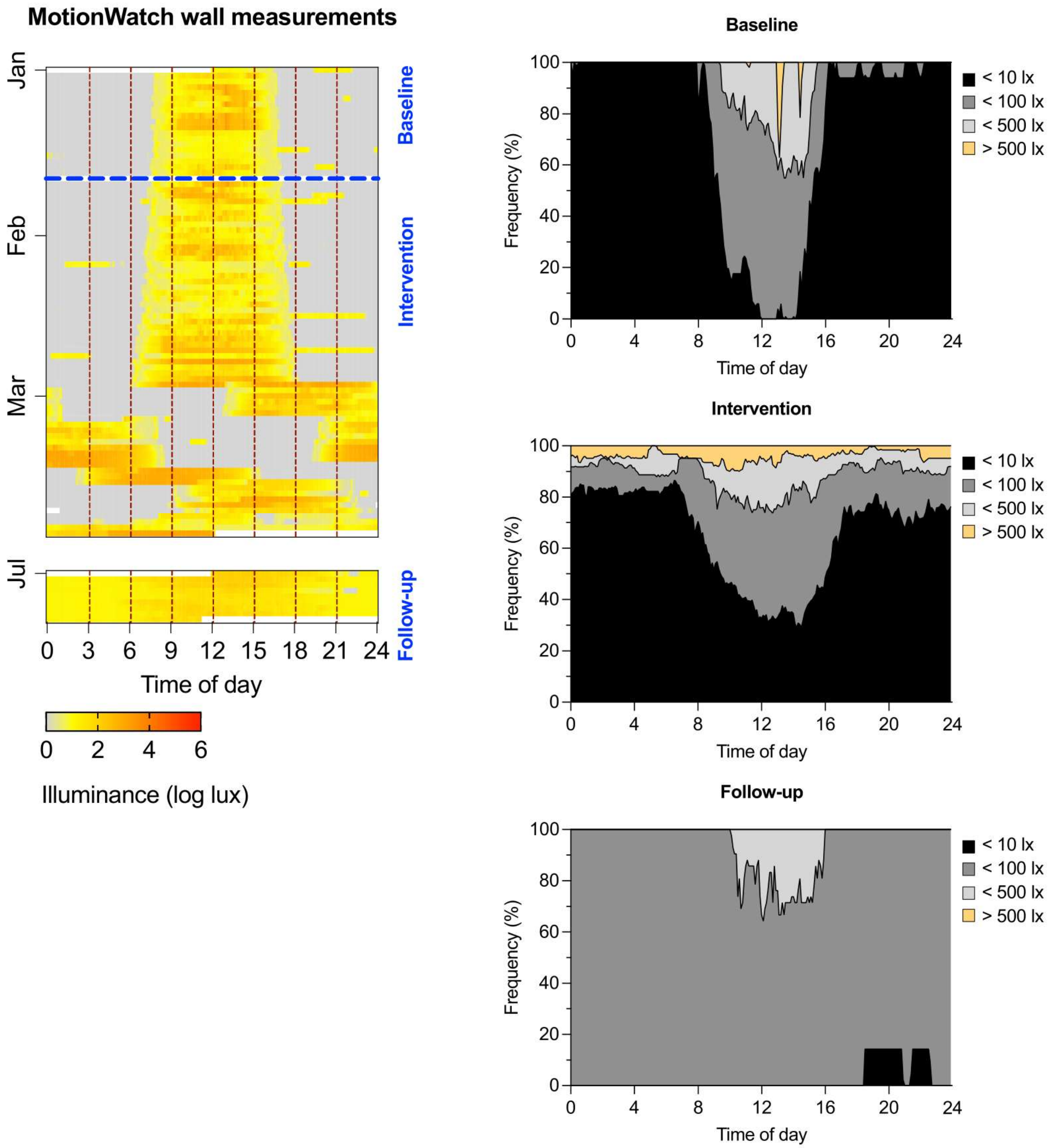

Participant 9

Living room

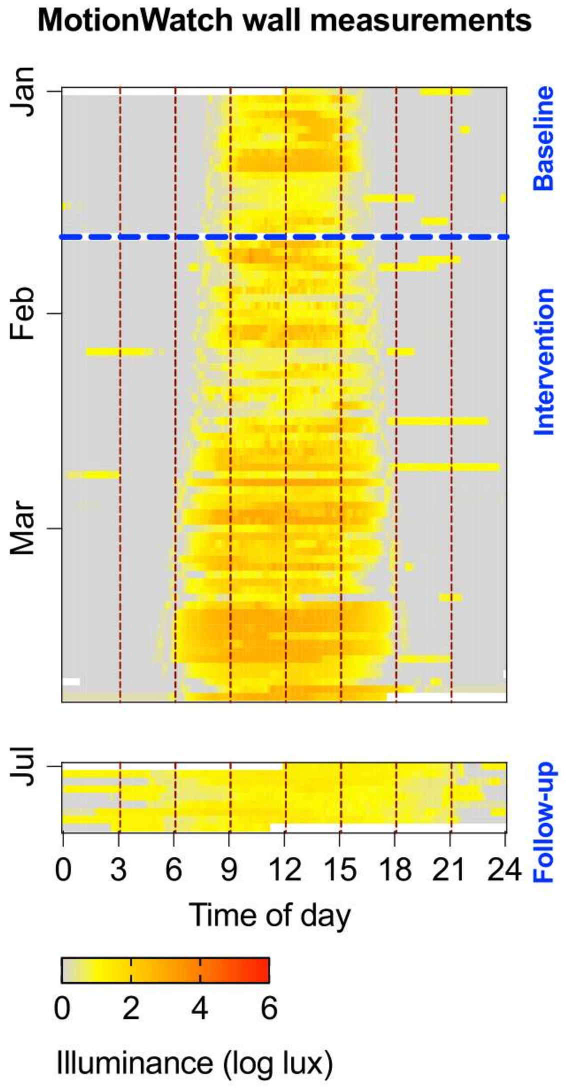

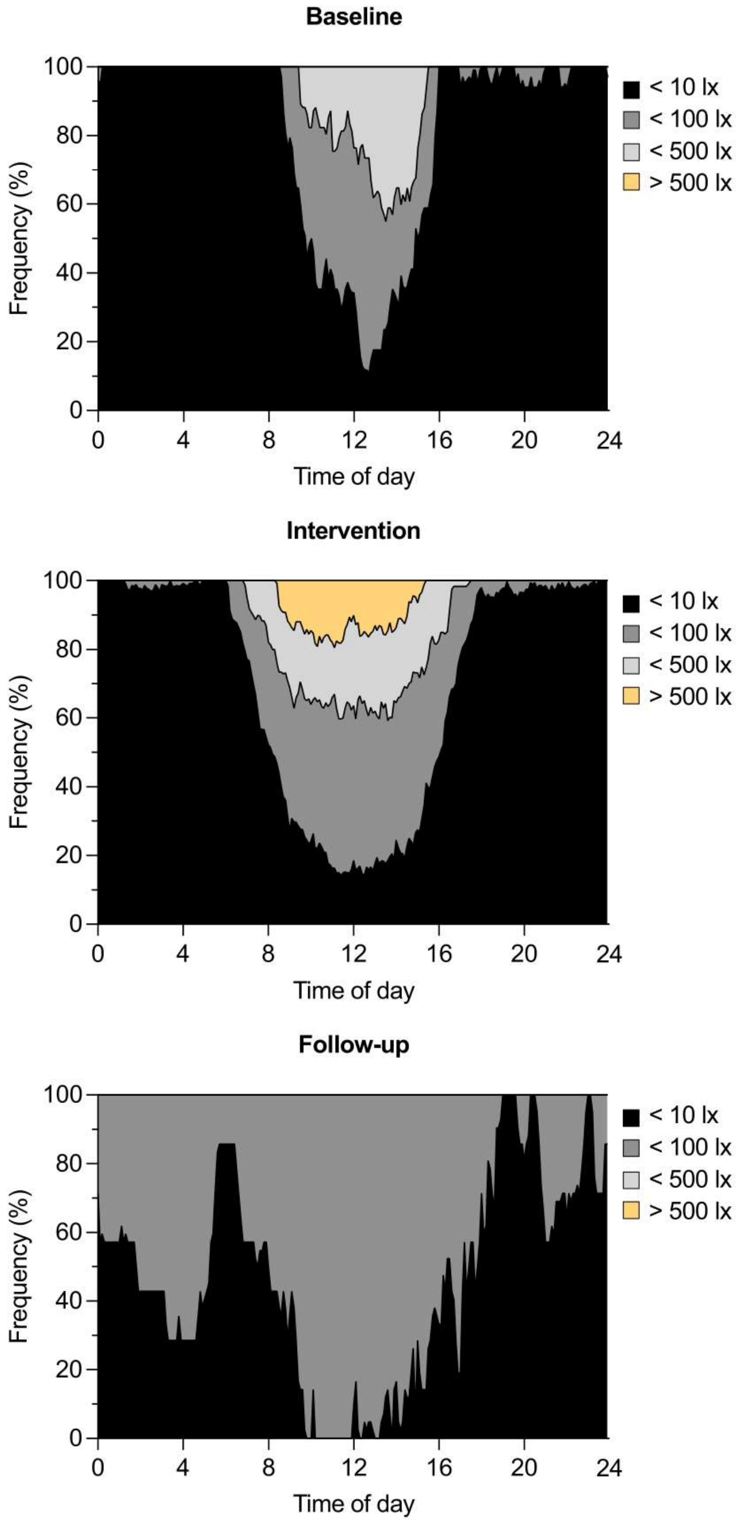

Participant 10

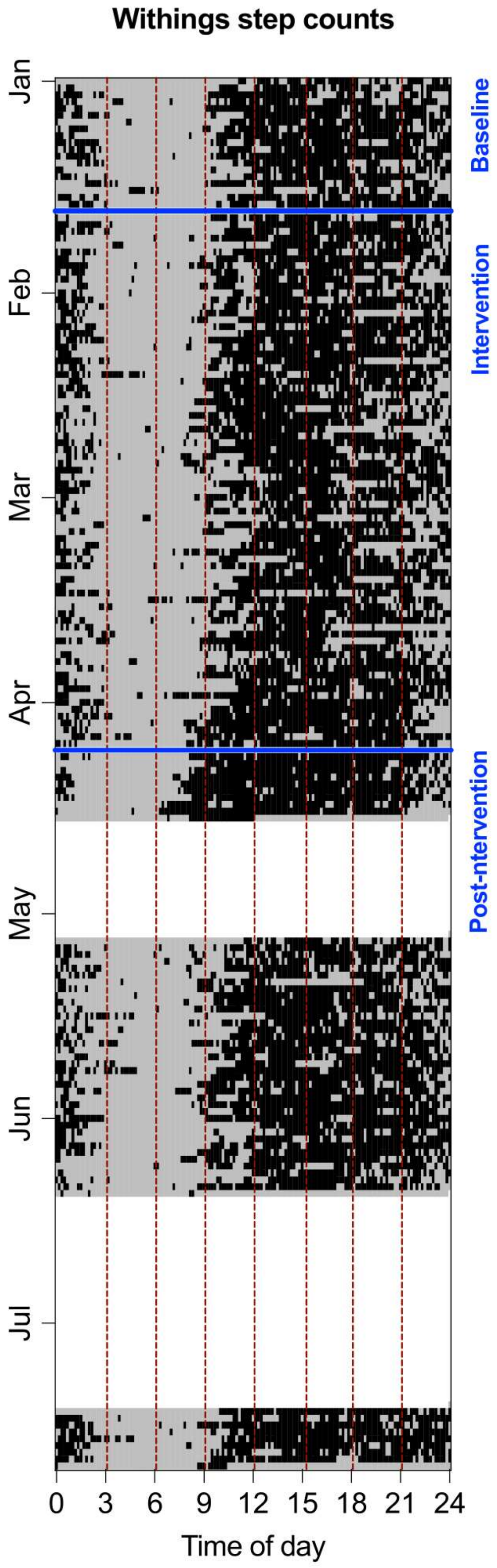

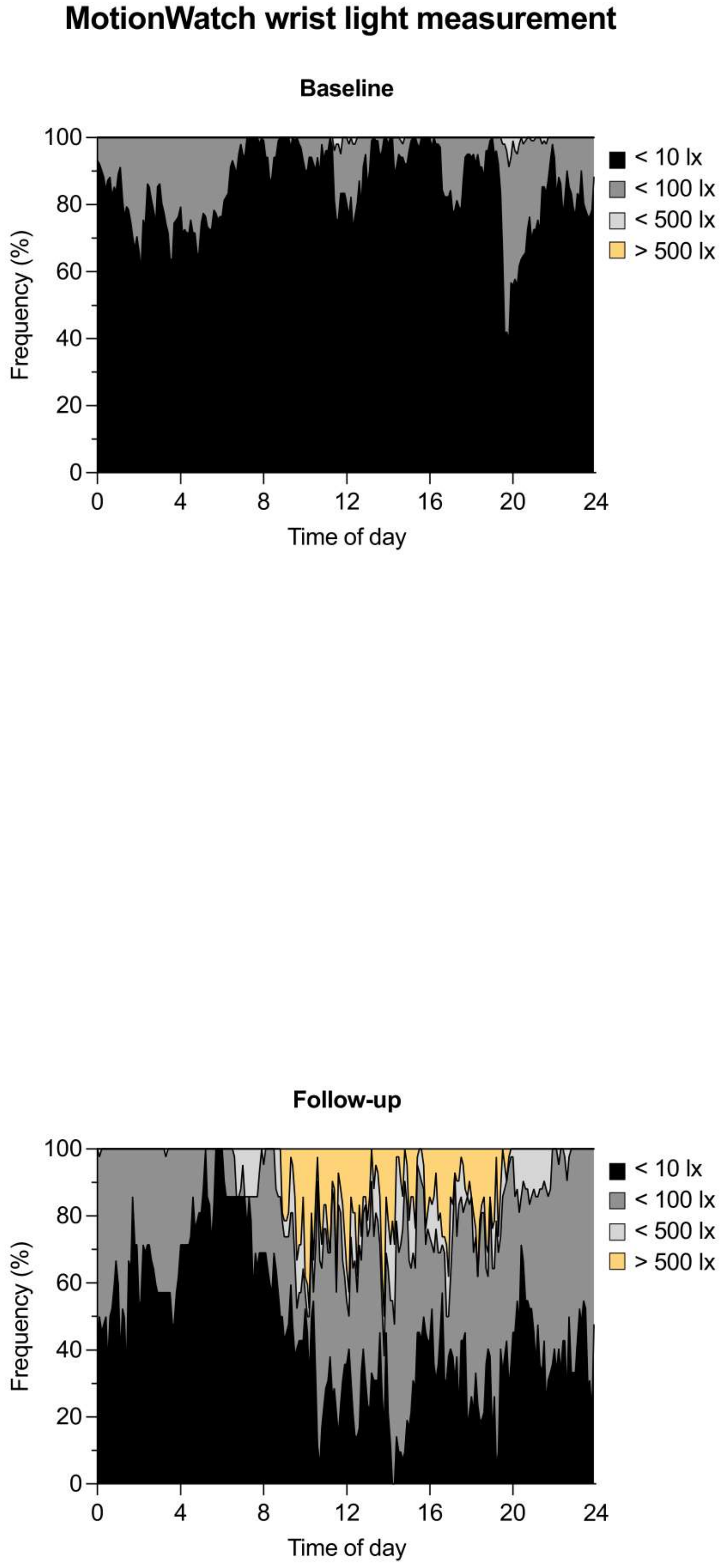

Participant 10

Bedroom

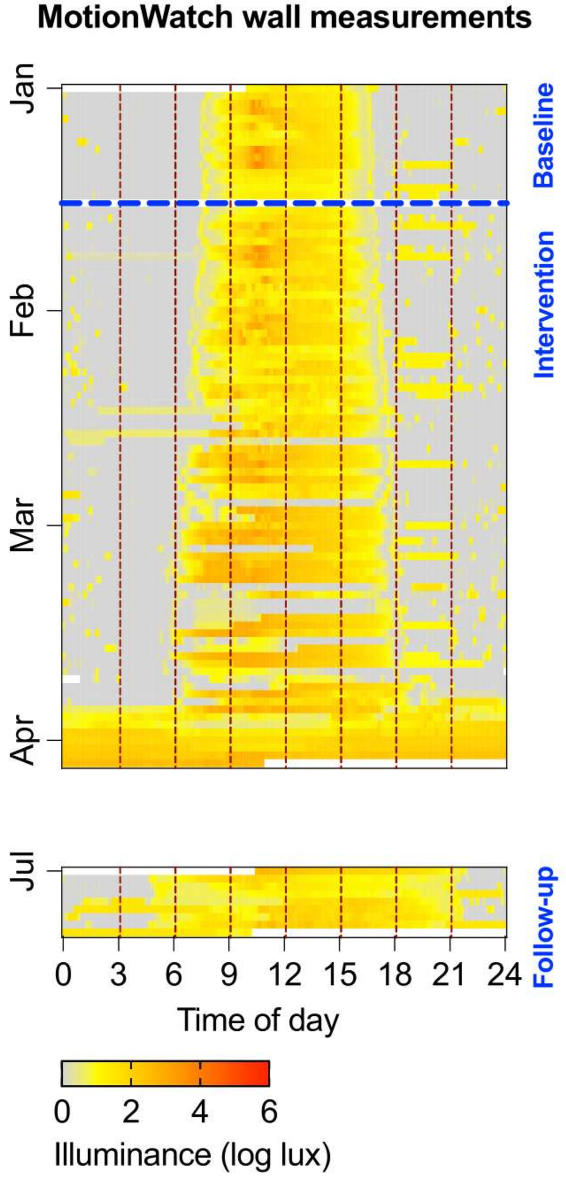

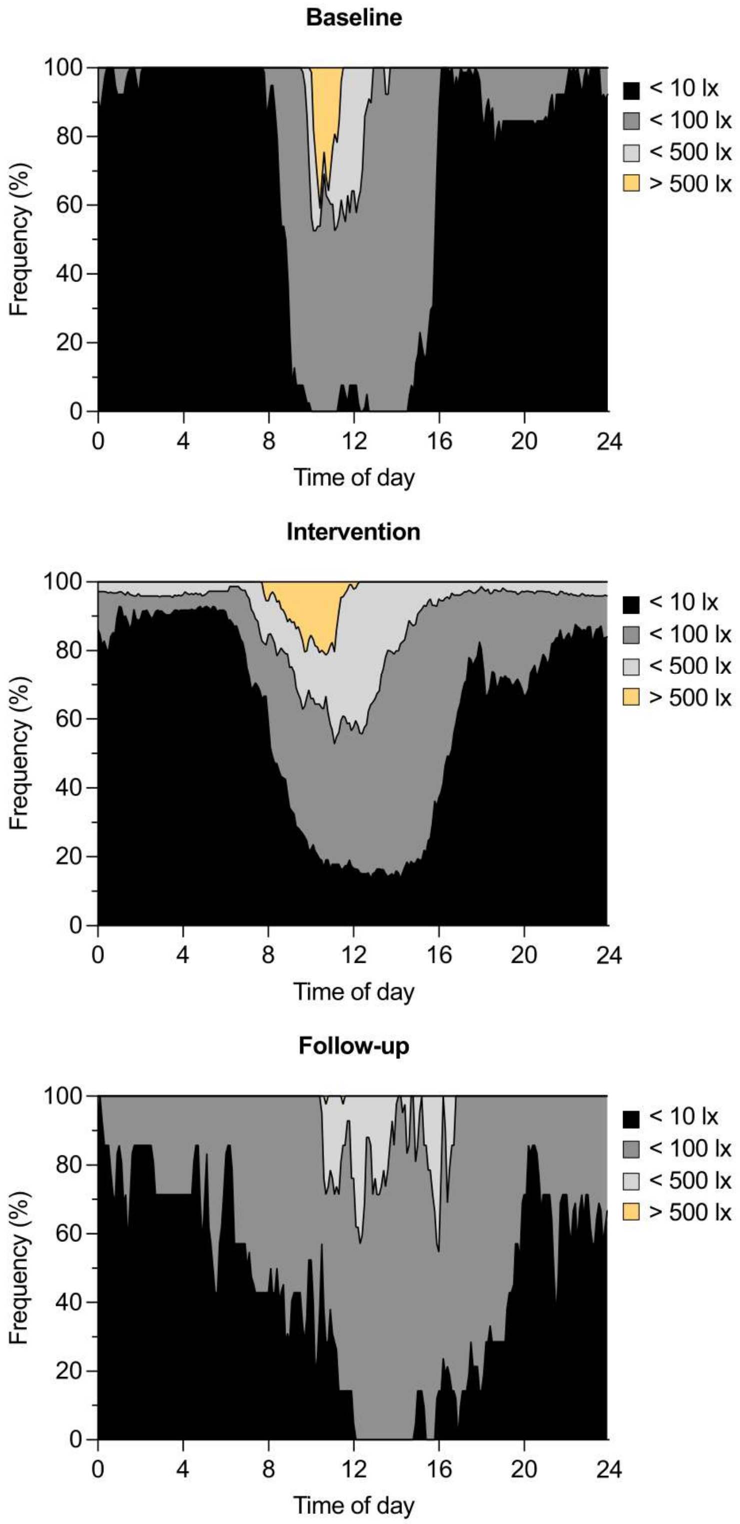

Participant 10

Living room

MotionWatch wall measurements

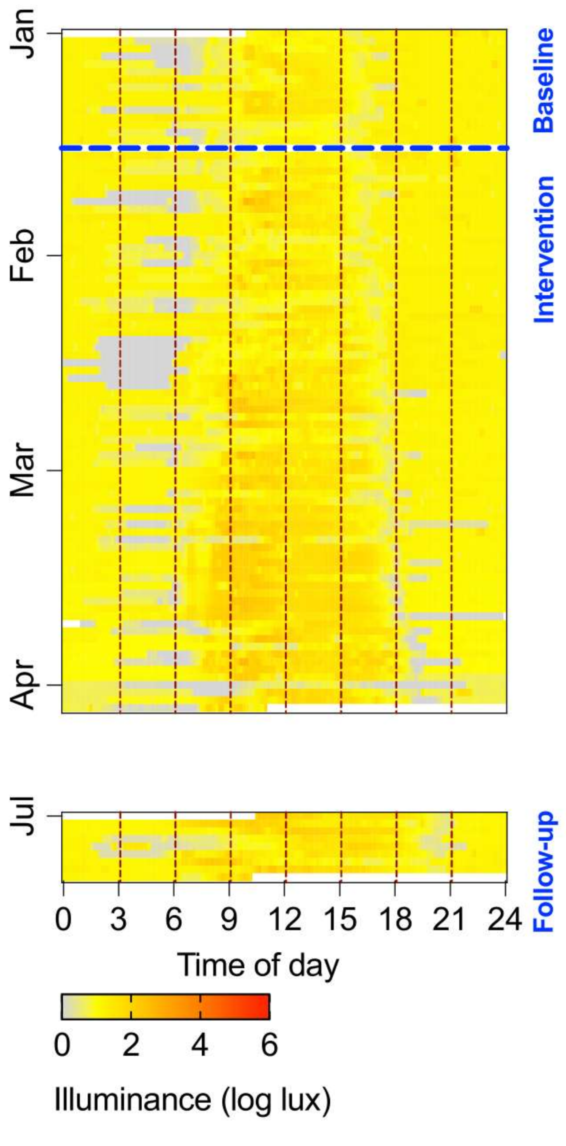

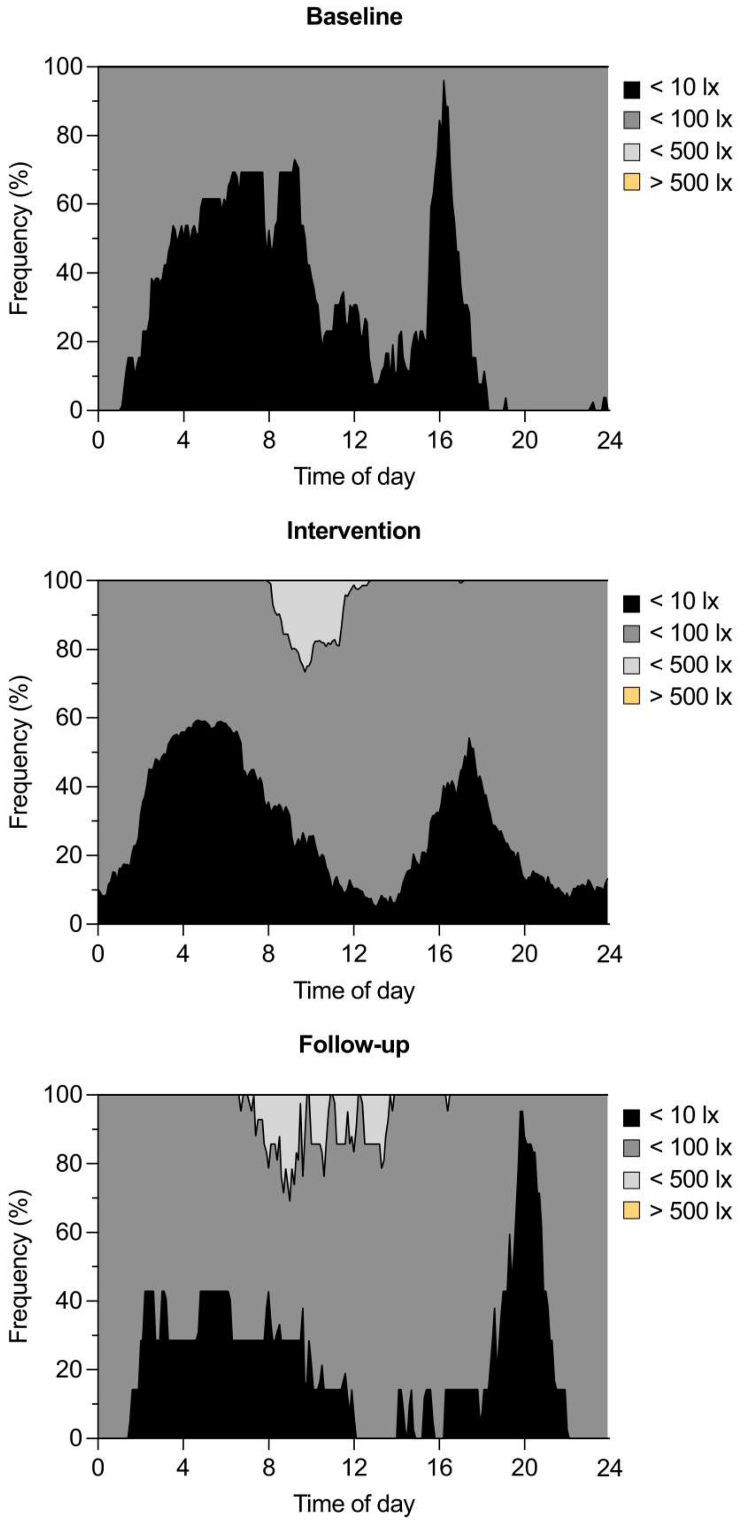

Supplement: Supplementary file 1 [file clockssleep-05-00048-s001.zip › S4 Passive monitored rest-activity and light exposure plots.pdf]
